# Supplementary material for: Multimodal switching of a redox-active macrocycle
Source: Nat Commun. 2019 Mar 1;10:1007. doi: 10.1038/s41467-019-08978-5 (PMC6397175; doi:10.1038/s41467-019-08978-5)
Supplement: Supplementary file 1 — Supplementary Information [file 41467_2019_8978_MOESM1_ESM.pdf]

Supplementary Information

Multimodal Switching of a Redox-active Macrocycle

Payne *et al*.

Supplementary Methods

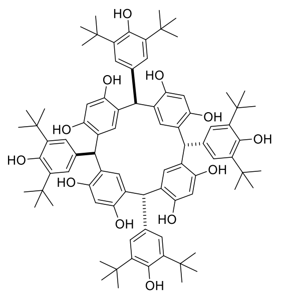

2,4,6,8-Tetrakis(3,5-di-tert-butyl-4-hydroxyphenyl)-1,3,5,7(1,3)-tetrabenzenacyclooctaphan-1^4,1^6,3^4,3^6,5^4,5^6,7^4,7^6-octa-ol (rctt-**1**). Concentrated hydrochloric acid (1 mL) and resorcinol (4.73 g, 43.0 mmol, 1.0 equiv.) were added to 3.5-di-tert-butyl-4-hydroxybenzaldehyde (10.17 g, 43 mmol, 1.0 equiv.) in ethanol (300 mL) at reflux and stirred for 3 hours. The precipitate was collected by hot filtration and washed with hot ethanol to give the desired compound as an off-white solid (10.50 g, 75%). ^{1}H NMR (300 MHz, DMSO) δ = 8.34 (s, 4H), 8.14 (s, 4H), 6.83 (s, 2H), 6.77 (s, 2H), 6.62 (s, 8H), 6.20 (s, 4H), 6.14 (s, 2H), 6.07 (s, 2H), 5.57 (s, 4H), 1.11 (s, 72H) ppm; ^{13}C NMR (75 MHz, DMSO) δ = 152.5, 152.3, 150.6, 136.6, 134.6, 124.9, 121.8, 121.0, 40.4, 33.9, 30.4 ppm; IR (ATR) v = 3550 (OH str.), 3400 (OH str.), 3200 (OH str.), 1617 (C=C str.) cm^{-1}; TOF MS ES+ m/z: 1304.7 [M]$^+$, 1305.7 [M+H]$^+$, 1306.7 [M+2H]$^+$, 1307.7 [M+3H]$^+$, 1327.7 [M+Na]$^+$, 1328.7 [M+H+Na]$^+$; HR-MS calcd. $[C_{84}H_{105}O_{12}]^+$ 1305.7601, obs. 1305.7581.

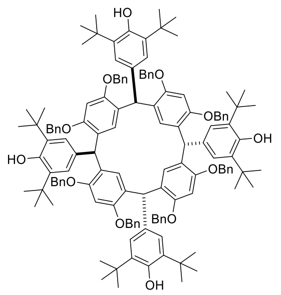

4,4',4'',4'''-(1^4,1^6,3^4,3^6,5^4,5^6,7^4,7^6-Octakis(benzyloxy)-1,3,5,7(1,3)-tetrabenzenacyclooctaphane-2,4,6,8-tetrayl)tetrakis(2,6-di-tert-butylphenol) (rctt-**2**). Benzyl bromide (4.0 mL, 33.6 mmol, 22

equiv.) was added to a stirred suspension of potassium carbonate (6.7 g, 48.5 mmol, 31 equiv.), potassium iodide (2.7 g, 16.2 mmol, 10 equiv.) and rctt-**1** (2.0 g, 1.5 mmol, 1.0 equiv.) in acetone (70 mL) followed by heating at 60 °C for 4 days under an atmosphere of dry nitrogen excluding light. The reaction mixture was cooled, poured into water and extracted with dichloromethane (3 × 100 mL). The organic fractions were combined, dried over $MgSO_4$ and the solvent was removed under reduced pressure. The residue was purified by column chromatography (SiO_2, 0-100% toluene in hexane) to afford rctt-**2** as an off-white solid (1.04 g, 34%). ^{1}H NMR (300 MHz, $CDCl_3$) δ = 7.21-7.24 (m, 8H), 7.02-7.15 (m, 28H), 6.83 (dd, J = 7.6, 1.6 Hz, 8H), 6.60-6.66 (m, 10H), 6.43 (s, 2H), 6.07 (s, 4H), 4.98 (dd, J = 20.2, 11.8 Hz, 8H), 4.83 (dd, J = 33.2, 11.9 Hz, 8H), 4.72 (s, 4H), 1.01 (s, 72H) ppm; ^{13}C NMR (75 MHz, $CDCl_3$) δ = 155.1, 154.6, 151.0, 137.9, 137.6, 134.0, 133.3, 130.9, 128.2, 128.0, 127.2, 127.0, 126.8, 126.6, 124.6, 97.5, 96.8, 69.7, 69.7, 43.0, 34.0, 30.5 ppm; IR (ATR) v = 3630 (OH str.), 3602 (OH str.), 1583 (C=C str.) cm^{-1}; TOF MS ES+ m/z: 2063.9 $[M+K]^+$, 2064.9 $[M+H+K]^+$, 2065.9 $[M+2H+K]^+$, 2066.9 $[M+3H+K]^+$; HR-MS calc. $[C_{140}H_{152}O_{12}K]^+$ 2064.0915, obs. 2064.0921.

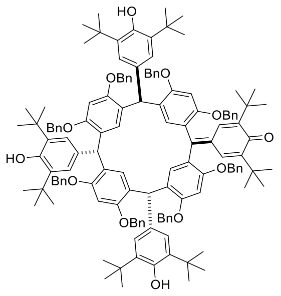

2,6-Di-*tert*-butyl-4-(1^4,1^6,3^4,3^6,5^4,5^6,7^4,7^6-octakis(benzyloxy)-4,6,8-tris(3,5-di-tert-butyl-4-hydroxyphenyl)-1,3,5,7(1,3)-tetrabenzenacyclooctaphane-2-ylidene)cyclohexa-2,5-dien-1-one (rctt-**2**-[Ox_1]). 2,3-Dichloro-5,6-dicyano-1,4-benzoquinone (5.6 mg, 0.025 mmol, 1.0 equiv.) was added to a stirred solution of rctt-**2** (50.0 mg, 0.025 mmol, 1.0 equiv.) in dichloromethane (4 mL) under a nitrogen atmosphere and the mixture was stirred for 24 hours. The reaction mixture volume was reduced by half and the reaction mixture was purified directly by preparative thin layer chromatography (SiO_2, 10% ethyl acetate in toluene) to give the title compound as a yellow solid (47.0 mg, 94%). ^{1}H NMR (300 MHz, $CDCl_3$) δ = 6.41-7.31 (m, 57H), 6.07 (s, 1H), 6.00 (d, J = 4.6 Hz, 1H), 4.51-5.07 (m, 19H), 0.85-1.32 (m, 72H) ppm; ^{13}C NMR (300 MHz, $CDCl_3$) δ = 186.2, 157.8,

156.8, 156.8, 155.7, 155.3, 155.1, 155.1, 154.5, 151.5, 151.5, 151.3, 151.0, 145.7, 145.3, 137.9, 137.7, 137.4, 137.3, 137.3, 137.2, 137.2, 137.1, 137.0, 136.8, 136.6, 136.3, 135.2, 134.4, 133.2, 133.0, 132.2, 131.8, 130.8, 130.5, 129.2, 128.9, 128.5, 128.4, 128.3, 128.3, 128.2, 128.2, 128.1, 128.1, 128.0, 128.0, 127.9, 127.6, 127.6, 127.5, 127.5, 127.4, 127.3, 127.3, 127.3, 127.2, 127.1, 127.1, 127.1, 127.0, 126.9, 126.8, 126.8, 126.7, 126.6, 126.6, 126.4, 126.3, 125.3, 124.5, 124.2, 123.0, 122.9, 100.0, 98.7, 97.8, 97.2, 71.3, 70.6, 70.2, 70.0, 69.8, 69.7, 69.6, 69.6, 43.6, 43.3, 43.1, 34.9, 34.6, 34.2, 34.2, 34.1, 34.0, 30.7, 30.6, 30.6, 30.6, 30.5, 30.3, 29.6, 29.3 ppm; IR (ATR) ν = 3611 (OH str.), 1605 (C=O str.), 1584 (C=C str.) cm^{-1}; TOF MS ES+ m/z: 2061.9 $[M+K]^+$, 2062.9 $[M+H+K]^+$, 2063.9 $[M+2H+K]^+$, 2064.9 $[M+3H+K]^+$; HR-MS calc. $[C_{140}H_{150}O_{12}K]^+$ 2062.0759, obs. 2062.0693.

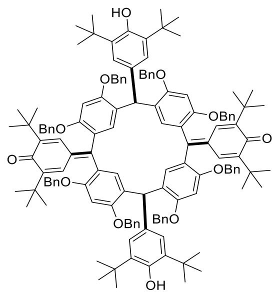

4,4'-(1^4,1^6,3^4,3^6,5^4,5^6,7^4,7^6-Octakis(benzyloxy)-4,8-bis(3,5-di-tert-butyl-4-hydroxyphenyl)-1,3,5,7(1,3)-tetrabenzenacyclooctaphane-2,6-diylidene)bis(2,6-di-tert-butylcyclohexa-2,5-dien-1-one) (rccc-**2**-[Ox_2]). 2,3-Dichloro-5,6-dicyano-1,4-benzoquinone (18 mg, 0.080 mmol, 4.0 equiv.) was added to a stirred solution of rctt-**2** (40 mg, 0.020 mmol, 1.0 equiv.) in dichloromethane (4 mL) under a nitrogen atmosphere and the resulting mixture stirred for 24 hours. The reaction mixture volume was reduced by half and the reaction mixture was purified directly by preparative thin layer chromatography (SiO_2, 10% ethyl acetate in toluene) to give the title compound as an orange solid (35 mg, 88%). ^1H NMR (300 MHz, $CDCl_3$) δ = 6.93-7.23 (m, 50H), 6.71 (s, 1H), 6.68 (s, 2H), 6.58 (s, 2H), 6.55 (s, 1H), 6.45 (s, 1H), 6.24 (s, 1H), 4.83-5.16 (m, 14H), 4.65 (dd, J = 11.5, 3.8 Hz, 4H), 1.17 (s, 18H), 1.11 (s, 18H), 1.04 (s, 18H), 1.03 (s, 18H) ppm; ^{13}C NMR (75 MHz, $CDCl_3$) δ = 186.4, 186.0, 158.0, 157.3, 156.9, 156.8, 146.9, 146.8, 146.8, 146.4, 143.0, 136.6, 136.2, 136.0, 135.9, 135.7, 135.6, 133.2, 132.3, 132.0, 131.9, 131.4, 130.6, 130.1, 129.0, 128.5, 128.4, 128.3,

128.2, 128.1, 128.0, 127.9, 127.8, 126.4, 126.3, 125.9, 125.3, 124.0, 123.5, 123.4, 122.2, 99.7, 97.5, 97.1, 71.7, 70.8, 69.8, 69.7, 35.1, 35.0, 35.0, 29.5, 29.3, 29.1 ppm; IR (ATR) ν = 1598 (C=O str.) cm^{-1}; TOF MS ES+ m/z: 2059.9 $[M+K]^+$, 2060.9 $[M+H+K]^+$, 2061.9 $[M+2H+K]^+$, 2063.9 $[M+3H+K]^+$; HR-MS calc. $[C_{140}H_{148}O_{12}K]^+$ 2060.0602, obs. 2060.0596.

Supplementary Figures

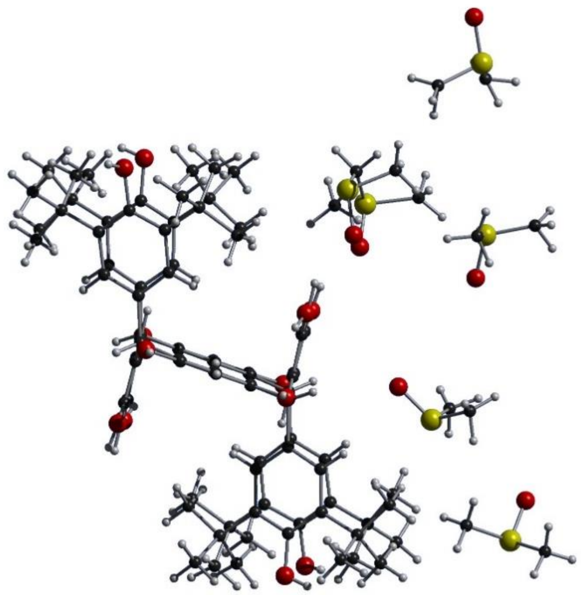

Supplementary Figure 1. X-Ray crystal structure of rctt-**1** which crystallizes with 6 molecules of dimethylsulfoxide (DMSO) in its unit cell.

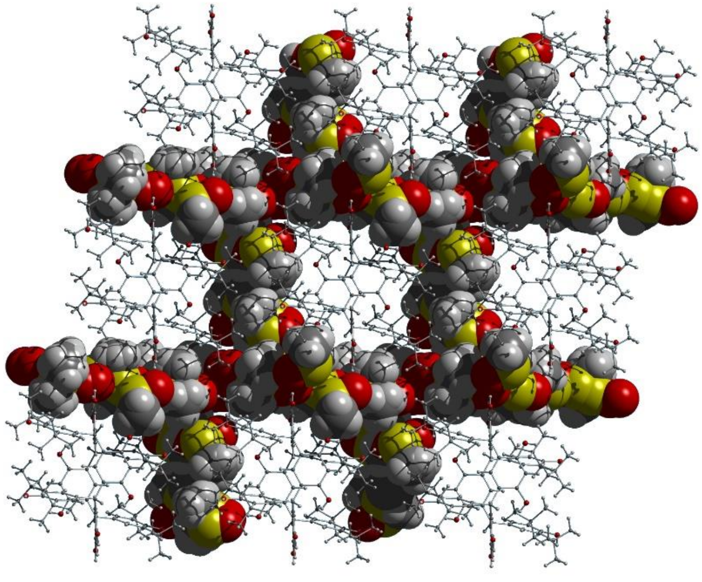

Supplementary Figure 2. Mixed representation of rctt-**1** packing with DMSO molecules depicted in space-filling format revealing its existence in the channel network of the crystals.

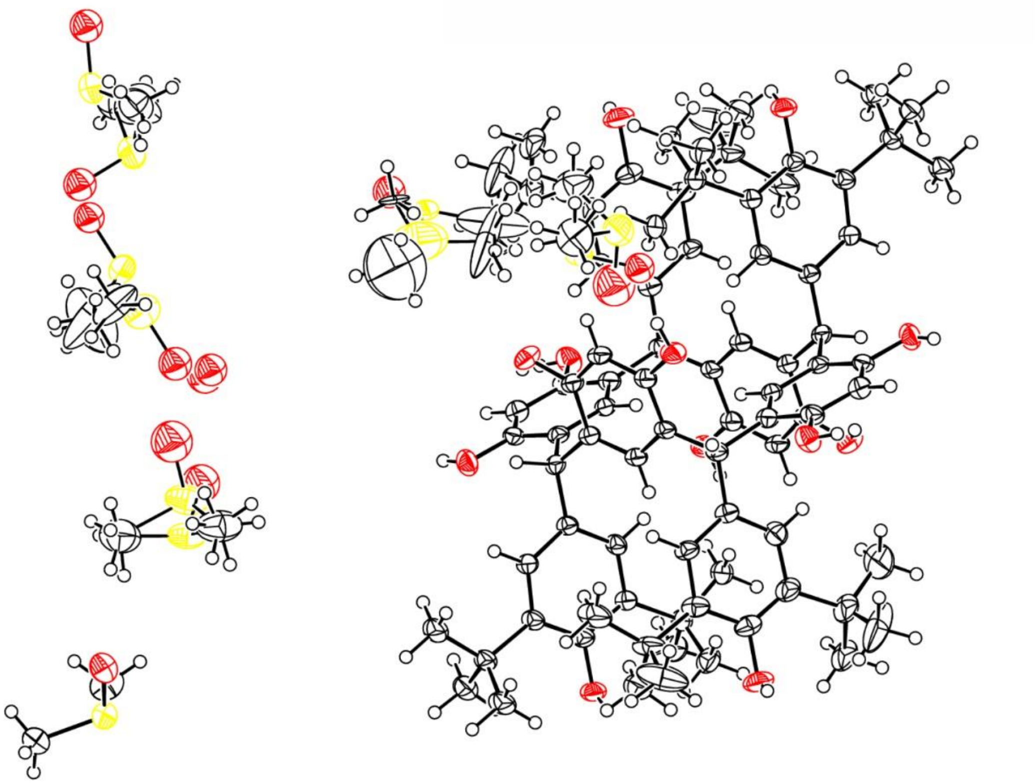

Supplementary Figure 3. ORTEP representation of rctt-**1** with ellipsoids drawn at the 50% probability level.

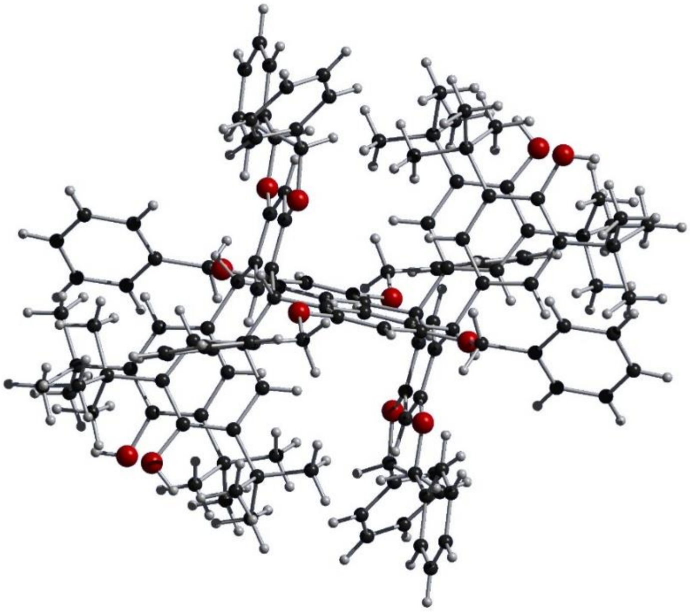

Supplementary Figure 4. X-Ray crystal structure of rctt-**2**.

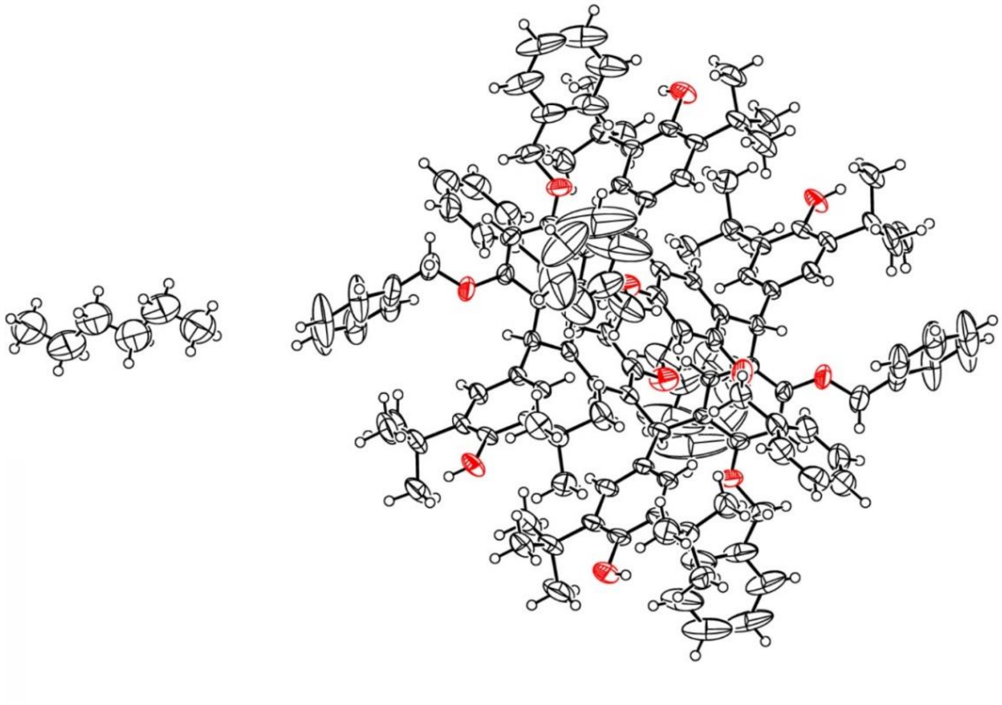

Supplementary Figure 5. ORTEP representation of rctt-**2** with ellipsoids drawn at the 50% probability level.

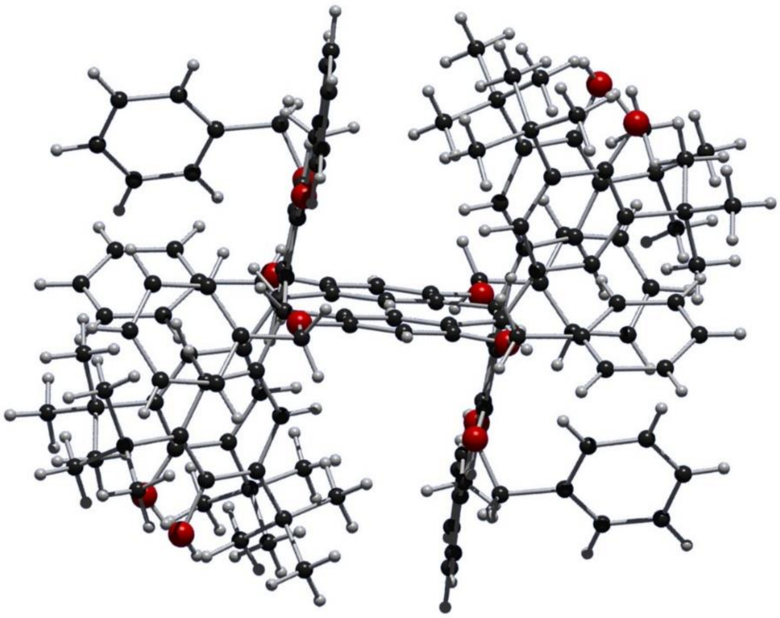

Supplementary Figure 6. X-Ray crystal structure of rctt-**2**-[Ox_1].

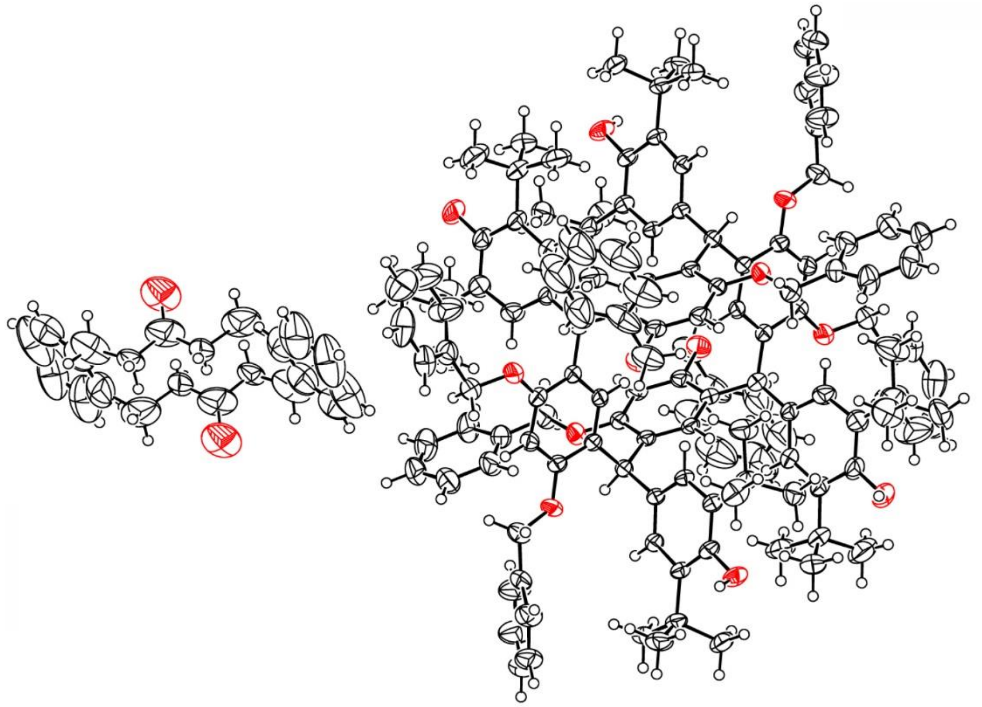

Supplementary Figure 7. ORTEP representation of rctt-**2**-[Ox_1] with ellipsoids drawn at the 50% probability level.

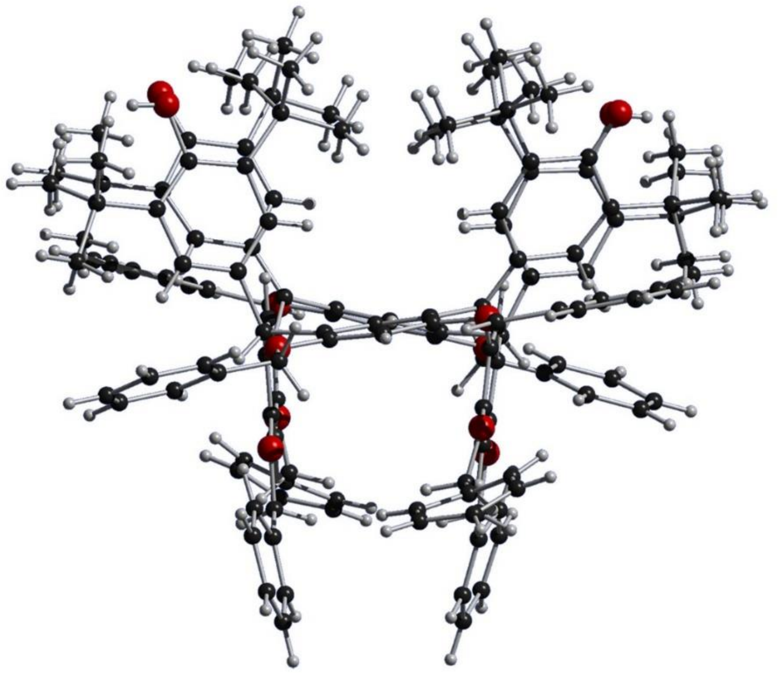

Supplementary Figure 8. X-Ray crystal structure of rccc-**2**-[Ox_2].

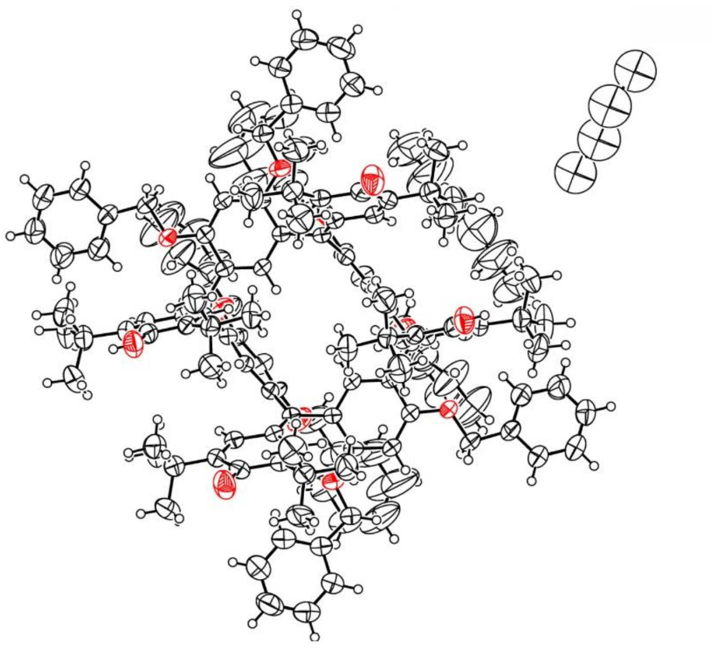

Supplementary Figure 9. ORTEP representation of rctt-**2** with ellipsoids drawn at the 50% probability level.

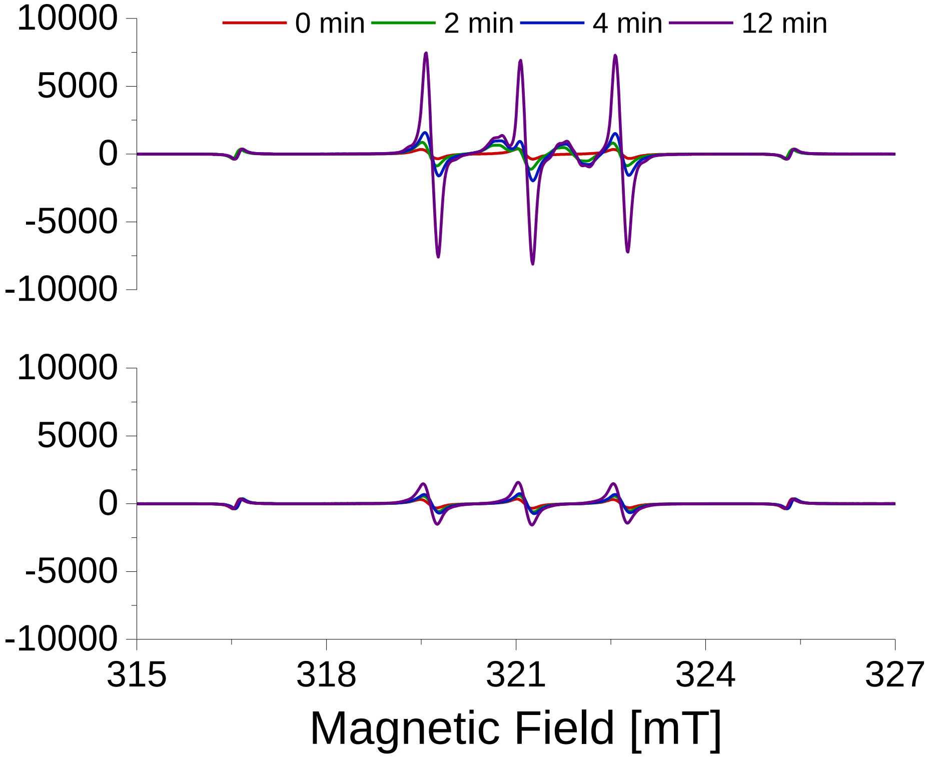

Supplementary Figure 10. Singlet oxygen $^1O_2$ ESR spectrum in the presence (top) and absence (bottom) of rctt-**2** (1.26×10^{-3} mol dm^{-3}) during photoirradiation in a capillary at 254 nm with a spin trap (2,2,6,6-tetramethylpiperidone, 0.26 mol dm^{-3}) in chloroform.

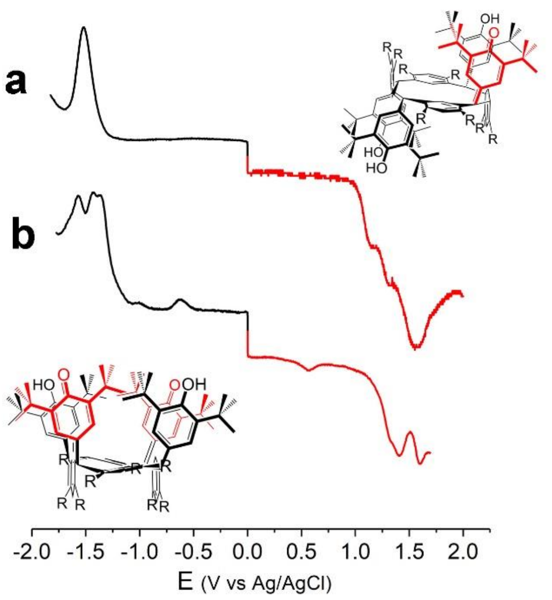

Supplementary Figure 11. Differential pulsed voltammograms for **a**, rctt-**2**-[Ox$_1$]. **b**, rccc-**2**-[Ox$_2$]. R = benzyloxy.

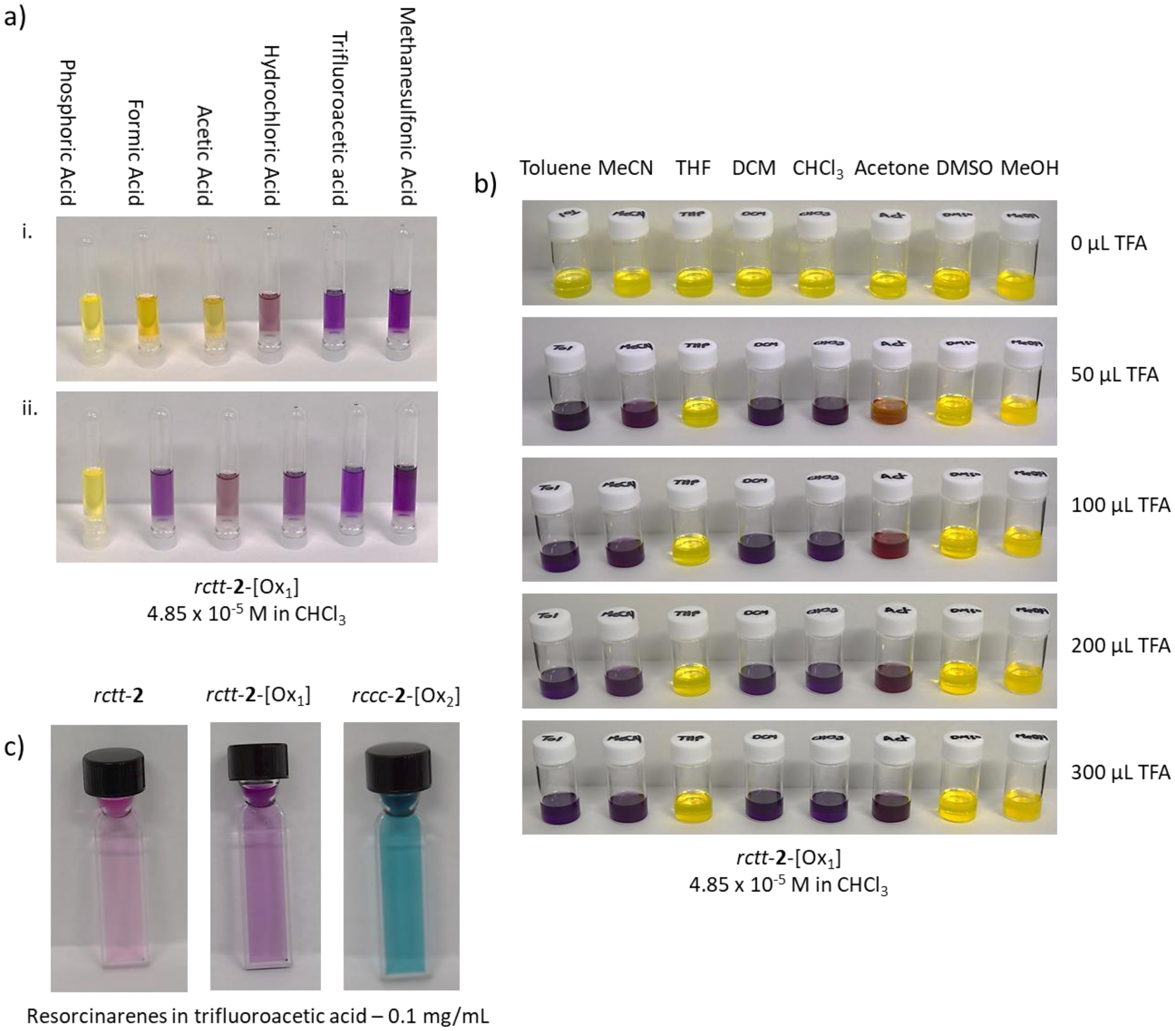

Supplementary Figure 12. (a) rctt-**2**-[Ox_1] (4.85×10^{-5} mol dm^{-3}) in chloroform in the present of various acids (i. 20 µL, ii. 100 µL) (b) rctt-**2**-[Ox_1] (4.85×10^{-5} mol dm^{-3}) in various solvents in the presence of trifluoroacetic acid (c) Appearance of the charge transfer complexes of resorcinarene derivatives in trifluoroacetic acid (0.1 mg/mL). Pink colour of rctt-**2** in trifluoroacetic acid is due to the mono-debenzylation, determined by mass spectrometric analysis.

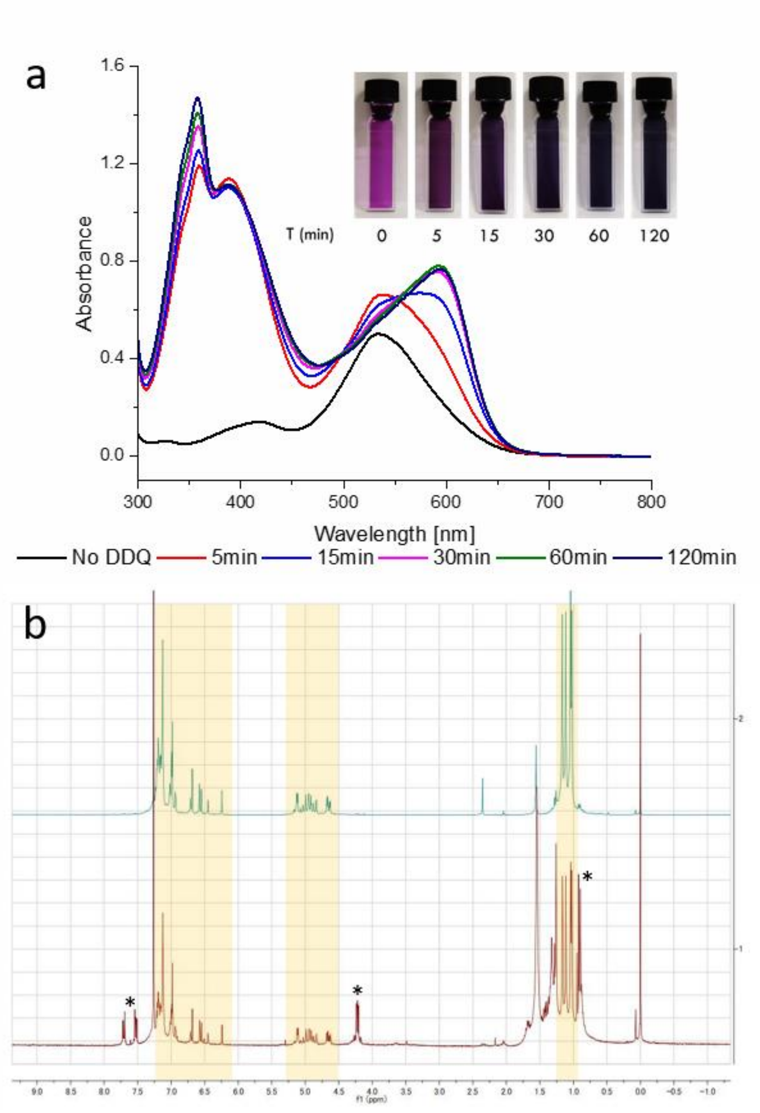

Supplementary Figure 13. Oxidation of rctt-**2**-[Ox_1] under acidic conditions. **a**, Electronic absorption spectra for 9.89×10^{-5} mol dm^{-3} rctt-**2**-[Ox_1] in TFA prior to addition of DDQ (black) and after addition of 1.5 mg DDQ (final concentration = 10.17×10^{-3} mol dm^{-3}). **b**, ^{1}H-NMR spectra of rccc-**2**-[Ox_2] (green) and the crude product obtained in **a**. Highlighted (yellow) regions indicate resonances due to rccc-**2**-[Ox_2]. Asterisk denotes unknown impurity formed during the reaction.

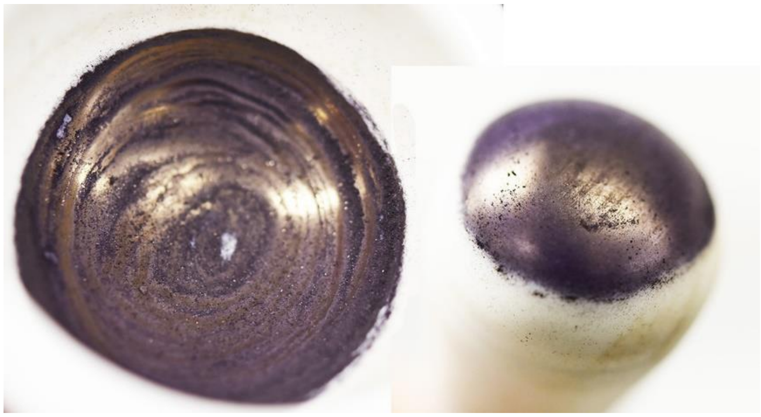

Supplementary Figure 14. Deep purple colour of the solid state charge transfer complex obtained by grinding an equimolar mixture of hydroquinone and benzoquinone. Left: in the mortar; right: deposited on pestle.

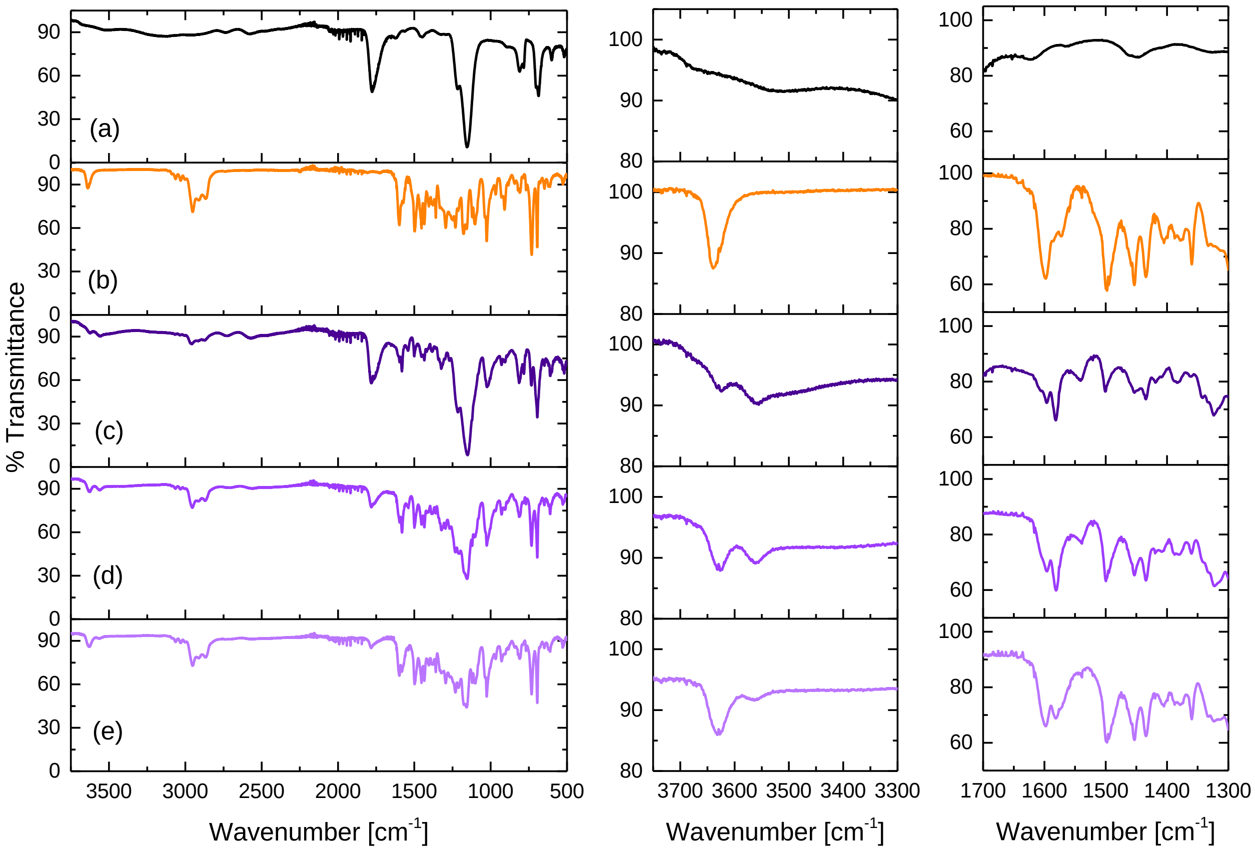

Supplementary Figure 15. Infrared spectra of (a) Trifluoroacetic acid. (b) rctt-**2**-[Ox_1]. (c) rctt-**2**-[Ox_1] after exposure to TFA vapour. (d) rctt-**2**-[Ox_1] 10 minutes after exposure to TFA vapour. (e) rctt-**2**-[Ox_1] 60 minutes after exposure to TFA vapour. After exposure to TFA, there are two notable features: appearance of a new lower frequency O-H stretching vibration (~3550 cm^{-1}) assigned to the OH groups of the hemiquinhydrone complex (see center panel) and a shift to lower frequency of the hemiquinonoid C=O stretching vibration (~1600 cm^{-1} to ~1580 cm^{-1}) due to charge transfer from phenol to quinone moiety.

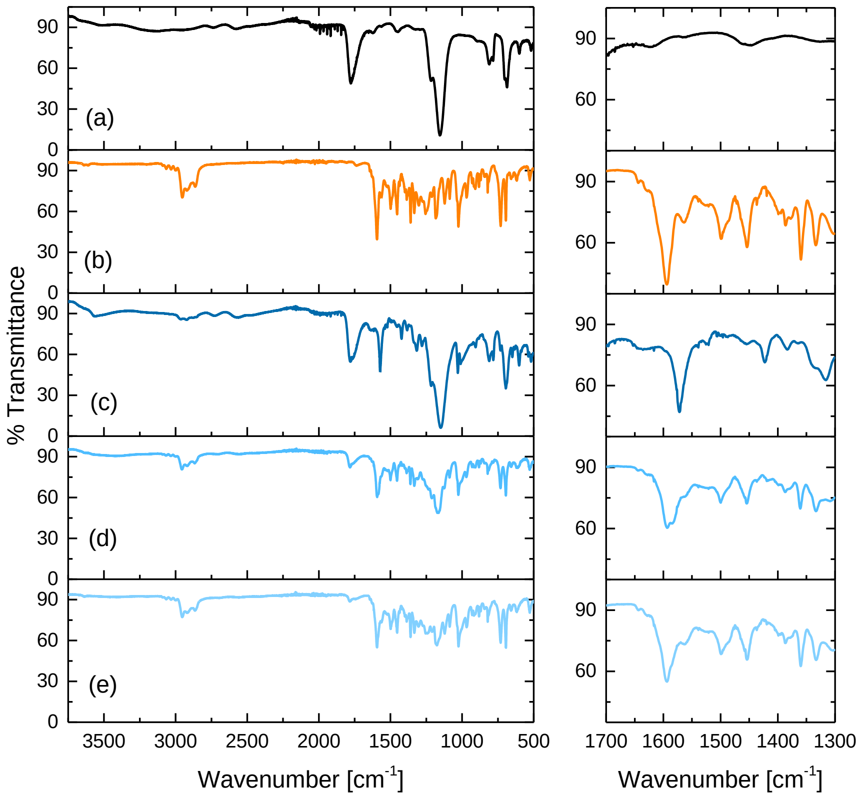

.

Supplementary Figure 16. Infrared spectra of (a) Trifluoroacetic acid. (b) rccc-**2**-[Ox$_2$]. (c) rccc-**2**-[Ox$_2$] after exposure to TFA vapour. (d) rccc-**2**-[Ox$_2$] 5 minutes after exposure to TFA vapour. (e) rccc-**2**-[Ox$_2$] 45 minutes after exposure to TFA vapour. Similar variation occurs in the hemiquinonoid C=O stretching vibration as for rctt-**2**-[Ox$_1$] (20 cm-1 shift to lower frequency) but a single OH stretching vibration emerges at ~3550 cm^{-1} since all substituents are involved in hemiquinhydrone formation in the presence of trifluoroacetic acid (see panel (c)).

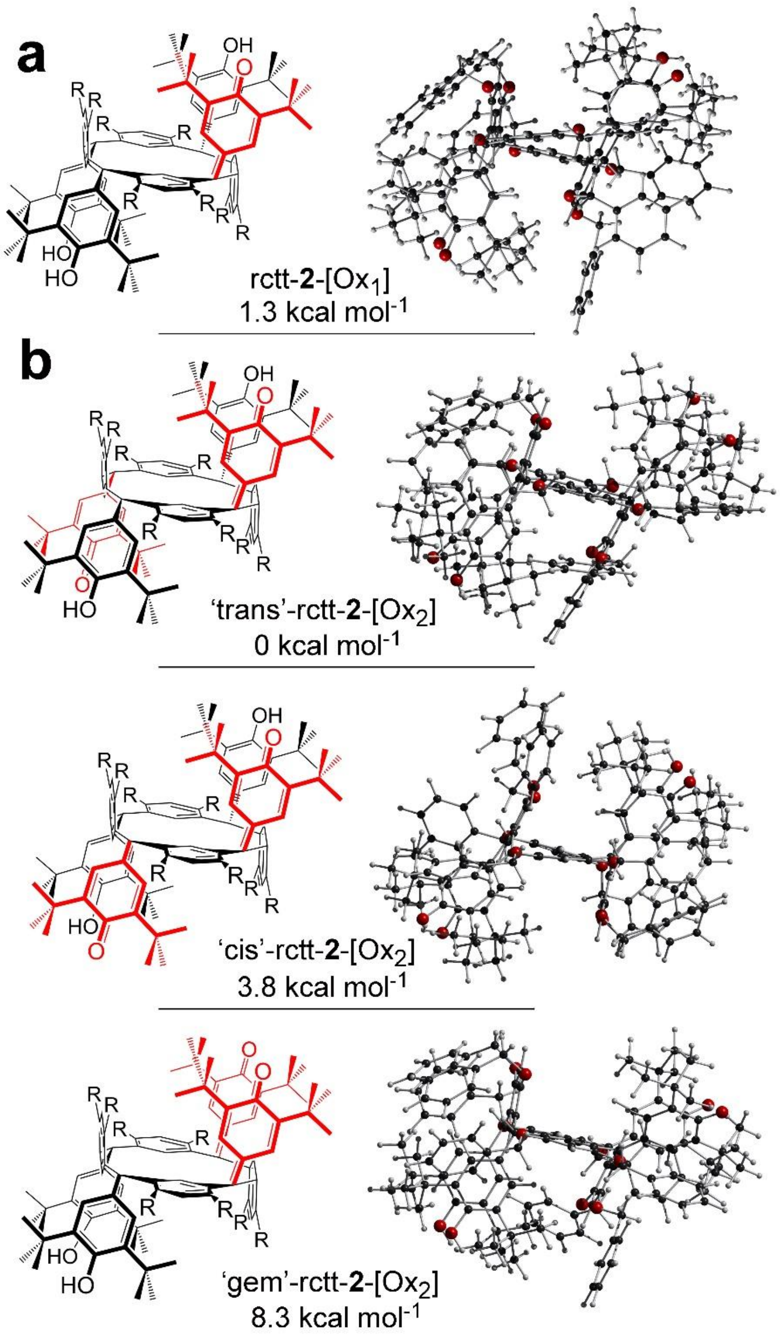

Supplementary Figure 17. DFT energy minimized structures for **a**, rctt-**2**-[Ox_1] and **b**, rctt-**2**-[Ox_2] with quinone substituents oriented (i) 'trans', (ii) 'cis' and (iii) 'geminal' (terms used analogously with alkenes). Note that energy differences in Supplementary Fig. 17 and 18 are based on compounds with the same numbers of atoms. That is, [Ox_1]-type compounds (Supplementary Figures 17(a), Fig. 18(a)) are considered separately from [Ox_2]-type compounds (Supplementary Figures 17(b), Fig. 18(b)).

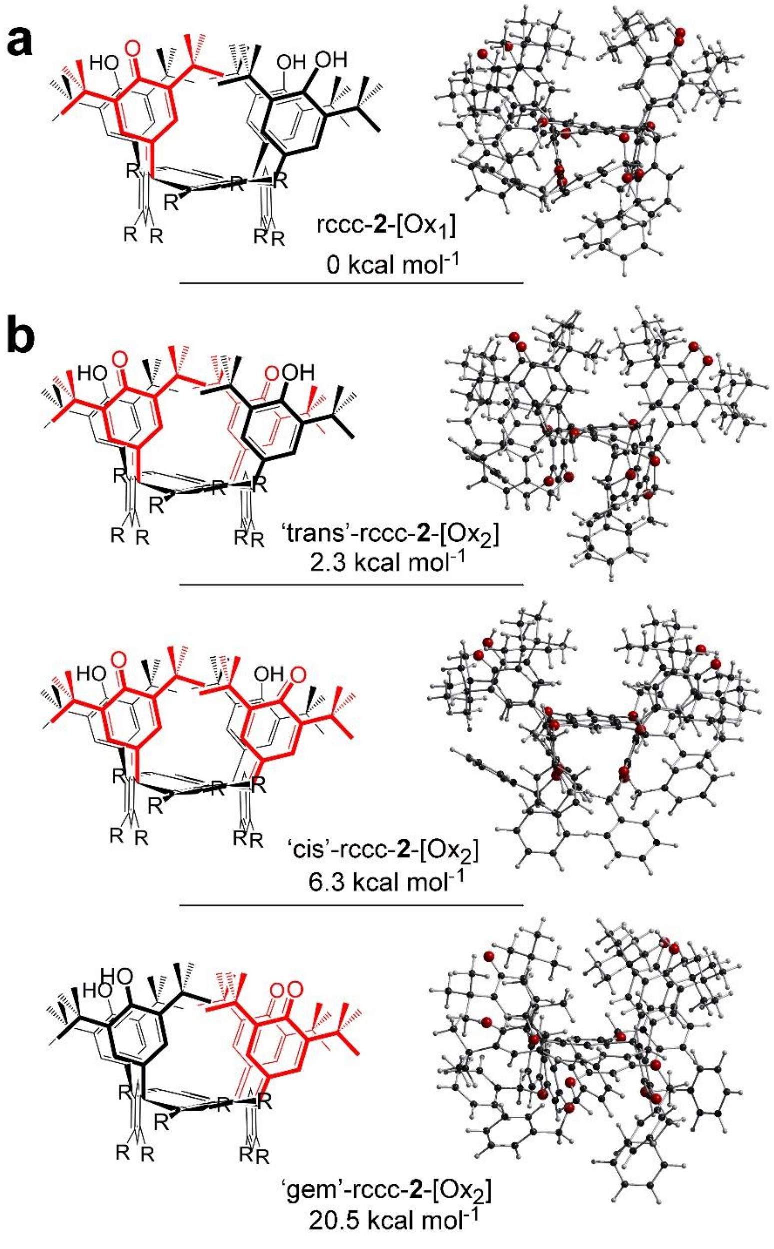

Supplementary Figure 18. DFT energy minimized structures for **a**, rccc-**2**-[Ox_1] and **b**, rccc-**2**-[Ox_2] with quinone substituents oriented (i) 'trans', (ii) 'cis' and (iii) 'geminal' (terms used analogously with alkenes). Note that energy differences in Supplementary Fig. 17 and 18 are based on compounds with the same numbers of atoms. That is, [Ox_1]-type compounds (Supplementary Figures 17(a), Fig 18(a)) are considered separately from [Ox_2]-type compounds (Supplementary Figures 17(b), Fig. 18(b)).

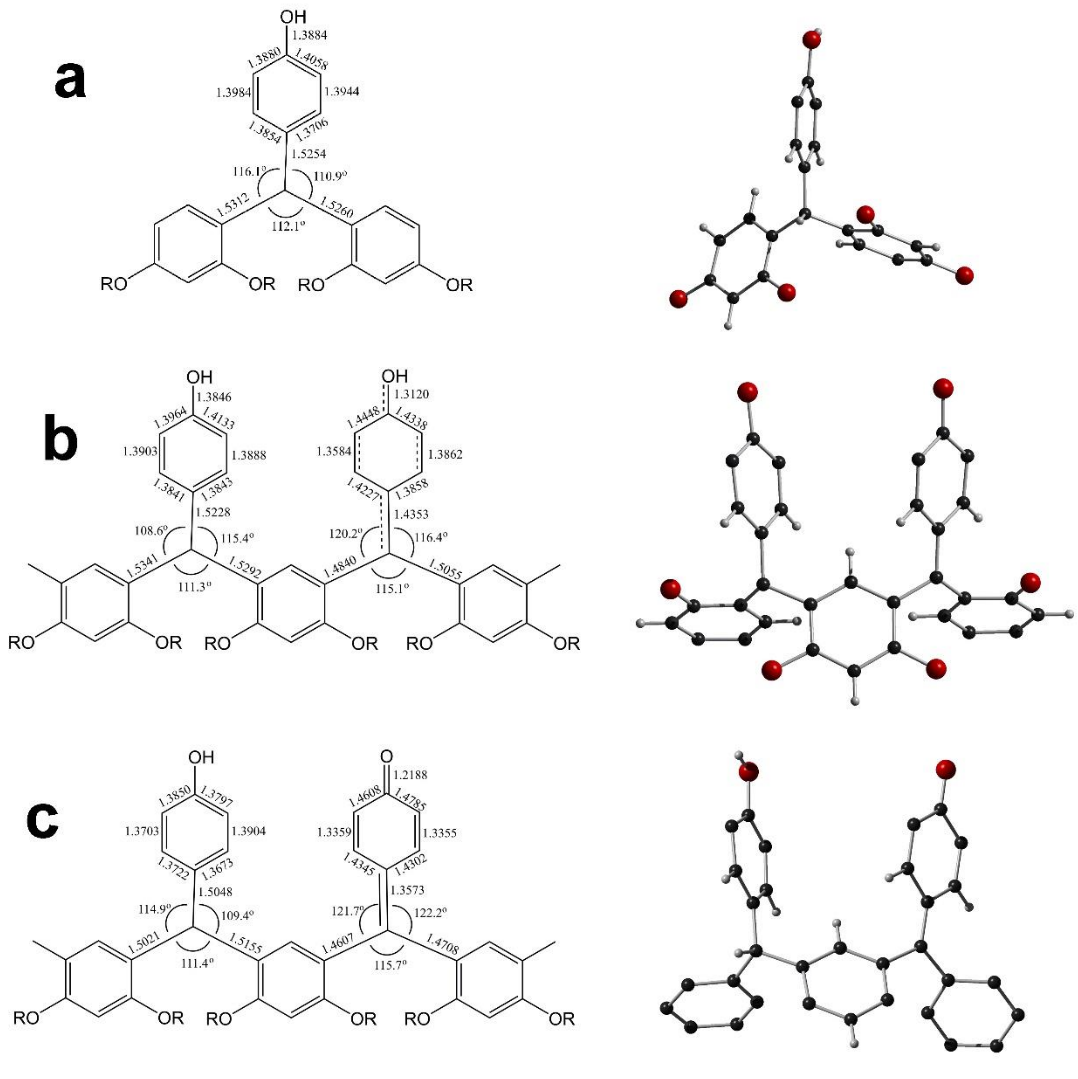

Supplementary Figure 19. Bond lengths and geometries for (a) rctt-**2**, (b) rctt-**2**-[Ox_1] and (c) rccc-**2**-[Ox_2].

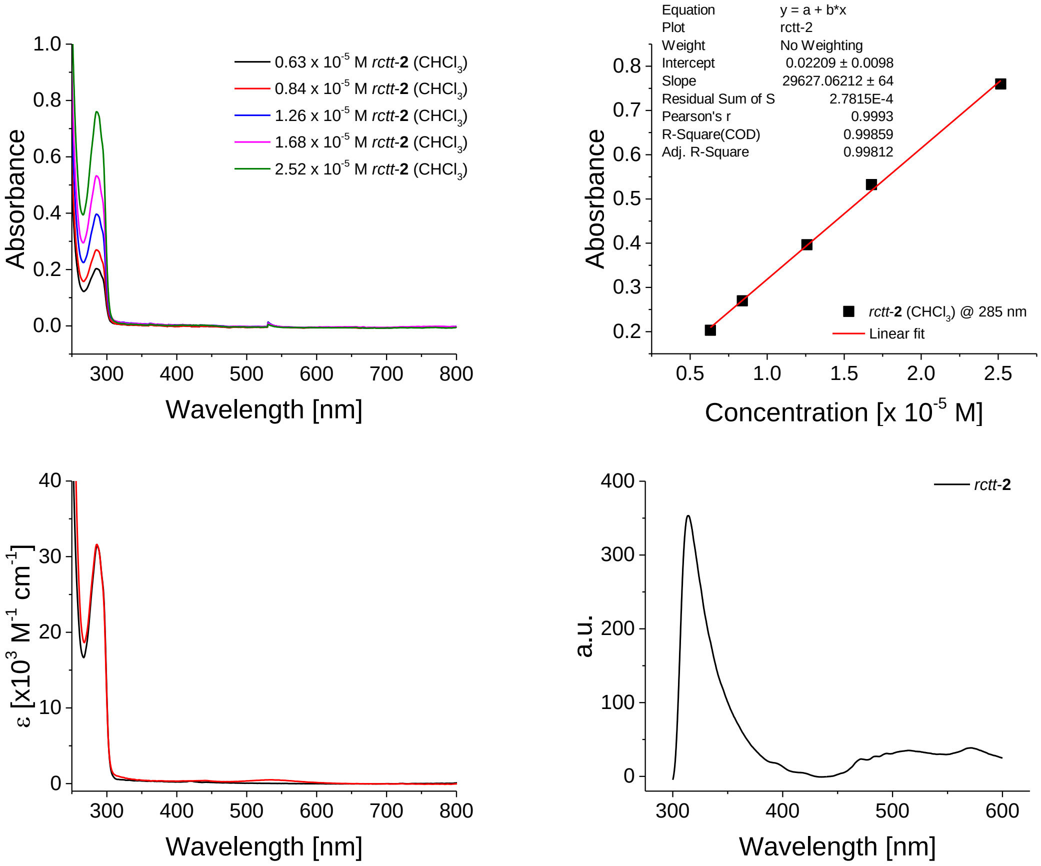

Supplementary Figure 20. (Top left) Electronic absorption spectra for rctt-**2** at various concentrations in $CHCl_3$. (Top right) Beer-Lambert plot of rctt-**2** with linear fit to determine the extinction coefficient at 285 nm. (Bottom left) Electronic absorption spectra for rctt-**2** at 2.47 × 10^{-5} mol in $CHCl_3$ (black) and at 2.40 × 10^{-5} mol dm^{-3} in $CHCl_3$ with TFA (0.36 mol dm^{-3}, red). (Bottom right) Emission spectra with excitation at 285 nm for rctt-**2** at 8.34 × 10^{-4} mol dm^{-3} in $CHCl_3$ with solid K_2CO_3 (68 mg).

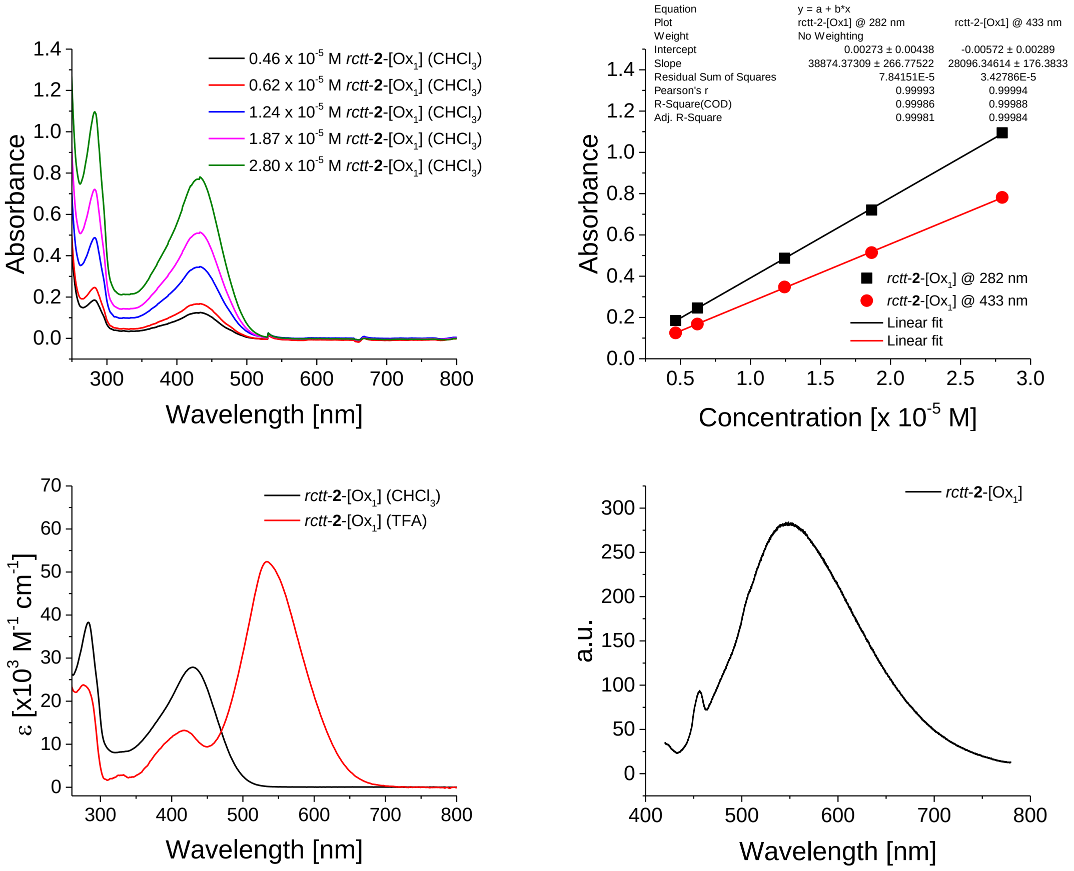

Supplementary Figure 21. (Top left) Electronic absorption spectra for rctt-**2**-[Ox_1] at various concentrations in $CHCl_3$. (Top right) Beer-Lambert plot of rctt-**2**-[Ox_1] with linear fit to determine the extinction coefficient at 282 nm and 433 nm. (Bottom left) Electronic absorption spectra for rctt-**2**-[Ox_1] at 2.47×10^{-5} mol dm^{-3} in $CHCl_3$ (black) and at 4.94×10^{-5} mol dm^{-3} in TFA (red). (Bottom right) Emission spectra with excitation at 400 nm for rctt-**2**-[Ox_1] at 1.24×10^{-5} mol dm^{-3} in $CHCl_3$.

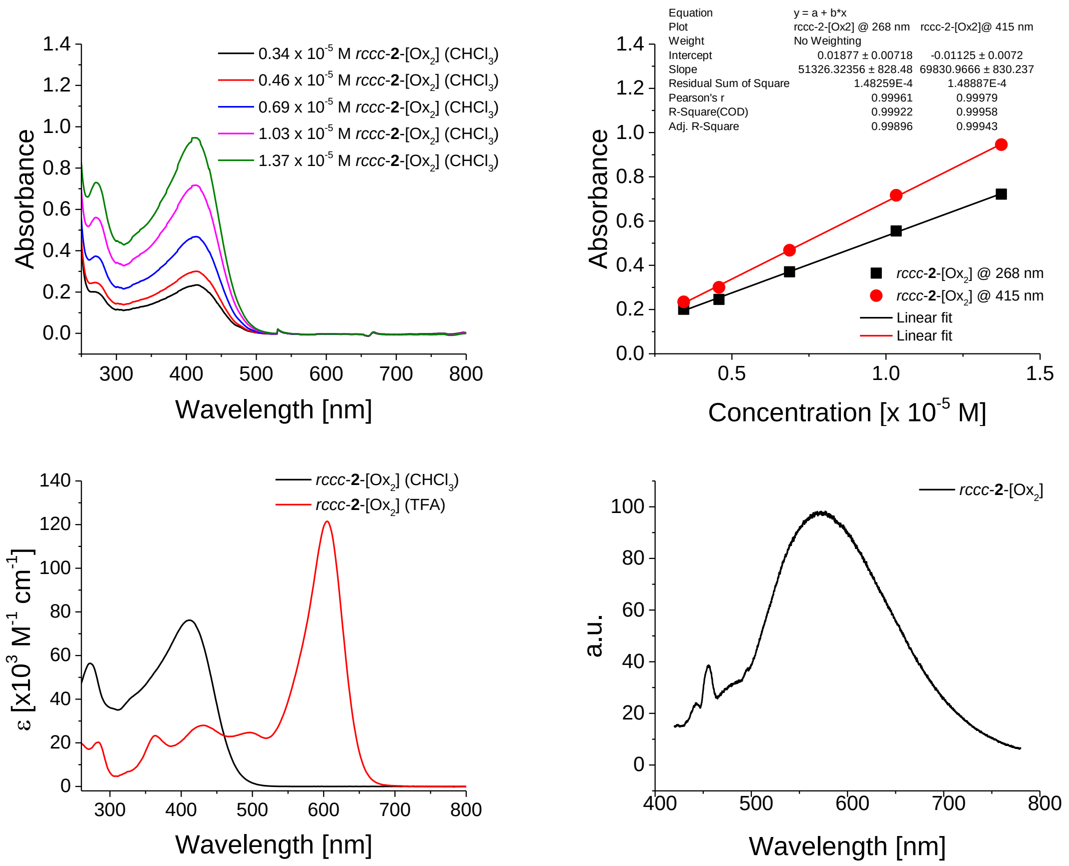

Supplementary Figure 22. (Top left) Electronic absorption spectra for rccc-**2**-[Ox$_2$] at various concentrations in $CHCl_3$. (Top right) Beer-Lambert plot of rccc-**2**-[Ox$_2$] with linear fit to determine the extinction coefficient at 268 nm and 415 nm. (Bottom left) Electronic absorption spectra for rccc-**2**-[Ox$_2$] at 2.47×10^{-5} mol dm^{-3} in $CHCl_3$ (black) and at 4.94×10^{-5} mol dm^{-3} in TFA (red). (Bottom right) Emission spectra with excitation at 400 nm for rccc-**2**-[Ox$_2$] at 1.03×10^{-5} mol dm^{-3} in $CHCl_3$.

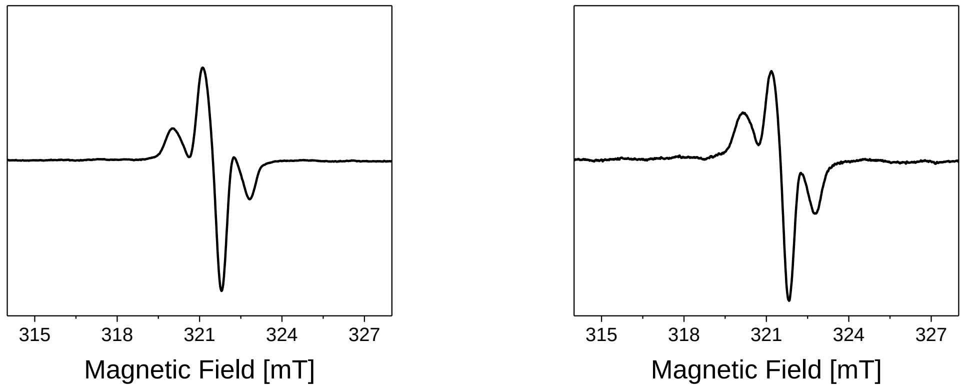

Supplementary Figure 23. X-Band ESR spectra of rctt-**2** recorded in solid-state at 298 K. Left: neutral; right: in acid. For both, microwave frequency: 8992 MHz; MW power 1.0 mW; sweep time: 60 s; time constant: 0.03 s; average of 10 scans.

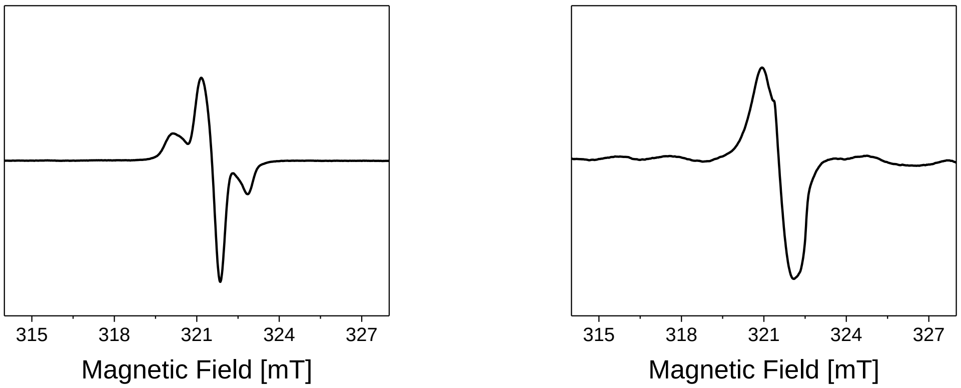

Supplementary Figure 24. X-Band ESR spectra of rctt-**2**-[Ox_1] recorded in solid-state at 298 K. Left: neutral (microwave frequency: 8993 MHz; MW power 1.0 mW; sweep time: 60 s; time constant: 0.03 s; average of 10 scans). Right: in acid (microwave frequency: 8987 MHz; MW power: 2.0 mW; sweep time: 60 s; time constant: 0.03 s; average of 30 scans).

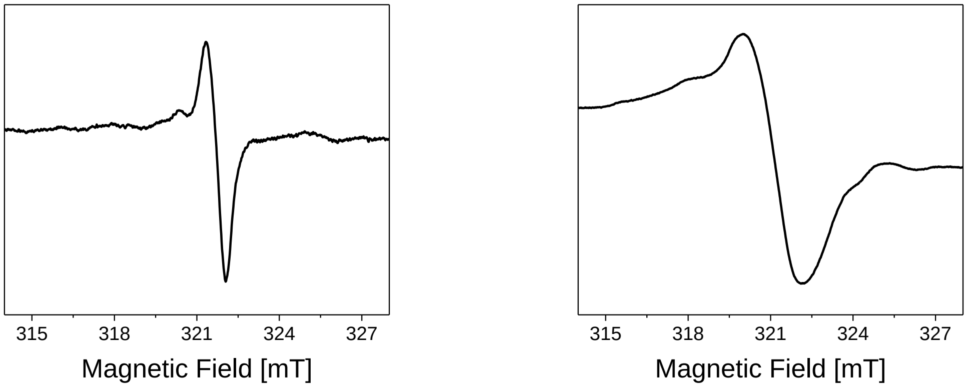

Supplementary Figure 25. X-Band ESR spectra of rccc-**2**-[Ox_2] recorded in solid-state at 298 K. Left: neutral (microwave frequency: 8995 MHz; MW power: 0.5 mW; sweep time: 60 s; time constant: 0.03 s; average of 50 scans). Right: in acid (microwave frequency: 8991 MHz; MW power: 2.0 mW; sweep time: 60 s; time constant: 0.03 s; average of 50 scans).

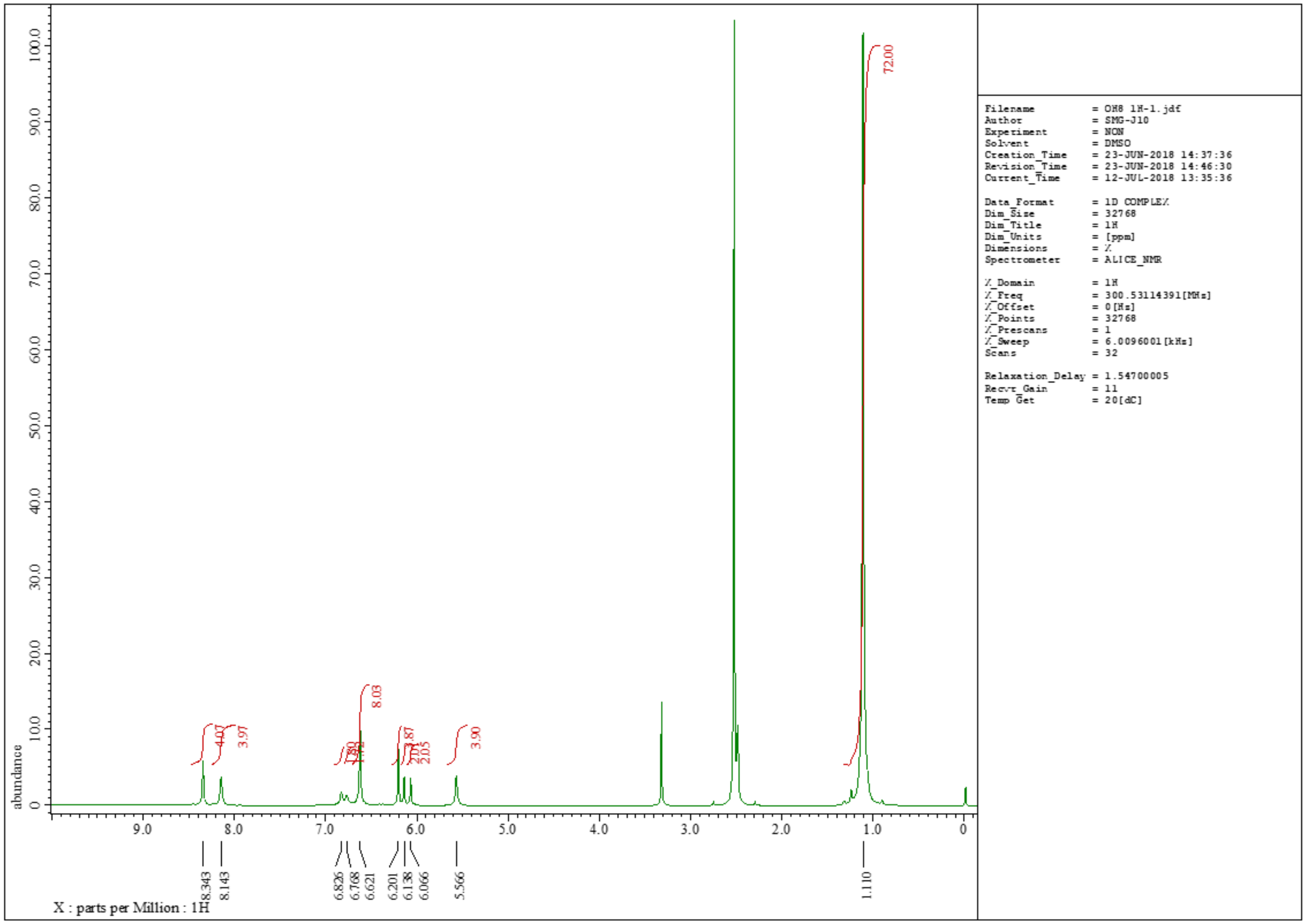

Supplementary Figure 26. ^{1}H NMR spectrum of rctt-**1**.

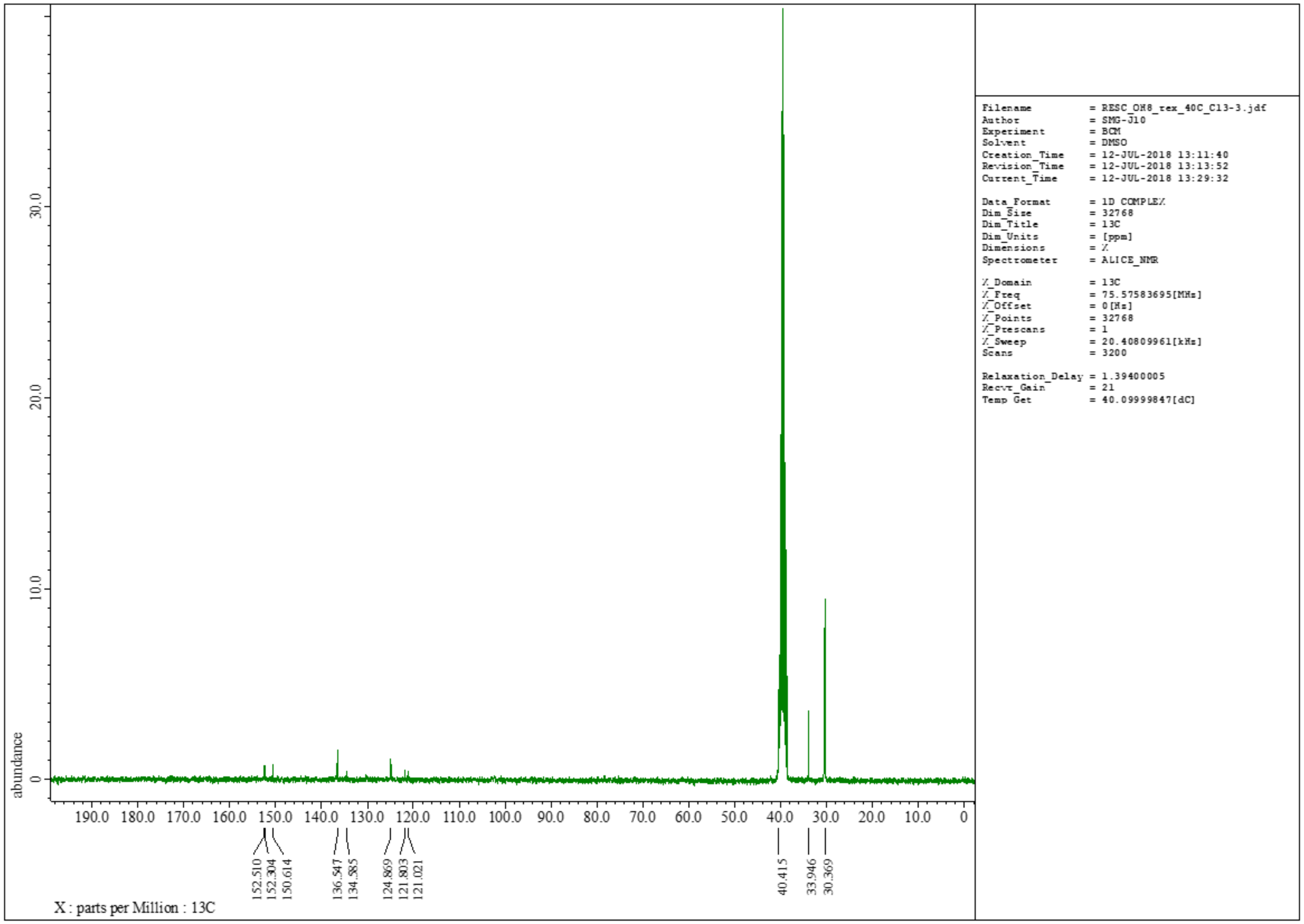

Supplementary Figure 27. ^{13}C NMR spectrum of rctt-**1**.

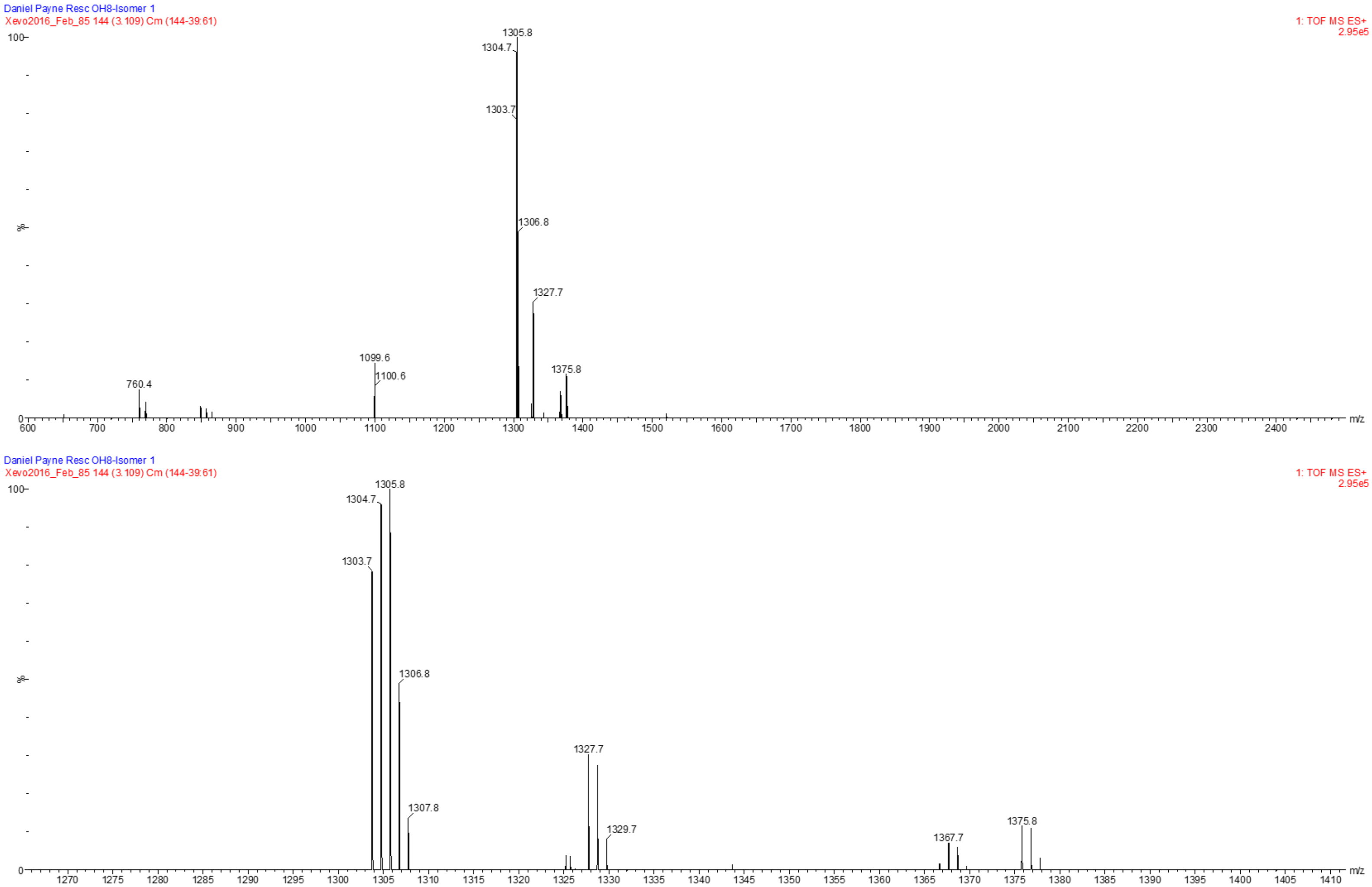

Supplementary Figure 28. HR-MS data for rctt-**1**.

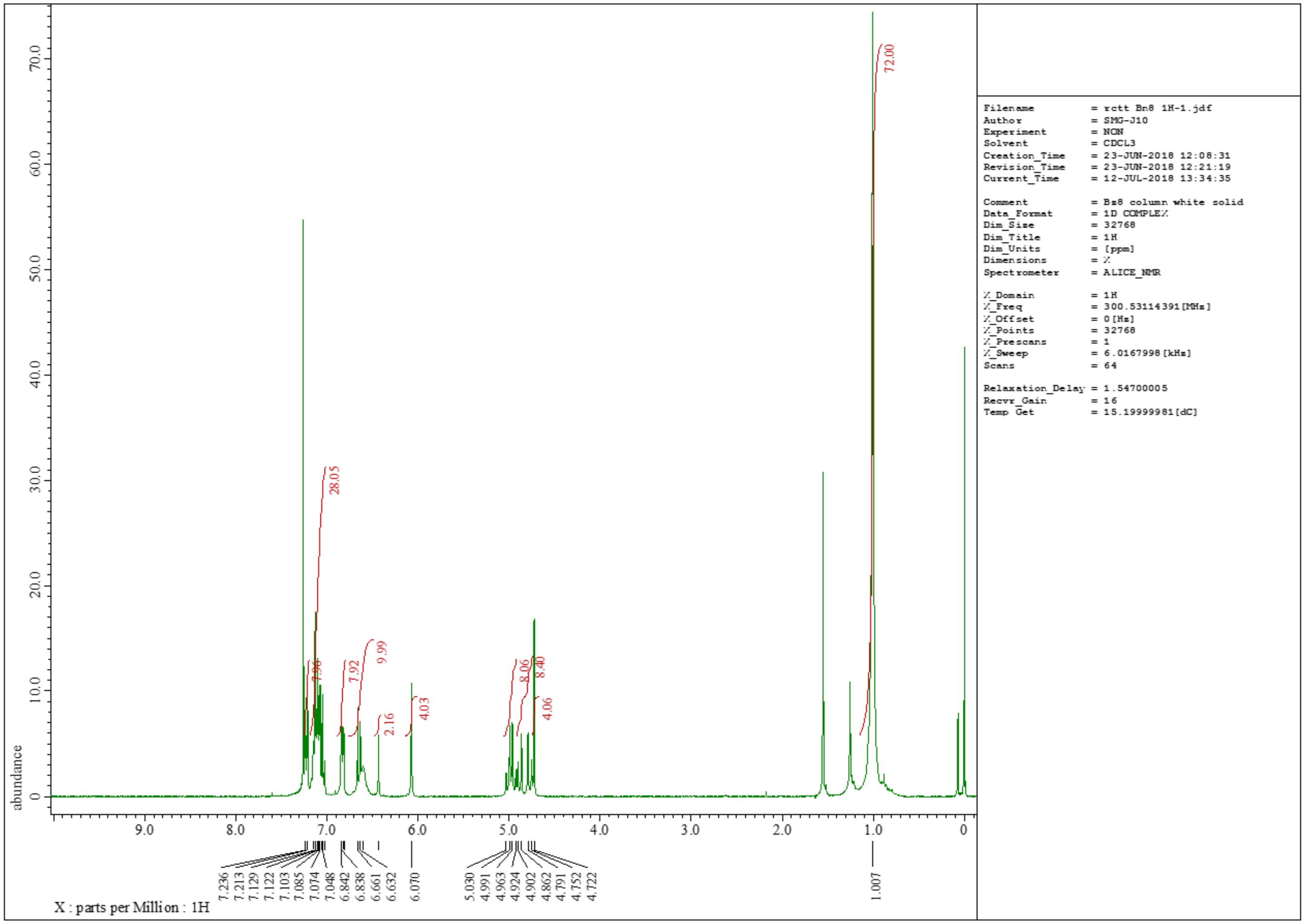

Supplementary Figure 29.^{1}H NMR spectrum of rctt-**2**.

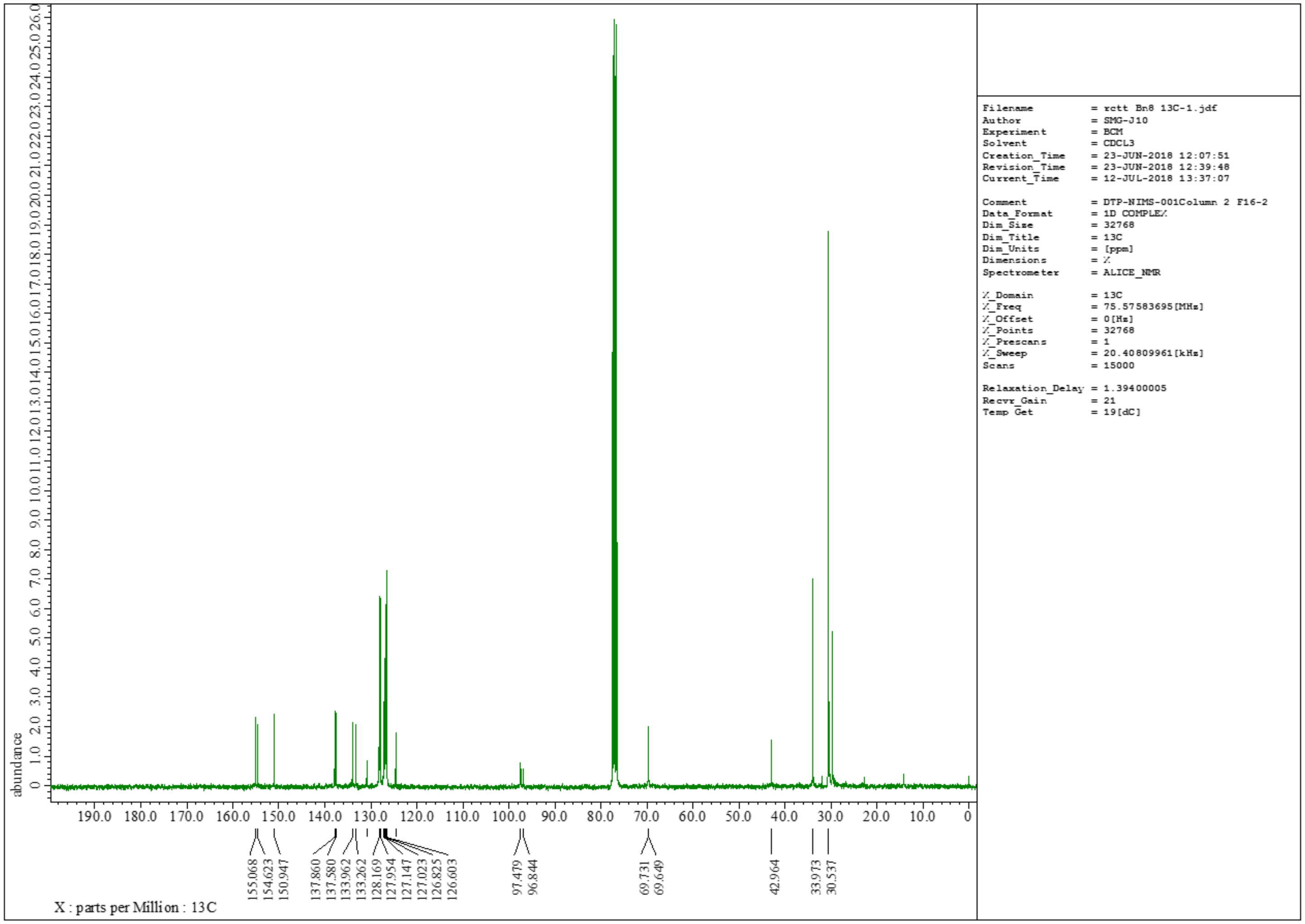

Supplementary Figure 30. ^{13}C NMR spectrum of rctt-**2**.

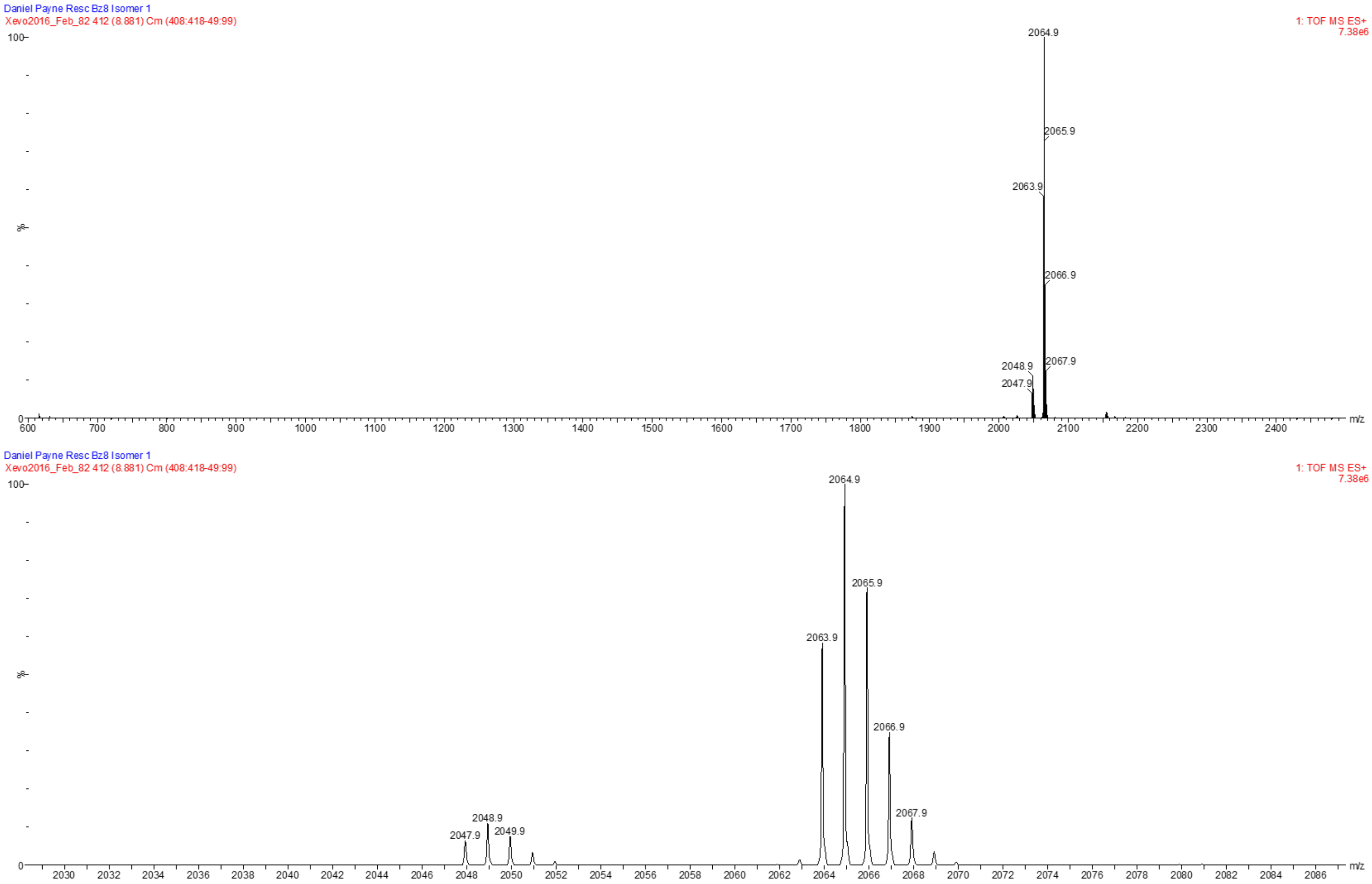

Supplementary Figure 31. HR-MS data for rctt-**2**.

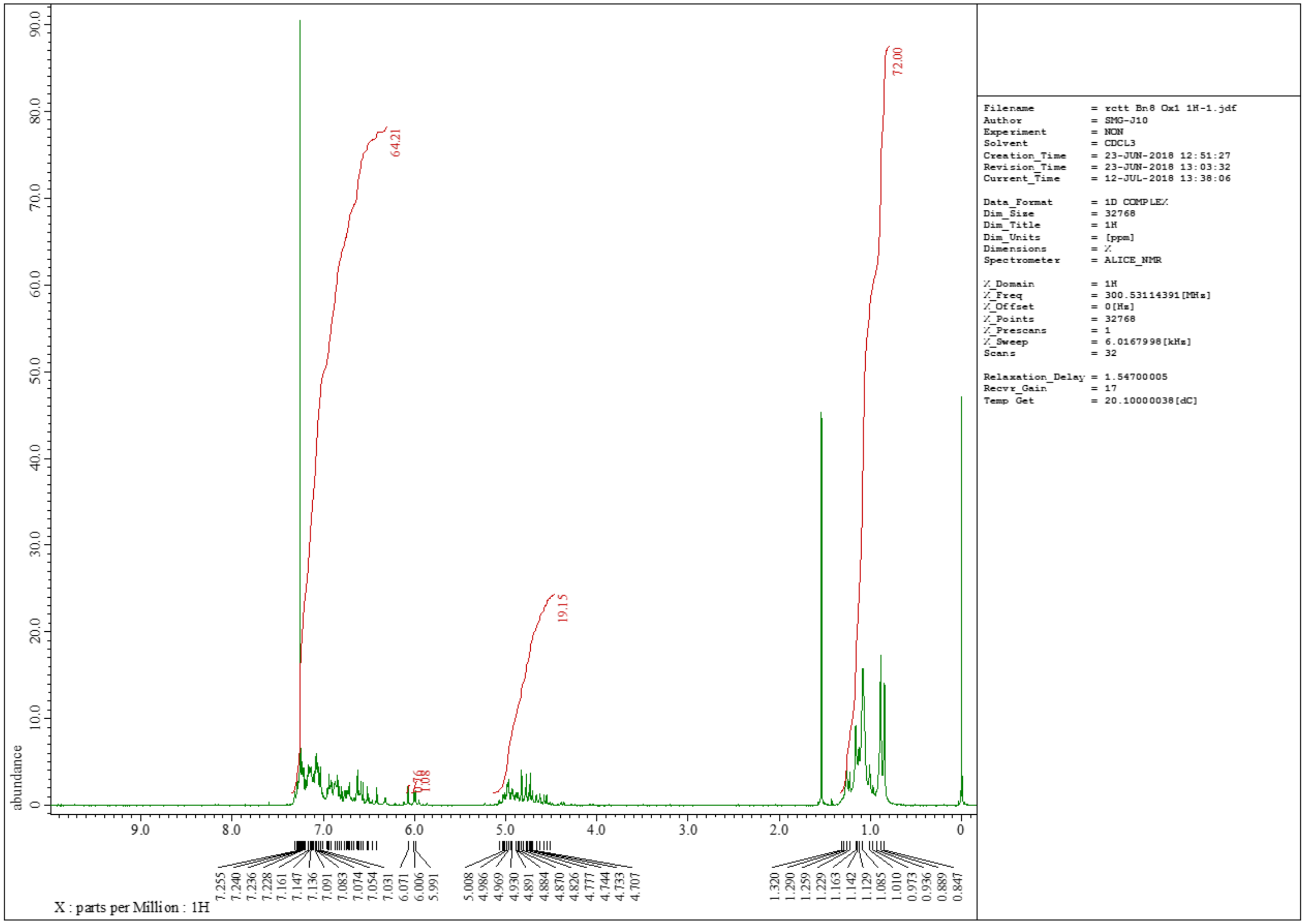

Supplementary Figure 32. ^{1}H NMR spectrum of rctt-**2**-[Ox_1].

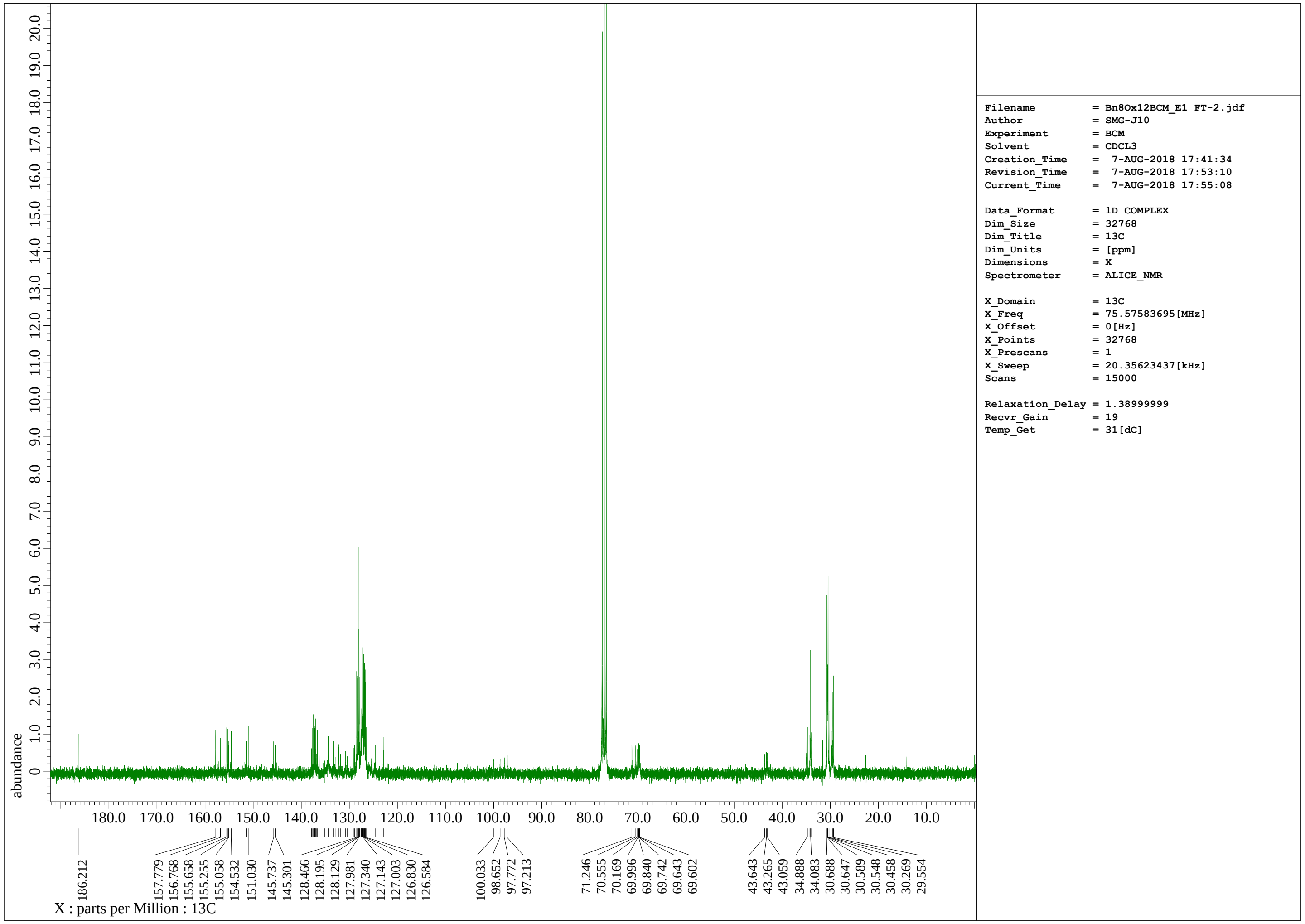

Supplementary Figure 33. ^{13}C NMR spectrum of rctt-**2**-[Ox$_1$]

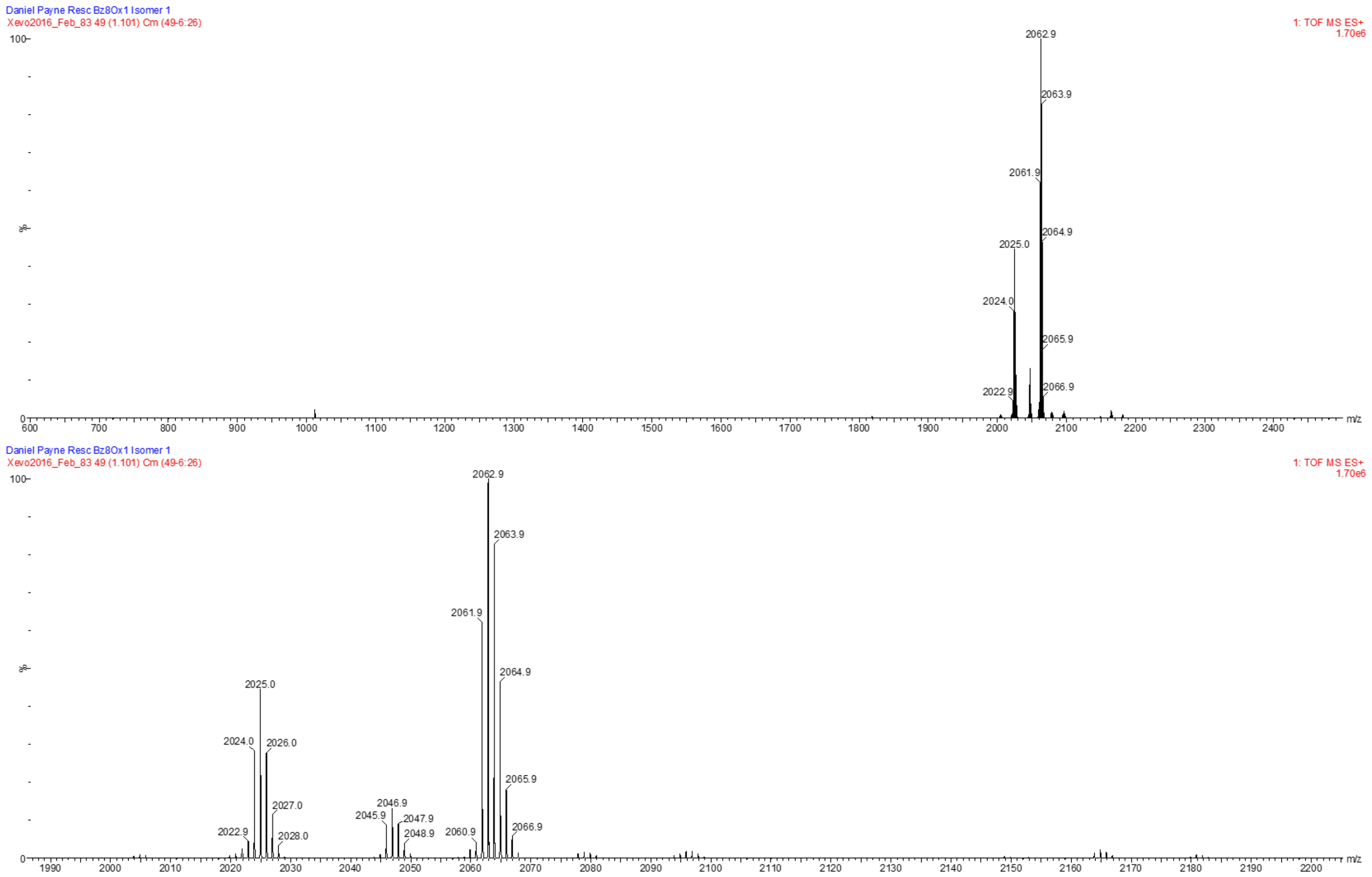

Supplementary Figure 34. HR-MS data for rctt-**2**-[Ox_1]

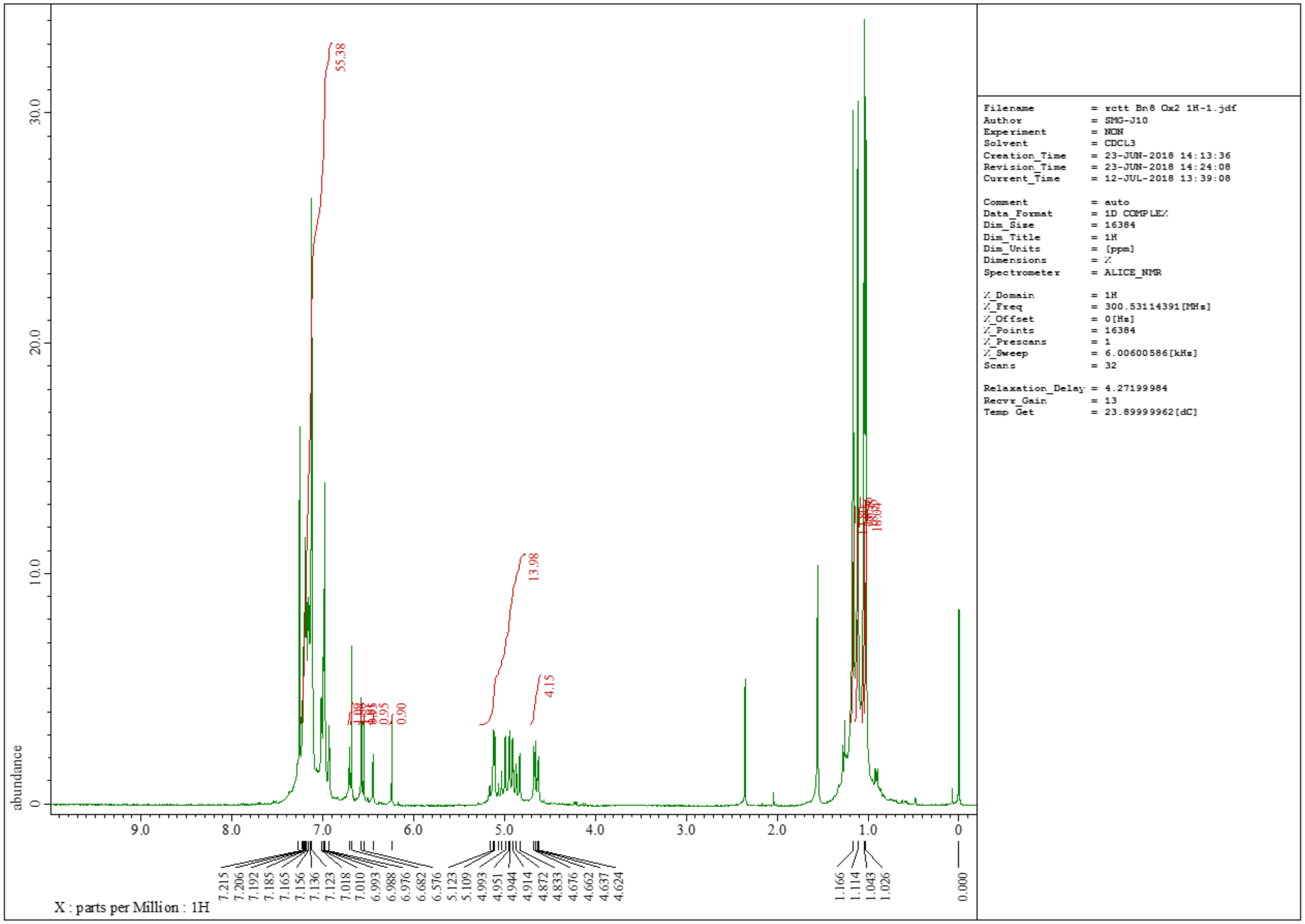

Supplementary Figure 35. ^{1}H NMR spectrum rccc-**2**-[Ox$_2$].

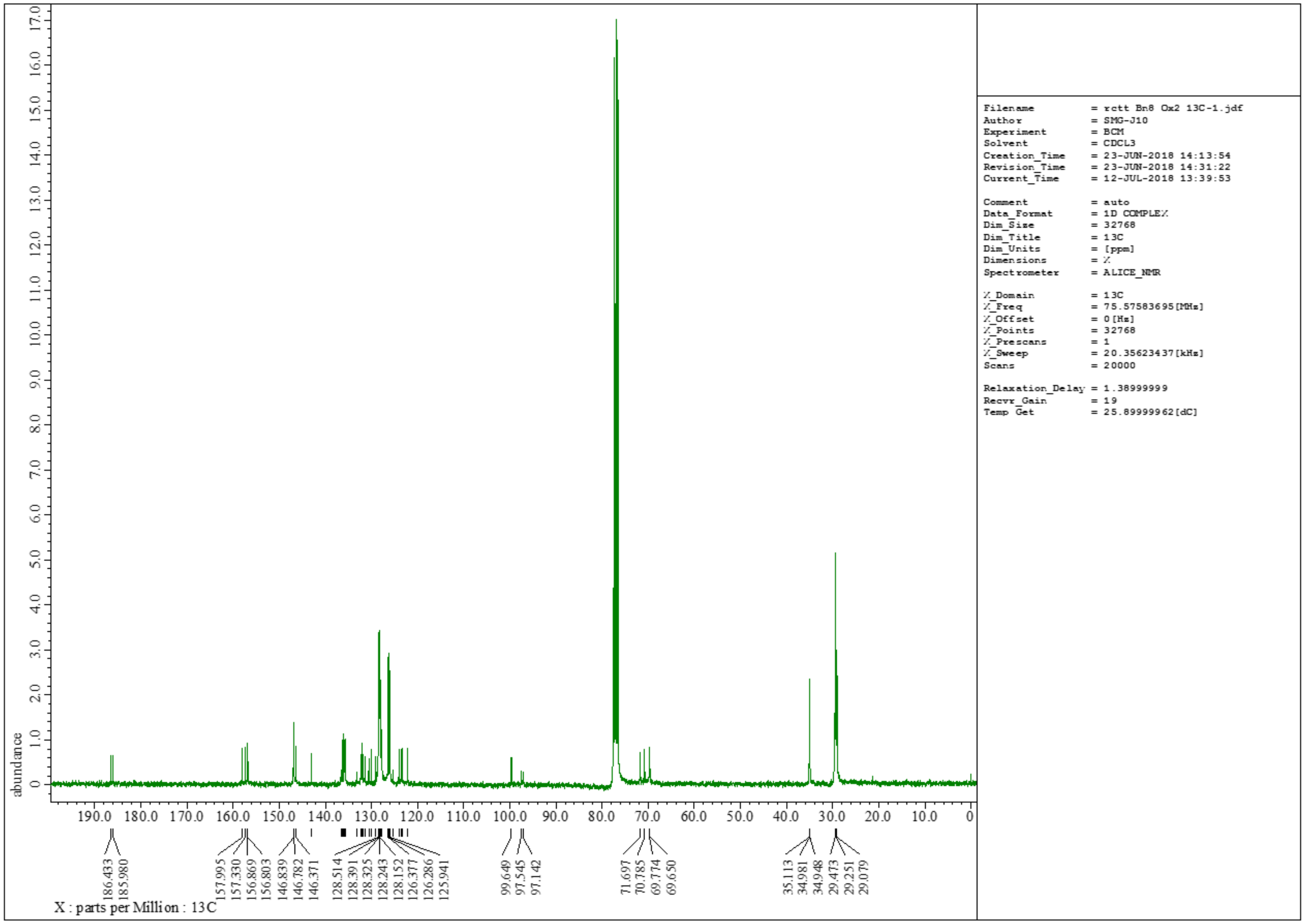

Supplementary Figure 36. ^{13}C NMR spectrum rccc-**2**-[Ox$_2$].

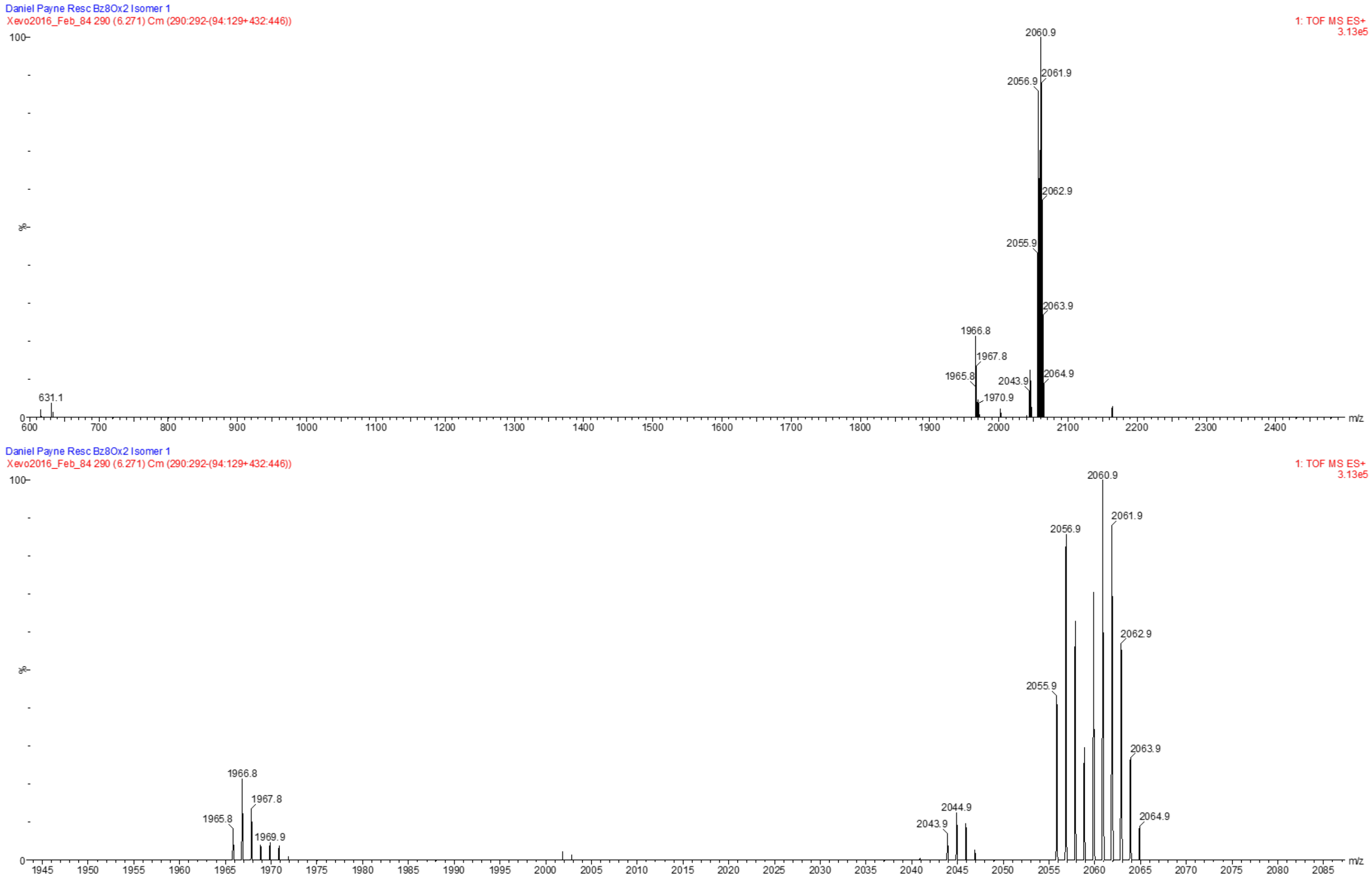

Supplementary Figure 37. HR-MS data for rccc-**2**-[Ox_2]

Supplementary Tables

Supplementary Table 1. Cartesian coordinates for the DFT calculated structure of rctt-**2**.

302

Energy = -6317.979741 H

C	3.289199	-3.634013	0.260748
C	0.716280	4.566836	0.649883
C	2.009898	4.852493	0.241250
C	2.771268	3.844950	-0.343372
C	2.271160	2.557236	-0.521441
C	0.967773	2.304719	-0.097984
C	0.178235	3.288173	0.479603
C	3.181439	1.478900	-1.084617
C	-1.276954	3.074912	0.857535
O	4.087087	4.123507	-0.705571
O	-0.121884	5.472325	1.269844
C	-2.179505	3.579428	-0.251817
C	-1.554518	1.630791	1.246653
C	2.430047	0.333127	-1.741909
C	1.999376	0.482526	-3.065119
C	1.393478	-0.570134	-3.740461
C	1.201554	-1.779384	-3.090386
C	1.551304	-1.946290	-1.748556
C	2.163849	-0.876738	-1.097209
C	-2.416489	0.828752	0.510959
C	-2.626914	-0.522993	0.785052
C	-1.987353	-1.069141	1.903669
C	-1.120470	-0.297937	2.656380
C	-0.882510	1.028811	2.320069
C	-3.315670	-1.356353	-0.224055
C	1.207917	-3.273076	-1.097503
C	-4.514381	-0.993991	-0.773481
C	-2.524451	-2.529093	-0.645502
C	1.898465	-3.547951	0.234412
C	-1.141251	-2.381765	-0.795179
C	-0.296387	-3.447849	-1.017141
C	-0.862507	-4.727358	-1.083717
C	-2.233346	-4.921011	-0.951073
C	-3.060163	-3.819296	-0.744051
O	2.156923	1.726820	-3.658419
O	0.603119	-2.843184	-3.770035
O	0.024063	-5.753429	-1.289283
O	-4.424684	-3.915777	-0.630212

O	-2.173717	-2.383693	2.301756
O	0.079934	1.678324	3.074784
C	-5.025763	-1.629211	-1.964510
C	-6.260310	-1.393118	-2.454731
C	-7.132636	-0.422451	-1.758972
C	-6.557627	0.364540	-0.641721
C	-5.328508	0.046605	-0.184966
C	-2.095227	3.044243	-1.529837
C	-2.983252	3.398589	-2.537756
C	-3.915210	4.414433	-2.247610
C	-3.969607	5.051672	-0.992208
C	-3.100952	4.583141	-0.003373
C	3.989787	-4.000852	1.405663
C	3.232836	-4.349813	2.539940
C	1.828493	-4.302680	2.550439
C	1.191351	-3.849198	1.390363
C	4.170138	1.012533	-0.024720
C	5.503172	0.804227	-0.360983
C	6.462415	0.446303	0.584736
C	6.039799	0.336672	1.926963
C	4.685943	0.456229	2.295492
C	3.782777	0.810786	1.288787
C	-7.381664	1.490158	-0.011679
O	-8.331828	-0.263585	-2.100678
C	-6.785542	-2.118244	-3.696892
C	1.002293	-4.779469	3.763329
O	3.960387	-4.775935	3.650217
C	5.527173	-4.049454	1.418595
C	-2.992449	2.634405	-3.873670
O	-4.776003	4.755211	-3.285631
C	-4.901065	6.247562	-0.699428
C	7.935844	0.290889	0.146349
O	7.056349	0.171976	2.857060
C	4.062251	0.299104	3.707105
C	-6.579766	2.250631	1.061891
C	-5.708932	-3.032440	-4.309651
C	-0.506775	-4.802120	3.453345
C	6.120694	-3.525968	0.097478
C	2.876732	-0.693226	3.615034
C	8.027716	-0.008167	-1.366926
C	-4.705779	6.779222	0.734911
C	-1.937081	1.509134	-3.909560
C	-7.209415	-1.091639	-4.770043
C	1.373738	-6.238139	4.137614
C	6.082194	-3.154465	2.547952

C	4.982740	-0.234850	4.826074
C	8.690823	-0.856791	0.852531
C	-2.705038	3.589734	-5.051197
C	-8.643442	0.903794	0.659867
C	-7.985432	-3.012534	-3.307506
C	1.191816	-3.813536	4.958436
C	6.004615	-5.505641	1.604058
C	3.544466	1.679390	4.167370
C	8.663463	1.622292	0.431439
C	-4.368406	1.946015	-4.037499
C	-7.779149	2.508245	-1.104363
C	-4.551701	7.422849	-1.646949
C	-6.394849	5.855783	-0.809003
C	1.464984	-3.524000	-4.752182
C	2.707341	1.799373	-5.013871
C	4.261856	4.898071	-1.926823
C	0.230784	6.872701	1.341520
C	-3.553963	-2.818626	2.570115
C	-0.295903	2.865284	3.844603
C	-0.281561	-7.104205	-0.847075
C	-5.102508	-5.116362	-0.199457
C	5.689065	5.357877	-1.999048
C	-0.945654	7.584699	1.946235
C	6.018362	6.462125	-2.785168
C	7.344685	6.858696	-2.923819
C	8.353244	6.158838	-2.265168
C	8.024739	5.066529	-1.465417
C	6.698571	4.664475	-1.333603
C	-1.571798	7.058100	3.078746
C	-2.642703	7.725718	3.662480
C	-3.094119	8.929894	3.124497
C	-2.477139	9.455879	1.993735
C	-1.410648	8.781494	1.402744
C	4.056952	2.455619	-4.963650
C	2.615652	-4.237694	-4.098455
C	4.257154	3.720947	-5.512910
C	5.503775	4.337951	-5.429243
C	6.556155	3.695395	-4.787328
C	6.363078	2.430760	-4.236064
C	5.122484	1.811484	-4.326234
C	3.870510	-3.631985	-4.022347
C	4.918777	-4.262888	-3.357304
C	4.714105	-5.499804	-2.749982
C	3.460937	-6.105772	-2.807240
C	2.419943	-5.478051	-3.482803

C	1.038465	-7.786126	-0.626832
C	-6.558770	-4.742412	-0.091833
C	1.942258	-7.242117	0.290049
C	3.169874	-7.851945	0.512444
C	3.515344	-9.004315	-0.193861
C	2.623598	-9.544375	-1.116543
C	1.386923	-8.935985	-1.331780
C	-7.550480	-5.698598	-0.307768
C	-8.895632	-5.349530	-0.213237
C	-9.258951	-4.038784	0.084239
C	-8.271452	-3.078360	0.289756
C	-6.929276	-3.429210	0.205352
C	-4.245423	-1.889641	3.526774
C	0.387683	2.788187	5.178245
C	0.337921	1.606719	5.920302
C	0.990743	1.519697	7.144031
C	1.693825	2.617606	7.640325
C	1.737075	3.800432	6.908508
C	1.087925	3.883352	5.678567
C	-3.701053	-1.663043	4.793420
C	-4.303570	-0.766096	5.667389
C	-5.462449	-0.090609	5.285609
C	-6.011234	-0.313759	4.026550
C	-5.398024	-1.203678	3.147378
H	3.826660	-3.407805	-0.649664
H	2.454714	5.827442	0.383721
H	0.558444	1.309444	-0.224710
H	3.766366	1.955561	-1.875908
H	-1.477170	3.714299	1.719350
H	1.042747	-0.451444	-4.757429
H	2.483271	-1.003815	-0.070427
H	-2.917260	1.241226	-0.353941
H	-0.622009	-0.727579	3.513246
H	1.570085	-4.048870	-1.775112
H	-0.712402	-1.392314	-0.703122
H	-2.655953	-5.912880	-1.028660
H	-4.362470	-2.314214	-2.466425
H	-4.925731	0.553219	0.677907
H	-1.334197	2.301784	-1.723133
H	-3.112006	5.028157	0.980319
H	0.114976	-3.773847	1.372413
H	5.791943	0.956150	-1.391589
H	2.745447	0.977553	1.551803
H	3.438284	-4.756138	4.464593
H	-5.385582	5.466472	-3.046377

H	6.744942	-0.194294	3.694753
H	-6.303143	1.605986	1.902517
H	-5.669589	2.697764	0.647483
H	-7.201445	3.058766	1.460488
H	-4.830947	-2.465244	-4.635890
H	-5.386983	-3.800363	-3.598709
H	-6.127227	-3.536679	-5.186121
H	-0.726933	-5.469432	2.613205
H	-1.039778	-5.181566	4.331665
H	-0.901038	-3.812587	3.216359
H	5.849669	-2.479160	-0.077081
H	7.211846	-3.588716	0.149824
H	5.795966	-4.122773	-0.756565
H	3.212316	-1.667758	3.246112
H	2.435821	-0.826453	4.609048
H	2.087013	-0.327111	2.957231
H	7.448760	-0.900459	-1.626483
H	7.677312	0.824321	-1.979611
H	9.073784	-0.184810	-1.634057
H	-3.680056	7.112455	0.909718
H	-4.963787	6.024755	1.483922
H	-5.363948	7.639776	0.887627
H	-2.131256	0.746242	-3.147655
H	-1.989106	1.013099	-4.883746
H	-0.919644	1.889194	-3.779312
H	-7.564962	-1.615224	-5.664257
H	-6.357208	-0.465268	-5.054912
H	-8.008950	-0.450832	-4.398127
H	0.765599	-6.562911	4.987531
H	2.422310	-6.384088	4.398283
H	1.159922	-6.897096	3.290534
H	7.175980	-3.141295	2.507708
H	5.767014	-3.513171	3.525965
H	5.726183	-2.131602	2.406696
H	4.381806	-0.360928	5.731177
H	5.776109	0.472960	5.088568
H	5.395106	-1.223647	4.590053
H	9.654741	-1.004100	0.353131
H	8.125341	-1.788397	0.785916
H	8.862475	-0.639586	1.902204
H	-2.716511	3.028521	-5.991991
H	-1.715060	4.044137	-4.936377
H	-3.451944	4.381141	-5.102726
H	-9.241177	1.708782	1.101350
H	-8.357406	0.211899	1.460471

H	-9.253063	0.369422	-0.068635
H	-8.788344	-2.415034	-2.878711
H	-8.361053	-3.528282	-4.198726
H	-7.677864	-3.764817	-2.574262
H	2.232247	-3.687574	5.275732
H	0.824756	-2.820245	4.685038
H	0.624309	-4.169264	5.824179
H	5.609001	-5.932086	2.527163
H	5.677702	-6.118154	0.757003
H	7.099213	-5.535996	1.645082
H	3.108016	1.602392	5.167674
H	4.363412	2.405377	4.198038
H	2.767895	2.054835	3.498444
H	8.593551	1.877441	1.491962
H	9.722053	1.544698	0.158213
H	8.214076	2.427761	-0.154791
H	-5.176768	2.676895	-4.053230
H	-4.389044	1.374986	-4.972757
H	-4.537747	1.247445	-3.205380
H	-8.380958	2.031688	-1.877287
H	-8.360137	3.320076	-0.654160
H	-6.884021	2.942026	-1.563853
H	-3.526834	7.752562	-1.455069
H	-4.617257	7.169809	-2.708359
H	-5.225460	8.264770	-1.459781
H	-7.020033	6.731665	-0.610328
H	-6.627947	5.088668	-0.066026
H	-6.695650	5.451027	-1.778239
H	0.788991	-4.217589	-5.249678
H	1.824336	-2.787365	-5.477907
H	2.777186	0.790348	-5.429991
H	2.004058	2.379999	-5.611380
H	4.012463	4.262787	-2.785672
H	3.577092	5.753522	-1.922957
H	0.457357	7.254640	0.341022
H	1.123449	6.988937	1.968190
H	-3.413721	-3.814749	2.990488
H	-4.107256	-2.903271	1.632776
H	-1.385186	2.873531	3.964221
H	0.008659	3.760704	3.300321
H	-0.860532	-7.048009	0.081708
H	-0.879538	-7.616735	-1.605925
H	-4.682962	-5.434887	0.763009
H	-4.965904	-5.925136	-0.923893
H	5.234863	7.008671	-3.299348

H	7.590157	7.715948	-3.539054
H	9.386257	6.468731	-2.366534
H	8.804924	4.527886	-0.940657
H	6.431968	3.816384	-0.715677
H	-1.218622	6.121668	3.493940
H	-3.124338	7.309146	4.538552
H	-3.927247	9.450015	3.580645
H	-2.830194	10.385152	1.564226
H	-0.938750	9.189478	0.515921
H	3.433365	4.228716	-6.002096
H	5.647946	5.325068	-5.850024
H	7.514745	4.188207	-4.688643
H	7.183179	1.935865	-3.732196
H	4.974207	0.824230	-3.899988
H	4.024951	-2.661524	-4.481374
H	5.889818	-3.785520	-3.305448
H	5.526389	-5.988486	-2.225241
H	3.292777	-7.056686	-2.318576
H	1.437634	-5.933879	-3.497654
H	1.687517	-6.330864	0.816115
H	3.854115	-7.423991	1.233776
H	4.474905	-9.477678	-0.025460
H	2.888341	-10.436635	-1.670411
H	0.695877	-9.357362	-2.053503
H	-7.274337	-6.717007	-0.559051
H	-9.658515	-6.098472	-0.386848
H	-10.304938	-3.764546	0.142305
H	-8.544634	-2.050501	0.495946
H	-6.160893	-2.677098	0.315180
H	-0.199528	0.753323	5.523735
H	0.954771	0.597782	7.711658
H	2.205407	2.549165	8.592479
H	2.286366	4.653643	7.286782
H	1.143497	4.796959	5.096192
H	-2.795890	-2.184193	5.084236
H	-3.872803	-0.592740	6.645864
H	-5.933693	0.605844	5.967999
H	-6.912908	0.205380	3.725351
H	-5.818472	-1.360299	2.160929

Supplementary Table 2. Cartesian coordinates for the DFT calculated structure of rccc-**2**

302
ox1-rccc-wb97xd-431g-s.log (E=-6317.98177738, step=9)

C	-0.132017	-4.506205	-1.851311
C	3.004080	3.216491	-0.423658
C	4.326232	2.931979	-0.117213
C	4.692944	1.637795	0.234710
C	3.765018	0.596326	0.240529
C	2.449896	0.909523	-0.065189
C	2.040542	2.198970	-0.390904
C	4.173875	-0.839654	0.526551
C	0.601073	2.468293	-0.532140
O	6.008006	1.406247	0.638293
O	2.549996	4.482154	-0.702351
C	0.065506	3.139951	-1.598703
C	-0.252497	1.865978	0.507069
C	3.043505	-1.579229	1.226728
C	2.756566	-1.238911	2.550150
C	1.722053	-1.850474	3.241647
C	0.968660	-2.836217	2.620782
C	1.186461	-3.169197	1.279124
C	2.229283	-2.530621	0.608928
C	-1.339673	1.091457	0.098812
C	-2.168304	0.421551	0.984123
C	-1.883632	0.558500	2.345125
C	-0.797474	1.301756	2.788299
C	0.023832	1.951240	1.877335
C	-3.295913	-0.490748	0.535102
C	0.252597	-4.192725	0.647339
C	-3.835335	-0.164962	-0.850644
C	-2.876355	-1.951115	0.627721
C	0.680205	-4.658826	-0.739538
C	-1.554393	-2.378378	0.575442
C	-1.193981	-3.722086	0.678151
C	-2.216356	-4.658996	0.822369
C	-3.552727	-4.267471	0.855852
C	-3.875805	-2.920991	0.761235
O	3.511000	-0.231943	3.145184
O	0.001597	-3.566704	3.296655
O	-1.839425	-5.992533	0.893459
O	-5.171702	-2.446415	0.798382
O	-2.702099	-0.088504	3.270485
O	1.108921	2.700955	2.304478
C	-3.060469	-0.358170	-1.991820

C	-3.490002	0.033607	-3.257011
C	-4.808772	0.509721	-3.367065
C	-5.652482	0.655840	-2.250457
C	-5.116551	0.346956	-0.996789
C	0.839760	3.378686	-2.791932
C	0.358100	4.041846	-3.865692
C	-1.016655	4.593972	-3.810778
C	-1.833264	4.334875	-2.602780
C	-1.290536	3.636763	-1.581370
C	0.206871	-5.029912	-3.102036
C	1.454630	-5.667884	-3.217429
C	2.311688	-5.846633	-2.112762
C	1.885459	-5.341169	-0.888865
C	4.671805	-1.489013	-0.756802
C	5.874589	-2.175046	-0.780281
C	6.424385	-2.705801	-1.952845
C	5.710358	-2.493191	-3.142471
C	4.451127	-1.849565	-3.153782
C	3.967208	-1.356843	-1.948591
C	-7.124662	1.095490	-2.388033
O	-5.240446	0.805218	-4.657244
C	-2.537310	-0.012924	-4.463197
C	3.646162	-6.605009	-2.228420
O	1.903271	-6.189798	-4.429340
C	-0.806823	-4.957918	-4.265685
C	1.192326	4.225915	-5.138560
O	-1.476468	5.270192	-4.766678
C	-3.256205	4.897755	-2.520375
C	7.793785	-3.413190	-1.817463
O	6.298169	-2.929454	-4.320248
C	3.635173	-1.653689	-4.447267
C	-7.897111	0.079840	-3.265570
C	-1.113533	-0.427265	-4.045023
C	3.381933	-8.061727	-2.670074
C	-1.122062	-6.381626	-4.790809
C	4.381963	-0.711410	-5.424853
C	7.714831	-4.460742	-0.680535
C	-3.947637	4.494060	-1.204902
C	0.478198	3.544577	-6.327037
C	-3.044563	-1.014565	-5.522149
C	4.584580	-5.904298	-3.232509
C	-0.294938	-4.045431	-5.406419
C	3.301761	-3.019064	-5.098466
C	8.851750	-2.344253	-1.459102
C	1.394856	5.730169	-5.433127

C	-7.237976	2.536142	-2.945709
C	-2.405999	1.396999	-5.082058
C	4.387879	-6.653955	-0.878293
C	-2.158540	-4.375256	-3.801710
C	2.272041	-0.990295	-4.167148
C	8.291449	-4.172426	-3.063776
C	2.587148	3.587244	-4.999484
C	-7.833317	1.119276	-1.022165
C	-3.201895	6.441649	-2.574952
C	-4.114601	4.353447	-3.682996
C	-3.509756	0.804654	4.125020
C	0.808657	3.794035	3.229928
C	-1.001650	-2.853932	4.074833
C	4.021555	-0.441816	4.499826
C	3.948505	0.862453	5.236897
C	-0.473110	-2.382822	5.407884
C	-0.184973	4.764587	2.649664
C	-4.475827	1.627532	3.325884
C	-4.091231	2.870724	2.819590
C	-4.974912	3.623800	2.054327
C	-6.250375	3.136457	1.783427
C	-6.638114	1.890999	2.275288
C	-5.752323	1.139970	3.039987
C	-1.312564	5.145695	3.376598
C	-2.246003	6.022243	2.825242
C	-2.058961	6.515052	1.536404
C	-0.931754	6.138094	0.807463
C	0.002865	5.271061	1.361608
C	0.444812	-3.153899	6.124021
C	0.964069	-2.691679	7.328862
C	0.567301	-1.454431	7.835073
C	-0.353310	-0.684651	7.129471
C	-0.867906	-1.147405	5.920534
C	5.098322	1.466301	5.740588
C	5.020534	2.687128	6.406682
C	3.789239	3.311790	6.570330
C	2.634208	2.711373	6.069118
C	2.713087	1.494376	5.402881
C	-6.271418	-3.389714	0.762303
C	-2.611951	-6.895637	1.740180
C	6.211109	1.628279	2.093425
C	3.379359	5.643238	-0.452931
C	6.018951	3.070423	2.466296
C	-7.548305	-2.600512	0.745073
C	-2.541010	-6.503516	3.191935

C	-7.884987	-1.803784	1.841537
C	-9.076296	-1.086488	1.852958
C	-9.946519	-1.161989	0.765954
C	-9.614994	-1.949740	-0.331913
C	-8.416964	-2.661908	-0.343809
C	-1.317721	-6.565890	3.863259
C	-1.228388	-6.201419	5.201030
C	-2.359761	-5.752271	5.880463
C	-3.580749	-5.676941	5.216235
C	-3.669287	-6.053436	3.877454
C	7.003119	4.011879	2.155264
C	6.794966	5.363232	2.409976
C	5.599225	5.784623	2.990455
C	4.616179	4.850542	3.307853
C	4.818171	3.500132	3.035186
C	2.503056	6.842302	-0.690074
C	1.558429	6.821505	-1.718630
C	0.750246	7.928858	-1.953097
C	0.877574	9.067267	-1.158476
C	1.815128	9.089877	-0.129429
C	2.623525	7.979711	0.106028
H	-1.074865	-3.999197	-1.724943
H	5.079696	3.704457	-0.093350
H	1.699531	0.130414	-0.011843
H	5.017783	-0.808238	1.219957
H	1.506800	-1.567654	4.259567
H	2.423998	-2.791300	-0.422981
H	-1.532311	1.024524	-0.960129
H	-0.587260	1.361316	3.848084
H	-4.117846	-0.354662	1.241223
H	-0.767340	-1.639342	0.486226
H	-4.328695	-5.010695	0.952014
H	-2.085338	-0.810994	-1.878949
H	-5.715597	0.471080	-0.106632
H	1.834228	2.959285	-2.810037
H	-1.852585	3.479077	-0.673900
H	2.503230	-5.471356	-0.011743
H	6.423496	-2.271694	0.148156
H	3.030257	-0.821920	-1.931064
H	-6.072881	1.297602	-4.667716
H	5.734544	-2.776973	-5.090364
H	-7.899272	-0.896076	-2.771579
H	-8.935212	0.406672	-3.384956
H	-7.464969	-0.054559	-4.259134
H	-0.682314	0.278178	-3.324929

H	-1.070603	-1.432638	-3.616817
H	-0.478755	-0.421433	-4.934922
H	2.895295	-8.090995	-3.644896
H	4.330819	-8.606192	-2.733205
H	2.742940	-8.570329	-1.940414
H	-0.243357	-6.923534	-5.142908
H	-1.571589	-6.976202	-3.990316
H	-1.836945	-6.319222	-5.617315
H	5.380119	-1.057127	-5.705506
H	3.799254	-0.591775	-6.343630
H	4.503358	0.272545	-4.962593
H	7.453074	-4.021650	0.284584
H	6.970680	-5.224616	-0.922765
H	8.687649	-4.950746	-0.568342
H	-4.034371	3.406538	-1.104205
H	-4.959863	4.912037	-1.192772
H	-3.414123	4.886379	-0.333282
H	-0.516460	3.965671	-6.474669
H	1.062749	3.687307	-7.242507
H	0.383275	2.469281	-6.143200
H	-2.343859	-1.051731	-6.364050
H	-4.025928	-0.718780	-5.893260
H	-3.117344	-2.018706	-5.094850
H	5.539349	-6.440685	-3.279133
H	4.786311	-4.880706	-2.908186
H	4.145545	-5.879391	-4.228604
H	-1.055942	-3.967052	-6.189112
H	0.622954	-4.400420	-5.886179
H	-0.088604	-3.042139	-5.025165
H	2.702338	-3.612129	-4.403797
H	4.169533	-3.629281	-5.357999
H	2.722130	-2.858615	-6.013009
H	9.832586	-2.815295	-1.330864
H	8.597300	-1.822586	-0.531909
H	8.926729	-1.600948	-2.258661
H	1.990001	5.848453	-6.345235
H	1.933365	6.213488	-4.611159
H	0.435657	6.227897	-5.568687
H	-8.291776	2.826589	-2.999870
H	-6.813136	2.685718	-3.942620
H	-6.718250	3.229643	-2.278843
H	-3.367136	1.771739	-5.428564
H	-2.003814	2.092216	-4.340515
H	-1.713132	1.362779	-5.929508
H	5.332329	-7.189756	-1.015710

H	4.622819	-5.648973	-0.512835
H	3.813438	-7.184659	-0.112516
H	-2.855862	-4.386000	-4.644620
H	-2.593593	-4.970914	-2.994234
H	-2.072180	-3.341445	-3.459201
H	1.731463	-0.898075	-5.113145
H	1.661399	-1.594542	-3.488913
H	2.376790	0.014890	-3.747928
H	8.494863	-3.502281	-3.896371
H	9.218149	-4.693022	-2.796264
H	7.561906	-4.912411	-3.397429
H	3.146477	3.751414	-5.925444
H	3.157275	4.035048	-4.178630
H	2.525685	2.506165	-4.835026
H	-7.386853	1.865334	-0.362683
H	-8.886232	1.379005	-1.168701
H	-7.796309	0.146069	-0.529331
H	-2.609412	6.830016	-1.739286
H	-2.756612	6.778049	-3.511431
H	-4.214815	6.851972	-2.496950
H	-4.215103	3.267265	-3.601330
H	-3.665521	4.596079	-4.645295
H	-5.116306	4.795040	-3.636023
H	-4.016549	0.120955	4.803910
H	-2.833996	1.445952	4.699861
H	1.782780	4.248411	3.401814
H	0.444902	3.390162	4.179311
H	-1.799666	-3.586880	4.203266
H	-1.391260	-2.015829	3.492180
H	3.428520	-1.209512	5.003998
H	5.052187	-0.799547	4.422928
H	-3.096627	3.252323	3.018042
H	-4.664216	4.588313	1.673703
H	-6.940252	3.724571	1.190133
H	-7.624190	1.500251	2.056864
H	-6.051162	0.170582	3.421018
H	-1.467352	4.752490	4.376429
H	-3.119123	6.310492	3.398086
H	-2.785489	7.190841	1.101077
H	-0.772653	6.518336	-0.193116
H	0.867572	4.974244	0.780179
H	0.774975	-4.097001	5.707881
H	1.684720	-3.292336	7.870796
H	0.978572	-1.091426	8.768961
H	-0.661163	0.280764	7.513455

H	-1.563740	-0.545199	5.350677
H	6.061106	0.987439	5.599857
H	5.921583	3.154161	6.783982
H	3.726724	4.263356	7.083789
H	1.673825	3.194002	6.204686
H	1.820472	1.031314	4.998156
H	-6.226229	-4.030198	1.651315
H	-6.185997	-4.021372	-0.127771
H	-2.141164	-7.862681	1.565888
H	-3.647996	-6.955902	1.394719
H	7.233441	1.292489	2.262199
H	5.508196	0.990712	2.633992
H	3.759669	5.617104	0.574138
H	4.232338	5.632286	-1.141608
H	-7.212765	-1.760274	2.689957
H	-9.332580	-0.478832	2.712631
H	-10.876203	-0.606651	0.775407
H	-10.282446	-2.005399	-1.182841
H	-8.158773	-3.270674	-1.203003
H	-0.433905	-6.894109	3.329402
H	-0.277255	-6.261851	5.714366
H	-2.286156	-5.457631	6.920015
H	-4.462714	-5.325369	5.737371
H	-4.622922	-5.996239	3.364200
H	7.930626	3.682066	1.700101
H	7.562481	6.086176	2.161980
H	5.438360	6.835546	3.199899
H	3.694805	5.177203	3.773672
H	4.041674	2.775619	3.252547
H	1.440286	5.924216	-2.311035
H	0.018162	7.896454	-2.751589
H	0.246158	9.929029	-1.337222
H	1.911648	9.967925	0.497527
H	3.342458	7.995321	0.918303
H	1.402254	-5.852302	-5.185241
H	0.279478	-5.080041	1.289958

Supplementary Table 3. Cartesian coordinates for the DFT calculated structure of ‘trans’-rctt-**2**-[Ox_2]

300
Energy = -6316.757023 H

C	-5.063830	1.809417	0.165375
C	2.845985	-4.153431	-0.230755
C	1.935496	-5.045354	-0.791415
C	0.637758	-4.615583	-1.065059
C	0.252140	-3.297494	-0.789733
C	1.203087	-2.426862	-0.262312
C	2.488319	-2.831736	0.046247
C	-1.136346	-2.813508	-0.935216
C	3.512183	-1.886851	0.652407
O	-0.321327	-5.435063	-1.608516
O	4.138488	-4.478318	0.107294
C	4.450923	-1.373688	-0.425555
C	2.807901	-0.797229	1.451449
C	-1.312593	-1.538072	-1.650939
C	-0.712199	-1.263893	-2.882645
C	-0.816943	0.000188	-3.449429
C	-1.498481	1.009957	-2.782606
C	-2.103867	0.774827	-1.541079
C	-2.001569	-0.500988	-1.011187
C	2.792098	0.523323	1.028540
C	2.006271	1.536574	1.603404
C	1.231826	1.180000	2.720861
C	1.263232	-0.127599	3.190598
C	1.996674	-1.116006	2.554279
C	1.908313	2.820332	0.874464
C	-2.748079	1.931486	-0.798485
C	3.004215	3.435603	0.316536
C	0.545025	3.322863	0.604106
C	-3.712365	1.522954	0.302708
C	-0.426859	2.452733	0.104008
C	-1.691010	2.878482	-0.257245
C	-2.003012	4.234408	-0.089086
C	-1.070263	5.126821	0.426483
C	0.191976	4.666391	0.781549
O	-0.018183	-2.271882	-3.534374
O	-1.520418	2.303413	-3.290234
O	-3.277887	4.603380	-0.444514
O	1.138464	5.470380	1.369935
O	0.338357	1.992362	3.381495
O	1.739310	-2.403449	2.994304

C	2.844912	4.452112	-0.700265
C	3.873080	5.147435	-1.229934
C	5.244673	4.857417	-0.761721
C	5.443741	3.715212	0.160652
C	4.361633	3.096019	0.683121
C	3.963995	-0.689432	-1.534082
C	4.806511	-0.152583	-2.500421
C	6.185805	-0.401741	-2.362019
C	6.709818	-1.176712	-1.310212
C	5.811880	-1.616349	-0.334439
C	-5.996171	1.492160	1.151845
C	-5.519963	0.835759	2.301799
C	-4.160726	0.503741	2.471665
C	-3.278959	0.892915	1.463339
C	-2.157698	-3.391038	-0.226060
C	-3.542808	-3.047549	-0.448400
C	-4.554950	-3.548054	0.296426
C	-4.245718	-4.510204	1.380072
C	-2.827866	-4.857402	1.630300
C	-1.874582	-4.322676	0.837356
C	6.869915	3.279300	0.510541
O	6.218495	5.557294	-1.137481
C	3.657230	6.237808	-2.285599
C	-3.642997	-0.254261	3.711966
O	-6.470100	0.547571	3.275998
C	-7.476057	1.878923	0.985582
C	4.233695	0.693154	-3.649648
O	7.003535	0.136354	-3.351492
C	8.193402	-1.596634	-1.241848
C	-6.018397	-3.196427	0.002055
O	-5.163915	-5.024963	2.069951
C	-2.462337	-5.782160	2.797252
C	6.879484	1.996824	1.363994
C	2.179309	6.333593	-2.706741
C	-3.837834	0.600921	4.988541
C	-7.731308	2.568808	-0.368435
C	-2.878725	-5.108394	4.123282
C	-6.135921	-2.102539	-1.076315
C	8.451039	-2.559464	-0.065647
C	2.696875	0.805657	-3.565537
C	4.485516	5.931750	-3.552374
C	-4.329108	-1.637123	3.835193
C	-8.379844	0.628513	1.034176
C	-3.167345	-7.149628	2.651921
C	-6.721186	-2.655967	1.265949

C	4.577381	0.054823	-5.013198
C	7.590405	4.392219	1.303269
C	4.072705	7.608399	-1.702982
C	-2.134096	-0.551665	3.609792
C	-7.874006	2.871548	2.100574
C	-0.946883	-6.046855	2.853706
C	-6.739243	-4.465554	-0.506964
C	4.803923	2.126467	-3.567224
C	7.641613	2.970199	-0.792896
C	8.577074	-2.370055	-2.527403
C	9.124860	-0.381871	-1.016248
C	-2.177978	2.503001	-4.586769
C	-0.665769	-2.791523	-4.755302
C	-0.123899	-6.873699	-1.631954
C	4.658979	-5.812521	-0.112210
C	0.608080	3.371575	3.736109
C	2.790975	-3.329418	3.387626
C	-3.729928	5.951737	-0.131160
C	1.032398	6.905488	1.305466
C	-1.448378	-7.501405	-1.965370
C	6.074452	-5.808135	0.391436
C	-1.588478	-8.348318	-3.063188
C	-2.820820	-8.935907	-3.345332
C	-3.920611	-8.677202	-2.532236
C	-3.787729	-7.821033	-1.438940
C	-2.558905	-7.234065	-1.161489
C	6.352011	-5.351771	1.682343
C	7.656398	-5.349183	2.162532
C	8.698233	-5.806669	1.356780
C	8.429025	-6.256519	0.068140
C	7.121589	-6.251722	-0.414591
C	-2.090657	-3.195126	-4.505145
C	-3.664816	2.299087	-4.485278
C	-2.384384	-4.407836	-3.880235
C	-3.703157	-4.759474	-3.612555
C	-4.739748	-3.895763	-3.958299
C	-4.453709	-2.671045	-4.558052
C	-3.134325	-2.325326	-4.828408
C	-4.293942	1.263009	-5.174163
C	-5.662641	1.044180	-5.030593
C	-6.412150	1.863245	-4.192108
C	-5.793595	2.912462	-3.513092
C	-4.428515	3.129636	-3.659970
C	-5.197723	6.025948	-0.426796
C	2.378139	7.492142	1.645936

C	-5.648568	6.097125	-1.746466
C	-7.010148	6.173622	-2.023246
C	-7.933925	6.190204	-0.979727
C	-7.491480	6.122299	0.337989
C	-6.128954	6.033487	0.611436
C	2.527909	8.880094	1.659850
C	3.773718	9.450122	1.901258
C	4.881597	8.636775	2.131238
C	4.731881	7.253298	2.130812
C	3.484820	6.681444	1.894195
C	-0.690547	4.135077	3.688543
C	2.173714	-4.350103	4.304581
C	1.233173	-3.971281	5.263966
C	0.678748	-4.920224	6.115676
C	1.063585	-6.256259	6.022231
C	1.998640	-6.640147	5.065382
C	2.545035	-5.691173	4.205118
C	-1.874587	3.525209	3.285181
C	-3.062446	4.249189	3.226844
C	-3.073078	5.596772	3.574817
C	-1.887629	6.215738	3.974225
C	-0.703257	5.488211	4.034122
H	-5.381127	2.313950	-0.734504
H	2.230162	-6.058182	-1.021546
H	0.907850	-1.409223	-0.034694
H	4.135501	-2.469142	1.333350
H	-0.328893	0.203998	-4.393496
H	-2.448773	-0.710328	-0.051393
H	3.353334	0.781209	0.143909
H	0.659721	-0.390252	4.045731
H	-3.326700	2.493682	-1.530040
H	-0.161219	1.412563	-0.038412
H	-1.333768	6.156995	0.606514
H	1.840423	4.626612	-1.051784
H	4.493127	2.322859	1.424578
H	2.895258	-0.566978	-1.633430
H	6.168289	-2.202295	0.499786
H	-2.220473	0.710056	1.585990
H	-3.754202	-2.390921	-1.279327
H	-0.834613	-4.537916	1.024207
H	-6.115052	0.008778	3.996402
H	7.940131	0.120197	-3.107889
H	6.396403	2.145863	2.335296
H	6.388753	1.164213	0.847513
H	7.917952	1.708966	1.556510

H	1.818915	5.394265	-3.139022
H	1.532788	6.600020	-1.863842
H	2.076152	7.115726	-3.464974
H	-4.875209	0.892354	5.170708
H	-3.485754	0.047003	5.864420
H	-3.255584	1.523156	4.905565
H	-7.490481	1.907468	-1.207376
H	-8.791626	2.831094	-0.437722
H	-7.156818	3.492964	-0.469411
H	-3.952252	-4.913789	4.134266
H	-2.624819	-5.758024	4.967909
H	-2.344108	-4.160820	4.247751
H	-5.637133	-1.177350	-0.765913
H	-5.718238	-2.421459	-2.034297
H	-7.195319	-1.874996	-1.232914
H	7.832668	-3.457392	-0.137979
H	8.266757	-2.080921	0.900921
H	9.498473	-2.874423	-0.087156
H	2.371412	1.297671	-2.642651
H	2.344734	1.416199	-4.403088
H	2.207828	-0.171468	-3.634916
H	4.333561	6.725772	-4.291744
H	4.166333	4.984350	-3.996261
H	5.547030	5.870930	-3.314805
H	-3.951655	-2.157056	4.720422
H	-4.092188	-2.242407	2.956621
H	-5.419029	-1.610143	3.910288
H	-9.426813	0.928711	0.912441
H	-8.270996	0.098465	1.978766
H	-8.121171	-0.054174	0.218985
H	-2.889872	-7.793714	3.493643
H	-2.853060	-7.645500	1.727095
H	-4.249047	-7.027225	2.637528
H	-7.776692	-2.467415	1.042904
H	-6.270807	-1.707078	1.568555
H	-6.652566	-3.366993	2.087565
H	4.152704	0.661259	-5.821013
H	4.148782	-0.950904	-5.078910
H	5.655896	-0.011143	-5.153270
H	8.612627	4.077920	1.541213
H	7.065557	4.585439	2.245496
H	7.630275	5.313082	0.721414
H	5.119912	7.597558	-1.402575
H	3.926199	8.387916	-2.459559
H	3.462173	7.852889	-0.829636

H	-1.898150	-1.166199	2.735067
H	-1.537347	0.362798	3.575416
H	-1.826511	-1.114185	4.497430
H	-7.764890	2.415182	3.084173
H	-7.242629	3.763966	2.048874
H	-8.916405	3.182119	1.966396
H	-0.729399	-6.715185	3.691113
H	-0.589167	-6.527901	1.935919
H	-0.373914	-5.130028	3.015968
H	-6.741121	-5.238152	0.263896
H	-7.775587	-4.228785	-0.771673
H	-6.232093	-4.853356	-1.397449
H	5.890857	2.124667	-3.646270
H	4.392990	2.729960	-4.383777
H	4.519547	2.597080	-2.620198
H	7.690489	3.849396	-1.433943
H	8.661443	2.655182	-0.547904
H	7.152397	2.157253	-1.339394
H	7.989577	-3.290880	-2.588317
H	8.395873	-1.802520	-3.442084
H	9.637476	-2.639906	-2.495401
H	10.160199	-0.724858	-0.926462
H	8.846417	0.132389	-0.093200
H	9.107643	0.368043	-1.813105
H	-1.923663	3.530741	-4.843290
H	-1.740839	1.832863	-5.332850
H	-0.605324	-2.032495	-5.541169
H	-0.040055	-3.639318	-5.028037
H	0.636199	-7.132679	-2.375541
H	0.224908	-7.198347	-0.644202
H	4.621038	-6.057699	-1.178087
H	4.040768	-6.532161	0.437991
H	1.337548	3.819753	3.057758
H	1.026909	3.378755	4.748136
H	3.590707	-2.769532	3.886701
H	3.204949	-3.829555	2.509631
H	-3.534559	6.154440	0.925672
H	-3.166790	6.665813	-0.741408
H	0.256386	7.243284	2.002155
H	0.741129	7.203157	0.290851
H	-0.735460	-8.550622	-3.701866
H	-2.920482	-9.592132	-4.201143
H	-4.877474	-9.134901	-2.751611
H	-4.640067	-7.602838	-0.806950
H	-2.460180	-6.550276	-0.329657

H	5.543016	-4.990266	2.305087
H	7.862003	-4.991239	3.163820
H	9.714523	-5.805256	1.730830
H	9.235222	-6.603321	-0.566282
H	6.917111	-6.591666	-1.423603
H	-1.580271	-5.062269	-3.570567
H	-3.918027	-5.701928	-3.127653
H	-5.767082	-4.171915	-3.751977
H	-5.253664	-1.984127	-4.805520
H	-2.908331	-1.367779	-5.281864
H	-3.713189	0.631172	-5.836746
H	-6.140718	0.237909	-5.573483
H	-7.475019	1.692573	-4.071689
H	-6.373092	3.560447	-2.867946
H	-3.948055	3.932767	-3.111943
H	-4.930161	6.100683	-2.558470
H	-7.349134	6.227494	-3.050526
H	-8.993408	6.254357	-1.194077
H	-8.205110	6.125700	1.152198
H	-5.786543	5.971557	1.638251
H	1.670345	9.517232	1.469255
H	3.881210	10.527992	1.902126
H	5.854478	9.079799	2.305302
H	5.589554	6.612915	2.297526
H	3.368811	5.607719	1.879498
H	0.923932	-2.935744	5.322965
H	-0.057242	-4.618605	6.850918
H	0.631001	-6.994432	6.686432
H	2.294067	-7.678720	4.979833
H	3.258528	-5.997968	3.447421
H	-1.861037	2.488558	2.989921
H	-3.968536	3.754159	2.895279
H	-3.996784	6.162840	3.545967
H	-1.888612	7.264324	4.247616
H	0.216441	5.975153	4.341247

Supplementary Table 4. Cartesian coordinates for the DFT calculated structure of ‘cis’-rctt-**2**-[Ox_2]

300
Energy = -6316.750976 H

C	4.264167	-1.851557	-0.040113
C	-1.105887	4.939202	0.991789
C	0.001427	5.657067	0.568808
C	1.092936	4.976692	0.043196
C	1.114406	3.590259	-0.068572
C	-0.027562	2.894583	0.347115
C	-1.141282	3.541749	0.869454
C	2.386320	2.907291	-0.564396
C	-2.445145	2.841524	1.234130
O	2.193213	5.725858	-0.380454
O	-2.229790	5.524702	1.534597
C	-3.455060	3.075491	0.120132
C	-2.270613	1.368644	1.572193
C	2.093149	1.636704	-1.346432
C	1.692483	1.713484	-2.683294
C	1.367221	0.570080	-3.407526
C	1.469626	-0.683686	-2.820851
C	1.905225	-0.799584	-1.496067
C	2.192085	0.370420	-0.787949
C	-2.778986	0.344969	0.782169
C	-2.570571	-1.011591	1.060916
C	-1.817971	-1.335871	2.198681
C	-1.321791	-0.334026	3.018424
C	-1.546256	1.001450	2.712943
C	-2.893838	-2.005807	0.011213
C	1.922175	-2.071574	-0.755980
C	-4.072185	-2.019623	-0.682897
C	-1.728143	-2.826669	-0.372972
C	2.939168	-2.409362	0.095538
C	-0.509260	-2.174095	-0.510109
C	0.668793	-2.843051	-0.776401
C	0.615022	-4.230493	-0.988473
C	-0.588459	-4.917719	-0.859243
C	-1.747078	-4.216753	-0.527641
O	1.642119	2.964160	-3.293240
O	1.136814	-1.834408	-3.519298
O	1.799143	-4.819272	-1.322715
O	-2.943113	-4.841214	-0.269404
O	-1.483399	-2.636424	2.537891
O	-1.017294	2.004327	3.522071
C	-4.189006	-2.721259	-1.942866

C	-5.325644	-2.761240	-2.667924
C	-6.528691	-2.073232	-2.146485
C	-6.403776	-1.273654	-0.903059
C	-5.234803	-1.289886	-0.227664
C	-3.171512	2.651233	-1.174076
C	-4.095269	2.747558	-2.206914
C	-5.313236	3.393950	-1.919759
C	-5.610618	3.911031	-0.644169
C	-4.665610	3.702266	0.366699
C	5.310043	-2.268393	0.708283
C	5.085783	-3.328148	1.720866
C	3.689976	-3.716527	2.035932
C	2.710246	-3.317705	1.195742
C	3.355790	2.686827	0.590443
C	4.670400	3.130473	0.496556
C	5.584740	2.959678	1.538755
C	5.149672	2.242895	2.667981
C	3.845673	1.722245	2.774465
C	2.957662	2.018857	1.742650
C	-7.610267	-0.470822	-0.408897
O	-7.629655	-2.161490	-2.743339
C	-5.405925	-3.485805	-4.015891
C	3.393542	-4.602112	3.254117
O	6.053428	-3.901750	2.283695
C	6.723364	-1.712744	0.503826
C	-3.788983	2.133364	-3.583621
O	-6.196793	3.523231	-2.987088
C	-6.877572	4.748392	-0.361637
C	6.994023	3.576271	1.474663
O	6.082394	2.093412	3.690164
C	3.388484	0.849405	3.960971
C	-8.762261	-1.431619	-0.037613
C	-4.051733	-4.104747	-4.406817
C	3.876781	-6.044850	2.980468
C	6.734844	-0.581317	-0.540760
C	1.981306	0.264109	3.726795
C	8.081709	2.489977	1.622285
C	-6.911431	5.239237	1.098183
C	-2.402665	1.454776	-3.612038
C	-5.804999	-2.484906	-5.123039
C	4.099893	-4.059246	4.514876
C	7.251807	-1.139622	1.836744
C	4.317110	-0.378680	4.133411
C	7.140592	4.637801	2.588693
C	-3.796448	3.223381	-4.677144

C	-8.068115	0.509135	-1.512926
C	-6.436889	-4.634604	-3.941782
C	1.884588	-4.644973	3.557985
C	7.666825	-2.829117	0.000357
C	3.313529	1.691227	5.257275
C	7.243144	4.281931	0.130711
C	-4.835949	1.041806	-3.902135
C	-7.262991	0.365739	0.836760
C	-6.864400	6.014612	-1.252939
C	-8.179680	3.935680	-0.560189
C	1.907649	-2.106163	-4.736706
C	2.590805	3.147850	-4.415601
C	2.078209	6.211901	-1.747677
C	-2.335310	6.967792	1.556435
C	-2.596176	-3.539457	2.873496
C	-1.737158	2.259264	4.787205
C	1.954487	-6.262113	-1.411206
C	-3.263500	-6.122810	-0.855663
C	3.378045	6.845200	-2.153833
C	-3.663492	7.323826	2.161728
C	3.446561	7.529519	-3.371010
C	4.649583	8.074654	-3.805643
C	5.795270	7.951504	-3.020234
C	5.725326	7.283766	-1.801438
C	4.522338	6.727343	-1.369431
C	-4.088065	6.699456	3.337224
C	-5.297458	7.056195	3.923656
C	-6.092151	8.044775	3.344987
C	-5.676407	8.666052	2.171557
C	-4.468666	8.302285	1.579249
C	4.023083	2.973442	-3.992094
C	3.373091	-2.295244	-4.447847
C	4.813001	4.084522	-3.691690
C	6.132801	3.923374	-3.279222
C	6.674872	2.646274	-3.156716
C	5.893070	1.531780	-3.449629
C	4.578260	1.695177	-3.868920
C	4.337119	-1.603265	-5.181000
C	5.692507	-1.771906	-4.901595
C	6.089494	-2.628893	-3.878325
C	5.128688	-3.323332	-3.144313
C	3.777774	-3.160680	-3.428718
C	3.422537	-6.495191	-1.628270
C	-4.763544	-6.282550	-0.846895
C	4.327408	-6.016197	-0.678790

C	5.693795	-6.195833	-0.849434
C	6.169966	-6.855294	-1.982957
C	5.273763	-7.326125	-2.938141
C	3.902428	-7.144728	-2.762846
C	-5.322571	-7.427951	-1.417536
C	-6.703125	-7.596011	-1.447187
C	-7.537680	-6.617779	-0.909911
C	-6.982241	-5.477113	-0.339162
C	-5.600468	-5.309962	-0.302778
C	-1.986085	-4.754631	3.500090
C	-1.630981	1.098371	5.733682
C	-2.700321	0.219462	5.906841
C	-2.555088	-0.925691	6.686474
C	-1.333764	-1.201104	7.296084
C	-0.263209	-0.321240	7.136506
C	-0.414233	0.823733	6.362705
C	-1.758507	-5.903165	2.742414
C	-1.155416	-7.018666	3.316626
C	-0.775008	-6.991074	4.655752
C	-0.992711	-5.843455	5.416517
C	-1.591104	-4.728788	4.838859
H	4.408515	-1.126946	-0.827740
H	0.051805	6.734183	0.639105
H	-0.049162	1.814623	0.252563
H	2.870074	3.594966	-1.260802
H	-2.829006	3.339233	2.129439
H	1.029619	0.662077	-4.431332
H	2.511856	0.267952	0.237987
H	-3.317649	0.593490	-0.119326
H	-0.746452	-0.613188	3.887144
H	-0.472591	-1.102228	-0.367603
H	-0.622799	-5.989989	-0.987809
H	-3.293533	-3.194443	-2.314445
H	-5.140917	-0.757103	0.706844
H	-2.202595	2.212992	-1.365898
H	-4.850757	4.072974	1.363718
H	1.684387	-3.607954	1.376485
H	4.970820	3.643926	-0.406994
H	1.924481	1.715432	1.833513
H	-7.075381	3.816510	-2.708168
H	5.705579	1.683310	4.480424
H	-9.064650	-2.021159	-0.903045
H	-8.447458	-2.109421	0.763606
H	-9.624314	-0.857482	0.319254
H	-3.266991	-3.347319	-4.502960

H	-3.731036	-4.854991	-3.676615
H	-4.156074	-4.605261	-5.374123
H	4.940803	-6.054046	2.737940
H	3.711103	-6.664287	3.868870
H	3.318215	-6.488782	2.150123
H	6.098566	0.259820	-0.244739
H	7.757672	-0.206423	-0.647051
H	6.409595	-0.943825	-1.521144
H	1.946661	-0.342722	2.815975
H	1.725803	-0.387816	4.568634
H	1.210901	1.036898	3.669271
H	7.983901	1.957162	2.566539
H	8.003311	1.767335	0.804815
H	9.072997	2.955266	1.575884
H	-6.036258	5.843562	1.338985
H	-6.970890	4.404790	1.803949
H	-7.796199	5.865854	1.243847
H	-2.333733	0.636372	-2.887348
H	-2.243150	1.027465	-4.607033
H	-1.590284	2.163377	-3.420587
H	-5.865687	-3.004673	-6.085444
H	-5.055306	-1.692015	-5.207001
H	-6.773312	-2.033557	-4.906763
H	3.866069	-4.706290	5.367049
H	3.741915	-3.052749	4.745484
H	5.180395	-4.029309	4.380421
H	8.275537	-0.774577	1.701229
H	7.253516	-1.904168	2.615091
H	6.636755	-0.294977	2.160775
H	3.984721	-0.959771	4.998015
H	5.372578	-0.146115	4.279131
H	4.252564	-1.014507	3.245559
H	8.133834	5.097142	2.532046
H	7.016214	4.190660	3.574445
H	6.390716	5.425733	2.459818
H	-3.571306	2.772507	-5.650135
H	-3.030726	3.976883	-4.463992
H	-4.767713	3.713985	-4.732395
H	-8.935720	1.077114	-1.160773
H	-8.344321	-0.027620	-2.419222
H	-7.266616	1.216383	-1.748039
H	-7.423860	-4.252361	-3.685797
H	-6.491087	-5.137238	-4.914262
H	-6.136272	-5.368661	-3.189891
H	1.472460	-3.640903	3.699888

H	1.309426	-5.143249	2.771177
H	1.718922	-5.211036	4.478157
H	7.739502	-3.630663	0.734388
H	7.299359	-3.243044	-0.942648
H	8.665322	-2.413679	-0.175584
H	4.262871	2.155380	5.542730
H	2.587908	2.499757	5.128131
H	2.990534	1.061041	6.092487
H	6.535745	5.097437	-0.039557
H	8.252061	4.706656	0.133004
H	7.169799	3.583833	-0.705898
H	-5.842867	1.456946	-3.930993
H	-4.613373	0.593091	-4.876368
H	-4.803473	0.249112	-3.146646
H	-6.464093	1.086550	0.629165
H	-8.151446	0.926114	1.143739
H	-6.964381	-0.262376	1.682564
H	-6.004551	6.637204	-0.988824
H	-6.794775	5.786987	-2.318906
H	-7.775590	6.597859	-1.085176
H	-9.040011	4.560215	-0.300692
H	-8.174296	3.062193	0.095214
H	-8.354291	3.567197	-1.575342
H	1.448953	-3.015624	-5.124999
H	1.752880	-1.300676	-5.461285
H	2.391387	4.163556	-4.753249
H	2.325788	2.447633	-5.212459
H	1.838119	5.361943	-2.399682
H	1.254287	6.932907	-1.808208
H	-2.247642	7.360235	0.537907
H	-1.515785	7.378495	2.158800
H	-3.151771	-3.792059	1.967484
H	-3.255798	-3.016218	3.573124
H	-2.782554	2.485013	4.554198
H	-1.252756	3.153232	5.176323
H	1.612568	-6.708873	-0.470692
H	1.352124	-6.654531	-2.235904
H	-2.781069	-6.920273	-0.278341
H	-2.876908	-6.157925	-1.880784
H	2.556072	7.626875	-3.983693
H	4.694440	8.596757	-4.753670
H	6.732575	8.378281	-3.355541
H	6.610718	7.191860	-1.183577
H	4.462400	6.193696	-0.430342
H	-3.468799	5.930963	3.783099

H	-5.620440	6.565464	4.833723
H	-7.032925	8.323117	3.803744
H	-6.293916	9.427060	1.710622
H	-4.152233	8.782595	0.659974
H	4.401839	5.081551	-3.786635
H	6.729477	4.797573	-3.051253
H	7.701746	2.519451	-2.835067
H	6.304238	0.533969	-3.363893
H	3.985625	0.819325	-4.101081
H	4.030778	-0.926555	-5.972041
H	6.432437	-1.228388	-5.476634
H	7.141705	-2.757532	-3.653212
H	5.425081	-3.997451	-2.351358
H	3.039831	-3.700166	-2.847288
H	3.955364	-5.477627	0.180798
H	6.379008	-5.812397	-0.103470
H	7.234896	-6.995625	-2.122401
H	5.640084	-7.829319	-3.824471
H	3.208789	-7.504878	-3.514588
H	-4.677889	-8.188762	-1.845655
H	-7.127231	-8.485796	-1.896210
H	-8.612921	-6.742478	-0.943408
H	-7.623732	-4.704971	0.068195
H	-5.168897	-4.418280	0.127581
H	-3.643147	0.420379	5.410348
H	-3.389809	-1.604801	6.810561
H	-1.216282	-2.094533	7.897196
H	0.686954	-0.530215	7.612622
H	0.422629	1.497832	6.220711
H	-2.068206	-5.915065	1.704981
H	-0.985221	-7.908100	2.721975
H	-0.307217	-7.858491	5.105360
H	-0.693208	-5.818148	6.457220
H	-1.749356	-3.829104	5.424380

Supplementary Table 5. Cartesian coordinates for the DFT calculated structure of ‘gem’-rctt-**2**-[Ox$_2$]

300
Energy = -6316.743863 H

C	2.492340	-4.076587	0.086071
C	1.414777	4.564267	0.110377
C	2.792669	4.524584	-0.055765
C	3.409621	3.340954	-0.419677
C	2.687026	2.164775	-0.623972
C	1.305062	2.231155	-0.485368
C	0.636155	3.414530	-0.146687
C	3.443670	0.878020	-0.921126
C	-0.828260	3.436076	0.042182
O	4.795723	3.323534	-0.519576
O	0.766808	5.674404	0.585879
C	-1.604408	4.473394	-0.412193
C	-1.443221	2.255353	0.699801
C	2.603102	-0.088875	-1.728705
C	2.468611	0.121147	-3.103960
C	1.731016	-0.752374	-3.893112
C	1.126996	-1.857013	-3.311775
C	1.227409	-2.089194	-1.938293
C	1.955723	-1.186424	-1.169875
C	-2.367196	1.496108	-0.033941
C	-2.851953	0.259041	0.403657
C	-2.445399	-0.186250	1.671115
C	-1.567875	0.565088	2.432092
C	-1.036386	1.752931	1.946567
C	-3.602277	-0.625396	-0.523275
C	0.566578	-3.316562	-1.355516
C	-4.764595	-0.239871	-1.134338
C	-2.970179	-1.931927	-0.798604
C	1.139163	-3.761091	-0.012814
C	-1.590607	-2.003548	-1.027169
C	-0.939521	-3.194163	-1.288403
C	-1.706372	-4.364180	-1.348117
C	-3.070938	-4.345228	-1.089896
C	-3.691933	-3.138555	-0.779575
O	3.086149	1.235650	-3.674164
O	0.382213	-2.746567	-4.085814
O	-1.015213	-5.515828	-1.629809
O	-5.009937	-3.075062	-0.395277
O	-2.877527	-1.379045	2.213630
O	-0.067089	2.326151	2.734904

C	-5.300944	-0.972743	-2.258964
C	-6.517110	-0.731924	-2.790118
C	-7.354650	0.337345	-2.199841
C	-6.797887	1.135823	-1.080831
C	-5.557173	0.855292	-0.624351
C	-1.146215	5.343277	-1.472624
C	-1.880319	6.365714	-1.960337
C	-3.163346	6.693723	-1.299491
C	-3.559395	5.923041	-0.095093
C	-2.866576	4.804293	0.217529
C	3.049487	-4.588626	1.254761
C	2.169687	-4.868336	2.321495
C	0.796386	-4.576066	2.257997
C	0.327522	-3.971603	1.089628
C	4.036452	0.304240	0.360771
C	5.393407	-0.003701	0.406269
C	6.024927	-0.406376	1.583787
C	5.224098	-0.521154	2.734379
C	3.830588	-0.323393	2.707601
C	3.272692	0.126185	1.508826
C	-7.653170	2.244816	-0.462279
O	-8.508091	0.569001	-2.639051
C	-7.063946	-1.548081	-3.965690
C	-0.205456	-4.948453	3.373175
O	2.731125	-5.485353	3.438975
C	4.560015	-4.873226	1.340766
C	-1.474644	7.125224	-3.230336
O	-3.908271	7.598095	-1.751782
C	-4.710268	6.453522	0.769492
C	7.548448	-0.627978	1.632146
O	5.892288	-0.831439	3.914003
C	2.942793	-0.576284	3.944348
C	-8.956209	1.655362	0.121382
C	-6.043393	-2.593890	-4.451435
C	-1.659948	-4.670621	2.938314
C	5.317349	-4.286104	0.131320
C	1.448085	-0.441941	3.599134
C	8.165235	-0.607330	0.223638
C	-4.875695	5.628044	2.059141
C	-0.069448	6.713413	-3.705581
C	-7.391742	-0.618991	-5.155575
C	-0.135575	-6.464502	3.686383
C	5.181011	-4.229224	2.598368
C	3.123868	-2.024020	4.464556
C	7.910086	-2.002871	2.234834

C	-1.459963	8.654000	-3.008752
C	-7.981575	3.309595	-1.533520
C	-8.333955	-2.307734	-3.516558
C	0.044748	-4.102549	4.643909
C	4.789442	-6.399635	1.356450
C	3.233051	0.468659	5.050878
C	8.192632	0.505243	2.461715
C	-2.482251	6.781590	-4.352842
C	-6.903736	2.941546	0.683508
C	-4.379478	7.900414	1.205983
C	-6.042879	6.441803	-0.011002
C	1.148348	-3.537782	-5.062618
C	4.083601	0.956566	-4.717981
C	5.327384	3.762206	-1.803630
C	1.547129	6.794308	1.103230
C	-4.324904	-1.541438	2.436588
C	-0.084117	3.735238	3.103795
C	-1.518562	-6.813035	-1.196693
C	-5.834128	-4.258462	-0.402270
C	6.826992	3.697346	-1.750695
C	0.554491	7.838037	1.511089
C	7.575342	4.322340	-2.750446
C	8.963846	4.236614	-2.748734
C	9.618411	3.534927	-1.737490
C	8.874505	2.920966	-0.734042
C	7.483468	2.996311	-0.741740
C	0.190408	8.011407	2.844335
C	-0.764489	8.963783	3.192751
C	-1.366171	9.741023	2.206398
C	-1.012004	9.564803	0.869378
C	-0.053612	8.619267	0.528679
C	5.369502	0.431498	-4.143073
C	2.160242	-4.420581	-4.387104
C	6.498077	1.249157	-4.093644
C	7.694248	0.781636	-3.556967
C	7.770909	-0.517599	-3.067905
C	6.643931	-1.339120	-3.095103
C	5.450393	-0.869030	-3.630100
C	3.513700	-4.081160	-4.394251
C	4.441392	-4.856028	-3.701730
C	4.017136	-5.977138	-2.992289
C	2.666677	-6.322465	-2.977118
C	1.745130	-5.550870	-3.676514
C	-0.325582	-7.700756	-0.990015
C	-7.188013	-3.889349	0.141165

C	0.589044	-7.378574	0.015676
C	1.694023	-8.187099	0.247789
C	1.905788	-9.319940	-0.538515
C	1.007711	-9.635779	-1.554940
C	-0.106256	-8.827255	-1.779805
C	-7.656393	-2.576209	0.100473
C	-8.931461	-2.276016	0.571293
C	-9.742677	-3.279032	1.093845
C	-9.272839	-4.589323	1.147815
C	-8.000445	-4.892717	0.674351
C	-4.494183	-2.586740	3.499733
C	1.305596	4.085492	3.553820
C	1.505245	5.049742	4.542806
C	2.794913	5.443285	4.892336
C	3.896545	4.859276	4.269598
C	3.701994	3.870670	3.307805
C	2.412507	3.485494	2.953378
C	-5.354514	-3.663554	3.298631
C	-5.505988	-4.638044	4.283408
C	-4.791873	-4.543095	5.473741
C	-3.930497	-3.464900	5.680578
C	-3.784726	-2.492031	4.699556
H	3.117830	-3.905288	-0.778718
H	3.416535	5.380910	0.150943
H	0.715576	1.337594	-0.652601
H	4.290325	1.148995	-1.555669
H	1.614833	-0.569838	-4.953406
H	2.067258	-1.380953	-0.112060
H	-2.647152	1.843563	-1.021095
H	-1.278292	0.213669	3.410542
H	0.774920	-4.135449	-2.043868
H	-1.009650	-1.090294	-0.981561
H	-3.639429	-5.262115	-1.121499
H	-4.662097	-1.731545	-2.684595
H	-5.139398	1.407126	0.204887
H	-0.207588	5.074423	-1.934749
H	-3.164403	4.189677	1.056691
H	-0.713506	-3.705332	1.019907
H	5.971689	0.116137	-0.500246
H	2.218439	0.365435	1.473306
H	2.185787	-5.377263	4.231874
H	5.310688	-0.804525	4.686037
H	-9.535971	1.150857	-0.651125
H	-8.721496	0.943030	0.919874
H	-9.563481	2.459177	0.551342

H	-5.120035	-2.127299	-4.809579
H	-5.789523	-3.308909	-3.661450
H	-6.479542	-3.155684	-5.282685
H	-1.915753	-5.227892	2.030971
H	-2.338869	-4.990634	3.733858
H	-1.848774	-3.609643	2.760941
H	5.171692	-3.202736	0.058264
H	6.387777	-4.474862	0.263533
H	5.014197	-4.746325	-0.812073
H	1.156049	-1.137263	2.805133
H	0.859216	-0.687556	4.489833
H	1.186347	0.576119	3.304481
H	7.734272	-1.395857	-0.398938
H	8.026495	0.345675	-0.289839
H	9.242102	-0.788921	0.304127
H	-5.137658	4.586996	1.859909
H	-5.683373	6.061922	2.656437
H	-3.961075	5.649001	2.661249
H	-0.026294	5.658120	-3.992752
H	0.198863	7.307748	-4.584083
H	0.682119	6.895981	-2.929659
H	-7.768349	-1.213844	-5.994679
H	-6.490575	-0.092149	-5.486869
H	-8.147882	0.114268	-4.876935
H	-0.838238	-6.703344	4.490920
H	0.855473	-6.807451	3.985753
H	-0.422298	-7.039149	2.800584
H	6.246927	-4.475268	2.640230
H	4.702192	-4.583732	3.507932
H	5.091296	-3.141515	2.548218
H	2.500753	-2.169222	5.352948
H	4.149573	-2.292157	4.723957
H	2.798830	-2.728336	3.695186
H	9.000690	-2.104166	2.273752
H	7.515645	-2.802075	1.601401
H	7.505723	-2.121701	3.237461
H	-1.216358	9.153706	-3.952481
H	-0.696820	8.931729	-2.277467
H	-2.429532	9.006009	-2.661030
H	-8.556296	4.124558	-1.080689
H	-8.564172	2.873616	-2.344464
H	-7.059136	3.731206	-1.946437
H	-9.104813	-1.612434	-3.185366
H	-8.724025	-2.898764	-4.352452
H	-8.099218	-2.987489	-2.691080

H	1.025559	-4.265576	5.104139
H	-0.024547	-3.038836	4.400083
H	-0.712605	-4.335395	5.398946
H	4.292533	-6.856055	2.214958
H	4.398309	-6.848885	0.437347
H	5.861794	-6.616819	1.417045
H	4.272719	0.487301	5.392577
H	3.002614	1.470789	4.680299
H	2.603550	0.267790	5.923583
H	7.822292	0.493870	3.487479
H	9.281448	0.381836	2.478271
H	7.963509	1.479431	2.016159
H	-3.487776	7.101682	-4.077701
H	-2.192017	7.289527	-5.279019
H	-2.491215	5.702891	-4.539997
H	-5.961254	3.370187	0.332457
H	-7.515629	3.759074	1.074955
H	-6.690869	2.253842	1.509160
H	-3.447498	7.922975	1.777757
H	-4.274973	8.557310	0.342563
H	-5.185576	8.282164	1.842281
H	-6.281940	5.435144	-0.360420
H	-5.980532	7.103979	-0.873630
H	-6.855846	6.778283	0.642072
H	0.383540	-4.113619	-5.581941
H	1.632670	-2.862460	-5.774577
H	3.657356	0.255008	-5.440781
H	4.233637	1.917562	-5.207988
H	4.931659	3.104929	-2.587974
H	4.983829	4.783066	-2.006792
H	2.197334	7.170768	0.307766
H	2.160636	6.450144	1.941899
H	-4.809520	-1.843019	1.507058
H	-4.725859	-0.572845	2.756035
H	-0.816542	3.880403	3.904108
H	-0.369513	4.353078	2.247848
H	-2.067655	-6.679852	-0.258137
H	-2.199955	-7.211570	-1.952677
H	-5.375283	-5.036355	0.218644
H	-5.913533	-4.634169	-1.430086
H	7.070709	4.878489	-3.533953
H	9.534783	4.723768	-3.529898
H	10.699780	3.473368	-1.730344
H	9.376055	2.375819	0.056833
H	6.896560	2.513429	0.028447

H	0.663679	7.405963	3.607428
H	-1.038629	9.098331	4.231966
H	-2.113417	10.477217	2.476111
H	-1.485988	10.157440	0.096809
H	0.228776	8.473511	-0.506587
H	6.444639	2.262599	-4.475092
H	8.554048	1.438020	-3.509298
H	8.700503	-0.885780	-2.652752
H	6.693604	-2.344961	-2.695295
H	4.579451	-1.512682	-3.643169
H	3.843255	-3.208232	-4.947266
H	5.489721	-4.583325	-3.714892
H	4.735400	-6.581399	-2.451160
H	2.331900	-7.187966	-2.420019
H	0.693068	-5.810101	-3.643131
H	0.440823	-6.483731	0.605690
H	2.386867	-7.929502	1.038463
H	2.767925	-9.951203	-0.360608
H	1.172484	-10.509835	-2.172894
H	-0.805690	-9.076429	-2.570167
H	-7.022685	-1.798223	-0.298986
H	-9.291739	-1.256295	0.521435
H	-10.734132	-3.041760	1.459297
H	-9.895009	-5.373918	1.560584
H	-7.636254	-5.913619	0.722265
H	0.650557	5.488117	5.047398
H	2.938891	6.196219	5.657602
H	4.899398	5.164380	4.541944
H	4.548077	3.397956	2.823664
H	2.263831	2.711664	2.212993
H	-5.914513	-3.736712	2.373985
H	-6.179630	-5.469783	4.116080
H	-4.905248	-5.301888	6.238488
H	-3.373529	-3.386247	6.606517
H	-3.101981	-1.664487	4.848582

Supplementary Table 6. Cartesian coordinates for the DFT calculated structure of 'trans'-rccc-**2**-[Ox_2]

300
Energy = -6316.753347 H

C	-0.495216	-4.837315	-1.821015
C	3.396833	3.002078	-0.108112
C	4.655877	2.632985	0.340151
C	4.895183	1.317006	0.721208
C	3.899687	0.343415	0.642882
C	2.635004	0.751890	0.235050
C	2.360034	2.059234	-0.152147
C	4.216019	-1.127891	0.885200
C	0.972512	2.446869	-0.472590
O	6.154965	0.989814	1.223620
O	3.061683	4.282249	-0.466920
C	0.615801	3.050464	-1.647049
C	-0.039341	2.045785	0.520177
C	3.014409	-1.842974	1.484062
C	2.716110	-1.669025	2.844979
C	1.569562	-2.227416	3.398713
C	0.687749	-2.952941	2.605826
C	0.951081	-3.147666	1.245263
C	2.129528	-2.590717	0.724805
C	-1.163577	1.342487	0.086474
C	-2.131075	0.859742	0.955561
C	-1.935215	1.090338	2.317867
C	-0.831070	1.794877	2.785148
C	0.115829	2.276591	1.892989
C	-3.307636	0.024290	0.487058
C	-0.023259	-3.774903	0.335339
C	-3.749158	0.314855	-0.943369
C	-2.987629	-1.456485	0.611790
C	0.369145	-4.597617	-0.689440
C	-1.701590	-1.958460	0.505399
C	-1.425599	-3.326688	0.477138
C	-2.495416	-4.218048	0.622522
C	-3.798466	-3.741083	0.753441
C	-4.038382	-2.372095	0.726429
O	3.592518	-0.888915	3.564763
O	-0.444176	-3.541412	3.147713
O	-2.184318	-5.559118	0.644088
O	-5.291783	-1.818597	0.806138
O	-2.861891	0.569765	3.219141
O	1.222099	2.989447	2.320878

C	-2.981831	-0.117516	-2.020914
C	-3.323515	0.155338	-3.340095
C	-4.541649	0.822255	-3.560200
C	-5.389457	1.209836	-2.504947
C	-4.942929	0.973812	-1.199948
C	1.523612	3.101648	-2.768239
C	1.194383	3.651584	-3.957487
C	-0.138853	4.285220	-4.113348
C	-1.072228	4.259678	-2.964991
C	-0.685031	3.655951	-1.821248
C	-0.102226	-5.516363	-2.920636
C	1.259004	-6.099107	-2.956931
C	2.103944	-5.984318	-1.746740
C	1.666046	-5.233697	-0.711228
C	4.711394	-1.746527	-0.417381
C	5.869104	-2.505158	-0.452587
C	6.368132	-3.074013	-1.631904
C	5.634245	-2.853001	-2.808473
C	4.446716	-2.085242	-2.815188
C	4.025980	-1.540581	-1.610214
C	-6.786586	1.814352	-2.758313
O	-4.877262	1.044953	-4.892979
C	-2.385696	-0.250354	-4.489511
C	3.435170	-6.741773	-1.688604
O	1.684539	-6.681398	-3.987739
C	-1.011734	-5.657158	-4.146262
C	2.151331	3.623880	-5.154349
O	-0.462446	4.839541	-5.194266
C	-2.433762	4.952917	-3.083603
C	7.708744	-3.837873	-1.513162
O	6.126252	-3.404919	-3.982033
C	3.619286	-1.839946	-4.094153
C	-7.643959	0.826552	-3.587317
C	-1.124695	-0.964425	-3.966746
C	3.173866	-8.254311	-1.866734
C	-1.205079	-7.148732	-4.500804
C	4.463067	-1.114698	-5.171491
C	7.602613	-4.893710	-0.387551
C	-3.265059	4.790707	-1.796518
C	1.509430	2.808272	-6.299457
C	-3.098101	-1.218091	-5.459142
C	4.390097	-6.236162	-2.790600
C	-0.383272	-4.901957	-5.339738
C	3.029665	-3.175399	-4.615784
C	8.805987	-2.809094	-1.150803

C	2.460010	5.062356	-5.629983
C	-6.694845	3.199091	-3.445142
C	-1.905844	1.014047	-5.238502
C	4.140526	-6.538914	-0.335437
C	-2.407690	-5.059245	-3.889434
C	2.406242	-0.929052	-3.817256
C	8.176218	-4.594532	-2.772127
C	3.492352	2.958381	-4.794463
C	-7.545069	2.050455	-1.439877
C	-2.220581	6.464078	-3.328473
C	-3.245762	4.337769	-4.243728
C	-3.647141	1.568915	3.972434
C	0.971716	4.208047	3.091719
C	-1.426523	-2.667299	3.797449
C	3.678957	-0.983363	5.019628
C	3.197032	0.292461	5.651892
C	-1.049141	-2.329029	5.215372
C	0.088351	5.179166	2.356987
C	-4.477229	2.423903	3.060177
C	-3.930921	3.572810	2.482764
C	-4.684216	4.351822	1.611683
C	-5.993639	3.988421	1.310604
C	-6.543146	2.836087	1.870786
C	-5.785324	2.055851	2.737939
C	-1.042922	5.717726	2.969212
C	-1.855836	6.618335	2.282163
C	-1.541165	6.980387	0.974808
C	-0.411529	6.442126	0.359222
C	0.399211	5.546190	1.045801
C	-0.446529	-3.282889	6.039248
C	-0.078503	-2.954231	7.339972
C	-0.317049	-1.671752	7.832101
C	-0.927537	-0.722163	7.017941
C	-1.287030	-1.047559	5.712313
C	4.036402	1.020888	6.492898
C	3.606542	2.218158	7.059650
C	2.331005	2.698686	6.783491
C	1.486062	1.972647	5.944993
C	1.915494	0.779228	5.377562
C	-6.470517	-2.662957	0.722883
C	-3.189649	-6.527123	1.027912
C	6.233627	1.115628	2.701266
C	3.993210	5.380359	-0.314449
C	6.041616	2.536351	3.148363
C	-7.652467	-1.743279	0.626591

C	-3.633631	-6.369448	2.462829
C	-7.937968	-0.873853	1.681785
C	-9.028151	-0.013802	1.611187
C	-9.849387	-0.019922	0.484133
C	-9.569294	-0.882309	-0.571379
C	-8.469676	-1.735905	-0.502703
C	-2.751719	-5.876279	3.425795
C	-3.169107	-5.735733	4.746375
C	-4.463502	-6.094344	5.117042
C	-5.343367	-6.590613	4.159006
C	-4.930661	-6.721764	2.835377
C	7.054833	3.477579	2.944712
C	6.864457	4.808698	3.298206
C	5.660875	5.208217	3.879708
C	4.650148	4.274555	4.088969
C	4.829796	2.947750	3.703517
C	3.260236	6.607749	-0.784393
C	2.454890	6.534835	-1.923703
C	1.761246	7.654673	-2.368204
C	1.867274	8.861141	-1.676651
C	2.666679	8.937185	-0.539876
C	3.358099	7.812698	-0.092676
H	-1.472424	-4.380390	-1.787312
H	5.455926	3.350596	0.442586
H	1.824658	0.032753	0.224489
H	5.030590	-1.185405	1.609085
H	1.337193	-2.103405	4.444399
H	2.328835	-2.719917	-0.329739
H	-1.273672	1.173803	-0.974411
H	-0.707482	1.954552	3.847761
H	-4.148711	0.240279	1.147699
H	-0.870745	-1.268342	0.423736
H	-4.614997	-4.430566	0.896697
H	-2.090228	-0.690994	-1.817170
H	-5.547841	1.275092	-0.356430
H	2.485916	2.630734	-2.629851
H	-1.333964	3.666157	-0.959605
H	2.267581	-5.137189	0.180362
H	6.419216	-2.640105	0.470268
H	3.149204	-0.913654	-1.592821
H	-5.577738	1.704630	-4.994375
H	5.467239	-3.408538	-4.690234
H	-7.793304	-0.092554	-3.013517
H	-8.624964	1.268329	-3.790266
H	-7.187778	0.552252	-4.539985

H	-0.531999	-0.313366	-3.315702
H	-1.357636	-1.886557	-3.423992
H	-0.498126	-1.236588	-4.820636
H	2.710574	-8.454864	-2.833007
H	4.121432	-8.801125	-1.811336
H	2.516621	-8.625181	-1.072983
H	-0.250965	-7.616381	-4.739419
H	-1.662423	-7.682276	-3.660558
H	-1.871007	-7.237784	-5.366072
H	5.376975	-1.642320	-5.449235
H	3.864274	-0.973560	-6.076616
H	4.760445	-0.129447	-4.800297
H	7.355516	-4.456913	0.582668
H	6.836047	-5.632234	-0.635619
H	8.561022	-5.413057	-0.283487
H	-3.473737	3.738240	-1.574318
H	-4.225222	5.300315	-1.928971
H	-2.766233	5.240730	-0.932382
H	0.565640	3.256135	-6.611104
H	2.187741	2.776921	-7.158993
H	1.321818	1.780119	-5.971029
H	-2.407690	-1.518738	-6.255085
H	-3.972293	-0.748047	-5.907965
H	-3.416335	-2.120885	-4.927607
H	5.341518	-6.777364	-2.732654
H	4.597657	-5.173979	-2.646480
H	3.954381	-6.386703	-3.777630
H	-1.034598	-4.983883	-6.216895
H	0.595035	-5.316397	-5.584439
H	-0.268271	-3.840240	-5.096348
H	2.396127	-3.622907	-3.844740
H	3.771040	-3.932612	-4.886400
H	2.414149	-2.990643	-5.501435
H	9.770368	-3.315986	-1.036000
H	8.578913	-2.288784	-0.215975
H	8.901315	-2.060224	-1.943008
H	3.153992	5.027519	-6.476801
H	2.932815	5.638076	-4.826993
H	1.548043	5.570178	-5.940091
H	-7.698537	3.621713	-3.553568
H	-6.244847	3.195266	-4.442534
H	-6.094641	3.875979	-2.831680
H	-2.743754	1.552356	-5.679820
H	-1.382530	1.685612	-4.549332
H	-1.208180	0.731172	-6.034648

H	5.071886	-7.113305	-0.333740
H	4.397073	-5.487196	-0.167965
H	3.529940	-6.894294	0.501100
H	-3.027083	-5.207816	-4.779168
H	-2.905950	-5.545362	-3.043892
H	-2.363238	-3.983105	-3.696756
H	1.879486	-0.745598	-4.757567
H	1.698498	-1.397272	-3.127115
H	2.704265	0.041427	-3.409926
H	8.369331	-3.921196	-3.605379
H	9.104750	-5.121555	-2.524143
H	7.436265	-5.327562	-3.096342
H	4.143762	2.973727	-5.673367
H	4.005706	3.490185	-3.986244
H	3.360770	1.913189	-4.498797
H	-7.041904	2.798851	-0.825897
H	-8.550907	2.416997	-1.667315
H	-7.648890	1.130800	-0.860603
H	-1.661235	6.908778	-2.498272
H	-1.670862	6.630335	-4.255175
H	-3.190519	6.968796	-3.397446
H	-3.457791	3.284996	-4.038430
H	-2.699937	4.413097	-5.183332
H	-4.199344	4.867791	-4.343289
H	-4.258587	0.963654	4.639008
H	-2.961336	2.177760	4.569522
H	1.973951	4.606846	3.240845
H	0.548900	3.955024	4.067894
H	-2.347374	-3.250961	3.753384
H	-1.562359	-1.763925	3.198664
H	3.108856	-1.847900	5.367549
H	4.732018	-1.157907	5.243265
H	-2.911515	3.861439	2.709115
H	-4.246047	5.240180	1.174322
H	-6.584434	4.601471	0.640832
H	-7.555697	2.539703	1.625683
H	-6.210846	1.157669	3.170268
H	-1.292416	5.433434	3.986647
H	-2.733597	7.029573	2.765945
H	-2.171414	7.678488	0.436786
H	-0.152433	6.719478	-0.654454
H	1.266570	5.120166	0.556065
H	-0.237899	-4.269594	5.642787
H	0.397104	-3.696573	7.969391
H	-0.022099	-1.413913	8.841772

H	-1.105746	0.278167	7.393147
H	-1.751604	-0.313022	5.063926
H	5.037989	0.658735	6.695849
H	4.272814	2.779976	7.701915
H	1.993691	3.630557	7.220689
H	0.483646	2.333046	5.747903
H	1.253017	0.229475	4.719870
H	-6.526373	-3.288769	1.620745
H	-6.398603	-3.311635	-0.155719
H	-2.686411	-7.482188	0.873250
H	-4.043773	-6.479097	0.343112
H	7.230071	0.744242	2.937268
H	5.474306	0.463297	3.140280
H	4.299614	5.467729	0.733834
H	4.882819	5.186371	-0.925161
H	-7.302228	-0.879434	2.559133
H	-9.244667	0.652725	2.437512
H	-10.701004	0.646849	0.429421
H	-10.198362	-0.885198	-1.452810
H	-8.248409	-2.399541	-1.330982
H	-1.753140	-5.571511	3.133665
H	-2.484431	-5.338143	5.486340
H	-4.785744	-5.981905	6.144934
H	-6.352855	-6.866537	4.438477
H	-5.624116	-7.095047	2.088738
H	7.992152	3.162406	2.499272
H	7.653742	5.531700	3.132018
H	5.516923	6.240939	4.173941
H	3.724852	4.570575	4.566878
H	4.023849	2.234225	3.825411
H	2.349184	5.590082	-2.439350
H	1.134961	7.580725	-3.249547
H	1.325330	9.734207	-2.019132
H	2.745121	9.868869	0.006990
H	3.965197	7.872831	0.804075

Supplementary Table 7. Cartesian coordinates for the DFT calculated structure of ‘cis’-rccc-**2**-[Ox_2]

300
Energy = -6316.746990 H

C	2.885497	-3.932789	-2.602846
C	-0.145478	4.875070	0.089288
C	1.142773	5.331899	-0.135289
C	2.212810	4.452158	-0.095488
C	2.008781	3.088274	0.146656
C	0.698104	2.657866	0.358627
C	-0.393279	3.526962	0.353309
C	3.210825	2.145459	0.219969
C	-1.820950	3.089762	0.682734
O	3.481626	4.992146	-0.308823
O	-1.198665	5.787233	0.125512
C	-2.771143	3.330955	-0.476940
C	-1.865269	1.649113	1.170119
C	2.852835	0.758720	0.735657
C	2.557351	0.573068	2.085570
C	2.191749	-0.664572	2.597595
C	2.163740	-1.778433	1.768272
C	2.473464	-1.637197	0.407364
C	2.805397	-0.372016	-0.074122
C	-2.260019	0.597152	0.360609
C	-2.209232	-0.741925	0.768332
C	-1.730073	-1.008167	2.051670
C	-1.321398	0.020671	2.886158
C	-1.399840	1.340914	2.458984
C	-2.513452	-1.811377	-0.197394
C	2.284167	-2.754548	-0.545834
C	-3.610768	-1.784770	-1.015427
C	-1.461652	-2.838478	-0.322752
C	3.223024	-3.090431	-1.479188
C	-0.143595	-2.435711	-0.453645
C	0.922726	-3.328393	-0.508279
C	0.639355	-4.697293	-0.390403
C	-0.676502	-5.130819	-0.227014
C	-1.716734	-4.211050	-0.189958
O	2.654827	1.658459	2.959817
O	1.894957	-3.051791	2.203743
O	1.703306	-5.561990	-0.450812
O	-3.023994	-4.554356	0.009162
O	-1.672209	-2.322501	2.496258
O	-1.034740	2.411839	3.239718
C	-3.670044	-2.601132	-2.205019

C	-4.738948	-2.629780	-3.030267
C	-5.942082	-1.844500	-2.665715
C	-5.869814	-0.949095	-1.490068
C	-4.749287	-0.943131	-0.735122
C	-2.471387	2.857106	-1.749521
C	-3.364067	2.967663	-2.811193
C	-4.561256	3.670715	-2.575939
C	-4.878054	4.223454	-1.319448
C	-3.968374	4.004357	-0.280843
C	3.764616	-4.261998	-3.571684
C	5.155870	-3.762448	-3.478116
C	5.520836	-2.891075	-2.334754
C	4.579720	-2.591704	-1.412631
C	3.968040	2.098755	-1.094463
C	5.306549	2.453155	-1.146909
C	6.042728	2.411692	-2.332050
C	5.419907	1.827128	-3.447633
C	4.055527	1.462834	-3.441436
C	3.344633	1.679433	-2.263471
C	-7.082305	-0.086659	-1.130879
O	-7.003544	-1.955188	-3.331208
C	-4.747788	-3.461215	-4.317429
C	6.962138	-2.383707	-2.214543
O	6.008386	-4.073438	-4.347260
C	3.358575	-5.127721	-4.769249
C	-3.055084	2.305656	-4.164145
O	-5.409671	3.800101	-3.669458
C	-6.137492	5.089830	-1.091024
C	7.479515	2.971988	-2.348061
O	6.230349	1.609894	-4.555181
C	3.358290	0.812151	-4.655508
C	-6.785999	0.825682	0.074221
C	-5.856794	-4.536933	-4.256091
C	7.922765	-3.585880	-2.068354
C	4.209639	-6.417795	-4.795796
C	3.378033	1.758626	-5.880888
C	8.478380	1.811208	-2.173875
C	-6.245198	5.559138	0.373717
C	-4.142349	1.253371	-4.480909
C	-4.977564	-2.533448	-5.532496
C	7.344472	-1.551664	-3.458017
C	3.553582	-4.334080	-6.080490
C	4.005669	-0.560023	-4.976953
C	7.683829	3.974464	-1.188089
C	-2.982711	3.365925	-5.284917

C	-7.451943	0.815490	-2.329627
C	-3.405805	-4.185802	-4.525972
C	7.142654	-1.482938	-0.979717
C	1.878823	-5.547631	-4.688808
C	1.872104	0.512637	-4.364816
C	7.801122	3.746239	-3.645881
C	-1.701571	1.567330	-4.138657
C	-8.278087	-0.992705	-0.758954
C	-6.048898	6.367126	-1.961758
C	-7.445808	4.313035	-1.374650
C	-2.481627	-2.630229	3.687442
C	-0.199110	2.201068	4.409715
C	1.259627	-3.250668	3.492606
C	3.753983	1.557795	3.947746
C	5.094227	1.614086	3.272241
C	2.179931	-2.973903	4.655433
C	-0.904357	1.479521	5.530490
C	-3.930096	-2.295513	3.474955
C	-4.556571	-1.324974	4.256001
C	-5.895800	-1.007462	4.040346
C	-6.610951	-1.654543	3.035754
C	-5.981461	-2.610620	2.239099
C	-4.646205	-2.928001	2.456005
C	-0.210237	0.544618	6.300945
C	-0.858615	-0.146411	7.321607
C	-2.208124	0.086955	7.575031
C	-2.903506	1.021342	6.809163
C	-2.254196	1.714594	5.791903
C	3.563954	-3.090807	4.524267
C	4.399464	-2.794046	5.597034
C	3.858796	-2.386376	6.814945
C	2.477347	-2.277531	6.953860
C	1.642728	-2.565388	5.876623
C	5.644074	0.462771	2.698924
C	6.849243	0.526679	2.007264
C	7.523697	1.739570	1.891797
C	6.987320	2.890367	2.466291
C	5.775830	2.827127	3.149943
C	-3.452360	-5.931512	0.150947
C	1.605458	-6.899492	0.124323
C	4.333593	5.223142	0.873690
C	-1.579994	6.367967	-1.152176
C	3.605207	5.865852	2.018515
C	-4.949790	-5.881575	0.286600
C	1.556535	-6.861254	1.627899

C	-5.616528	-6.654578	1.234657
C	-7.005481	-6.595060	1.336057
C	-7.734062	-5.758002	0.495709
C	-7.069122	-4.976455	-0.448839
C	-5.684043	-5.038010	-0.549843
C	2.659063	-6.378936	2.339110
C	2.644265	-6.354806	3.728276
C	1.516468	-6.792371	4.422641
C	0.409102	-7.259731	3.721238
C	0.432480	-7.296824	2.328033
C	3.615637	7.252265	2.177467
C	2.908928	7.851137	3.216537
C	2.178427	7.065287	4.104075
C	2.155224	5.681529	3.946709
C	2.861964	5.082964	2.909058
C	-2.531022	7.505339	-0.909623
C	-2.853849	8.361107	-1.965636
C	-3.754235	9.404475	-1.777470
C	-4.338756	9.606008	-0.528376
C	-4.013538	8.759644	0.527181
C	-3.114139	7.711825	0.338471
H	1.862293	-4.268597	-2.660934
H	1.333239	6.384851	-0.290146
H	0.525071	1.610635	0.565465
H	3.891383	2.559198	0.970448
H	-2.147594	3.724978	1.511816
H	1.966888	-0.747053	3.649139
H	3.032104	-0.282771	-1.125635
H	-2.605390	0.802844	-0.641637
H	-0.960410	-0.220489	3.872831
H	0.066414	-1.374511	-0.502646
H	-0.889050	-6.181462	-0.109840
H	-2.784791	-3.174305	-2.439379
H	-4.700338	-0.342259	0.159974
H	-1.516411	2.373422	-1.899836
H	-4.174991	4.395777	0.704339
H	4.829067	-1.978357	-0.559825
H	5.779897	2.798279	-0.238954
H	2.282418	1.482597	-2.231885
H	-6.254503	4.211013	-3.440662
H	5.761837	1.181056	-5.283983
H	-5.963321	1.518041	-0.137380
H	-7.679388	1.418073	0.295665
H	-6.542977	0.250384	0.972471
H	-5.678804	-5.225543	-3.423561

H	-6.834828	-4.074233	-4.129255
H	-5.854405	-5.117276	-5.185258
H	7.872886	-4.228795	-2.946933
H	8.950313	-3.224004	-1.953659
H	7.665134	-4.174639	-1.181338
H	5.268089	-6.180507	-4.895189
H	4.058265	-6.990190	-3.874262
H	3.904617	-7.043154	-5.641928
H	4.378760	2.069087	-6.191471
H	2.901599	1.269590	-6.736088
H	2.819948	2.670102	-5.648771
H	8.290869	1.287545	-1.231289
H	8.378672	1.094431	-2.991558
H	9.506051	2.192359	-2.163937
H	-5.386543	6.169066	0.660549
H	-6.333811	4.713560	1.062502
H	-7.142915	6.175264	0.481448
H	-5.125754	1.715164	-4.563388
H	-3.906868	0.755951	-5.428035
H	-4.177397	0.491960	-3.693952
H	-4.968509	-3.122243	-6.456279
H	-4.179599	-1.786360	-5.593769
H	-5.935521	-2.019846	-5.452363
H	8.393940	-1.246507	-3.383666
H	6.735196	-0.646191	-3.518058
H	7.214852	-2.134280	-4.369251
H	3.263774	-4.954338	-6.935664
H	4.594222	-4.033234	-6.199842
H	2.922822	-3.438805	-6.077237
H	3.905836	-1.225792	-4.114831
H	5.069930	-0.518576	-5.219605
H	3.493016	-1.019096	-5.827729
H	8.667669	4.442123	-1.291333
H	7.654848	3.493993	-0.208166
H	6.922493	4.760439	-1.213415
H	-2.751649	2.879695	-6.239128
H	-2.189182	4.089933	-5.069626
H	-3.928063	3.898052	-5.380365
H	-8.331954	1.417182	-2.077667
H	-7.677598	0.215732	-3.210815
H	-6.627412	1.496530	-2.563496
H	-2.572222	-3.481383	-4.615877
H	-3.189831	-4.882566	-3.708720
H	-3.456413	-4.763298	-5.453728
H	8.179724	-1.135908	-0.943022

H	6.498344	-0.597856	-1.020913
H	6.938451	-2.028978	-0.052179
H	1.639874	-6.173120	-5.554084
H	1.673265	-6.130060	-3.784549
H	1.208938	-4.681547	-4.707822
H	1.421986	0.060226	-5.253388
H	1.756160	-0.194175	-3.537273
H	1.311547	1.422952	-4.135089
H	8.774147	4.236494	-3.535730
H	7.830352	3.087862	-4.509368
H	7.047979	4.520194	-3.829686
H	-1.529909	1.111219	-5.118333
H	-0.869047	2.249729	-3.939943
H	-1.688331	0.766507	-3.391552
H	-9.138128	-0.374563	-0.478463
H	-8.557035	-1.625715	-1.601113
H	-8.020393	-1.628990	0.094389
H	-5.171052	6.949015	-1.674277
H	-5.966911	6.158293	-3.031522
H	-6.939043	6.985023	-1.806013
H	-8.302242	4.970154	-1.194295
H	-7.521063	3.454057	-0.705749
H	-7.548050	3.926047	-2.391844
H	-2.318067	-3.698649	3.826963
H	-2.080270	-2.092718	4.553095
H	0.069167	3.215966	4.705662
H	0.712302	1.680221	4.101783
H	0.957574	-4.297768	3.467171
H	0.358457	-2.634136	3.537844
H	3.589899	2.410429	4.604532
H	3.630185	0.630953	4.516008
H	-3.992855	-0.810164	5.026807
H	-6.376610	-0.252831	4.651000
H	-7.653012	-1.409660	2.866581
H	-6.523812	-3.109304	1.446025
H	-4.151123	-3.645860	1.813486
H	0.835976	0.342397	6.093297
H	-0.312755	-0.870426	7.914440
H	-2.715325	-0.454901	8.363563
H	-3.952941	1.207930	7.003307
H	-2.797313	2.424930	5.180556
H	3.979909	-3.393967	3.571486
H	5.473306	-2.877485	5.481943
H	4.509603	-2.153079	7.648477
H	2.051347	-1.960687	7.898390

H	0.568182	-2.455396	5.980853
H	5.126069	-0.484383	2.797334
H	7.266541	-0.368198	1.564251
H	8.466461	1.786022	1.360431
H	7.511121	3.834813	2.383002
H	5.354875	3.724701	3.589521
H	-2.983767	-6.385574	1.030281
H	-3.149902	-6.491389	-0.741934
H	2.515088	-7.381831	-0.231153
H	0.750074	-7.436123	-0.294833
H	5.125017	5.865675	0.490389
H	4.778467	4.276663	1.184965
H	-0.685352	6.722259	-1.676479
H	-2.052324	5.590056	-1.762866
H	-5.054037	-7.299067	1.901349
H	-7.516029	-7.196439	2.078179
H	-8.812825	-5.710148	0.579461
H	-7.622542	-4.314413	-1.104493
H	-5.163387	-4.411728	-1.259786
H	3.525023	-6.019336	1.796391
H	3.502551	-5.982517	4.272487
H	1.503044	-6.764739	5.505229
H	-0.469701	-7.599404	4.255379
H	-0.427048	-7.676230	1.785911
H	4.180230	7.864877	1.483060
H	2.925560	8.927991	3.331546
H	1.626565	7.529556	4.912314
H	1.581715	5.068427	4.630900
H	2.815222	4.006840	2.776435
H	-2.400882	8.208277	-2.939614
H	-3.998492	10.060401	-2.604244
H	-5.039496	10.418545	-0.380204
H	-4.461688	8.911713	1.501915
H	-2.852437	7.051595	1.153691

Supplementary Table 8. Cartesian coordinates for the DFT calculated structure of ‘gem’-rccc-**2**-[Ox_2]

300
ox2c-wb97xd-431g-s.log (E=-6316.72437112)

C	-0.411139	4.821487	0.954494
C	0.416411	-3.375482	-1.956090
C	1.340590	-2.941502	-2.891281
C	2.403380	-2.149292	-2.489208
C	2.532382	-1.731727	-1.166608
C	1.576420	-2.170482	-0.247956
C	0.525996	-3.010961	-0.607909
C	3.712879	-0.855800	-0.807309
C	-0.451242	-3.603794	0.398482
O	3.367205	-1.820110	-3.448243
O	-0.630034	-4.221590	-2.295660
C	0.080907	-3.626972	1.828915
C	-1.829196	-2.978425	0.287789
C	3.449820	0.618027	-1.039885
C	4.551034	1.438173	-1.321767
C	4.412128	2.812821	-1.449908
C	3.162139	3.391465	-1.250850
C	2.046568	2.603768	-0.938419
C	2.213830	1.214822	-0.885150
C	-2.002897	-1.598937	0.223306
C	-3.260359	-0.998737	0.119389
C	-4.381253	-1.840153	-0.009808
C	-4.237301	-3.211776	0.127189
C	-2.977724	-3.772120	0.290039
C	-3.380584	0.471408	0.051092
C	0.754774	3.185777	-0.495674
C	-4.297587	1.163170	0.799211
C	-2.409293	1.145502	-0.839952
C	0.744336	4.043255	0.574466
C	-1.448088	2.016659	-0.322582
C	-0.449543	2.596579	-1.120288
C	-0.518030	2.356971	-2.504718
C	-1.433394	1.455883	-3.027654
C	-2.340746	0.816804	-2.202246
O	5.745429	0.781733	-1.453139
O	2.958038	4.756330	-1.345595
O	0.296209	2.967881	-3.431846
O	-3.192802	-0.161585	-2.673513
O	-5.593499	-1.259815	-0.324290
O	-2.900497	-5.148517	0.482683

C	-4.947131	0.535917	1.932568
C	-5.866867	1.159344	2.699442
C	-6.325947	2.508537	2.302029
C	-5.696040	3.162366	1.129798
C	-4.693950	2.523182	0.479431
C	1.265736	-4.311483	2.094858
C	1.755011	-4.482184	3.384570
C	0.992956	-3.939995	4.440595
C	-0.192593	-3.218914	4.215820
C	-0.617027	-3.078006	2.891201
C	-0.391920	5.697715	1.986830
C	0.834636	5.836092	2.807223
C	2.021928	5.040657	2.434460
C	1.938178	4.213093	1.374411
C	4.284294	-1.068507	0.586187
C	5.480378	-1.760742	0.722242
C	6.107029	-1.933167	1.953222
C	5.474518	-1.374907	3.079336
C	4.261269	-0.662989	2.988408
C	3.695156	-0.525331	1.719871
C	-6.226845	4.539021	0.701983
O	-7.228804	3.091730	2.951586
C	-6.401796	0.548176	4.001685
C	3.301995	5.149554	3.269511
O	0.863557	6.607755	3.798985
C	-1.593928	6.583439	2.321722
C	3.045448	-5.282918	3.635429
O	1.479777	-4.176224	5.722529
C	-1.046941	-2.639596	5.363822
C	7.457285	-2.664728	2.046918
O	6.140159	-1.537562	4.290851
C	3.581026	-0.023241	4.217972
C	-5.461466	5.082896	-0.518397
C	-5.972601	1.447526	5.185346
C	3.805619	6.610800	3.268352
C	-1.199924	8.072115	2.191921
C	3.153284	-1.107245	5.235532
C	8.540422	-1.687243	2.556131
C	-2.292493	-1.900766	4.832495
C	4.071509	-4.446061	4.426833
C	-7.943158	0.434554	3.979311
C	3.023747	4.682945	4.715912
C	-2.100899	6.286202	3.751403
C	4.515184	1.018551	4.879803
C	7.917575	-3.182665	0.670185

C	2.703630	-6.576129	4.408545
C	-7.718833	4.439772	0.303181
C	-5.818605	-0.854616	4.249908
C	4.429498	4.271211	2.696521
C	-2.752819	6.319254	1.355590
C	2.285060	0.719874	3.829609
C	7.349250	-3.886143	2.984919
C	3.727544	-5.688711	2.313244
C	-6.106450	5.547973	1.871262
C	-1.577331	-3.781699	6.267046
C	-0.251542	-1.594562	6.183880
C	-6.630889	-2.094038	-0.914461
C	-2.826561	-5.998909	-0.727936
C	4.080168	5.666476	-1.213130
C	6.988141	1.516655	-1.594464
C	8.083947	0.490190	-1.632210
C	4.930462	5.749546	-2.456638
C	-3.951466	-5.771726	-1.697973
C	-7.413601	-2.857120	0.121009
C	-7.854331	-4.154312	-0.136680
C	-8.589933	-4.847492	0.821623
C	-8.880050	-4.250644	2.045383
C	-8.434320	-2.956089	2.307450
C	-7.707973	-2.261339	1.346448
C	-3.860081	-4.742235	-2.639579
C	-4.895335	-4.508183	-3.538017
C	-6.041652	-5.300430	-3.499189
C	-6.146005	-6.322909	-2.557155
C	-5.107714	-6.552537	-1.657502
C	6.312335	5.910987	-2.346443
C	7.104377	5.996359	-3.488479
C	6.519737	5.912656	-4.749215
C	5.140903	5.746958	-4.863111
C	4.349559	5.667591	-3.722143
C	9.153608	0.547261	-0.741213
C	10.168528	-0.406765	-0.796740
C	10.113031	-1.429296	-1.738104
C	9.037943	-1.498603	-2.623842
C	8.030210	-0.542439	-2.572521
C	-3.919016	0.093770	-3.913320
C	0.498822	4.420279	-3.355814
C	4.538248	-2.733696	-3.526967
C	-0.537573	-4.985404	-3.521546
C	4.173094	-4.160914	-3.244605
C	-4.952495	1.168496	-3.726897

C	-0.763743	5.195905	-3.084305
C	-5.090423	2.202139	-4.651139
C	-6.084410	3.165563	-4.483452
C	-6.939720	3.098558	-3.386902
C	-6.789387	2.073726	-2.452961
C	-5.795915	1.117909	-2.615416
C	-1.992359	4.820636	-3.634011
C	-3.137311	5.564960	-3.371903
C	-3.058549	6.716456	-2.590813
C	-1.833703	7.105824	-2.054972
C	-0.696607	6.334106	-2.280437
C	4.235327	-4.653586	-1.938790
C	3.768150	-5.929770	-1.642831
C	3.236872	-6.727968	-2.653370
C	3.181947	-6.248851	-3.961354
C	3.645371	-4.969057	-4.252879
C	-0.919666	-4.211663	-4.760664
C	-0.653060	-4.788185	-6.004507
C	-1.015758	-4.137373	-7.178240
C	-1.642204	-2.894899	-7.118582
C	-1.898489	-2.312943	-5.881090
C	-1.542793	-2.965070	-4.702516
H	-1.287411	4.725823	0.328441
H	1.280090	-3.230796	-3.928258
H	1.692086	-1.908548	0.795764
H	4.514831	-1.113487	-1.496320
H	-0.572230	-4.653426	0.114440
H	5.256618	3.428476	-1.715261
H	1.353835	0.595413	-0.658296
H	-1.125807	-0.961943	0.247442
H	-5.088892	-3.872552	0.097548
H	-1.427844	2.189074	0.746842
H	-1.405346	1.274029	-4.091910
H	-4.603403	-0.454306	2.195324
H	-4.217853	2.978042	-0.378886
H	1.809648	-4.723330	1.256406
H	-1.534477	-2.552478	2.683108
H	2.781384	3.592994	1.117763
H	5.953373	-2.133329	-0.174895
H	2.790312	0.052494	1.599847
H	0.937271	-3.766069	6.409570
H	5.576145	-1.348604	5.053704
H	-5.573493	4.425708	-1.385830
H	-5.869967	6.062687	-0.786237
H	-4.396025	5.212938	-0.321813

H	-4.880699	1.515038	5.236260
H	-6.385718	2.450901	5.078615
H	-6.334226	1.017138	6.125527
H	3.061684	7.274965	3.706637
H	4.732642	6.682224	3.847406
H	4.015083	6.938629	2.244024
H	-0.401241	8.325867	2.888325
H	-0.865023	8.286880	1.171044
H	-2.069714	8.702268	2.406558
H	3.972777	-1.705508	5.643060
H	2.648827	-0.634100	6.083235
H	2.461875	-1.807702	4.763114
H	8.640576	-0.846883	1.861636
H	8.289061	-1.305728	3.545582
H	9.506540	-2.201805	2.609687
H	-2.954822	-2.569729	4.275844
H	-2.022802	-1.057046	4.189781
H	-2.856252	-1.506796	5.683280
H	3.670164	-4.143282	5.392186
H	4.980000	-5.036119	4.587010
H	4.347338	-3.555221	3.857756
H	-8.285056	-0.067133	4.891357
H	-8.281725	-0.151584	3.120719
H	-8.399293	1.421229	3.922818
H	3.941574	4.747530	5.310434
H	2.687162	3.641026	4.720486
H	2.258591	5.305710	5.179299
H	-2.981619	6.902592	3.962814
H	-1.330837	6.502449	4.490671
H	-2.393948	5.234268	3.837721
H	4.748068	1.816652	4.171848
H	5.464558	0.597476	5.215889
H	4.016611	1.465751	5.745552
H	8.880065	-3.689987	0.788824
H	8.061638	-2.369343	-0.045987
H	7.208662	-3.904912	0.250861
H	3.615521	-7.160233	4.575505
H	2.002928	-7.189851	3.832748
H	2.255064	-6.342250	5.374370
H	-8.091981	5.439854	0.055479
H	-8.315143	4.039291	1.122788
H	-7.845385	3.806499	-0.577331
H	-6.077463	-1.559609	3.454682
H	-4.729838	-0.822724	4.344214
H	-6.221717	-1.246785	5.188647

H	5.314719	4.366575	3.332313
H	4.153878	3.213212	2.666021
H	4.708883	4.579259	1.684069
H	-3.587380	6.980984	1.599882
H	-2.471909	6.506366	0.316888
H	-3.108138	5.287626	1.438720
H	1.844026	1.155422	4.731484
H	2.471316	1.537513	3.126546
H	1.547622	0.038952	3.392571
H	8.316200	-4.399576	3.031510
H	7.058319	-3.584713	3.990047
H	6.603916	-4.591151	2.602931
H	4.650493	-6.228988	2.545238
H	3.095768	-6.352869	1.714712
H	3.992868	-4.810772	1.713902
H	-6.267087	6.564830	1.496505
H	-6.847535	5.328830	2.638057
H	-5.118088	5.504973	2.331016
H	-2.212929	-4.449269	5.678645
H	-0.792671	-4.397767	6.713021
H	-2.175912	-3.362898	7.081872
H	-0.884984	-1.190582	6.979375
H	0.056671	-0.770518	5.534206
H	0.655389	-1.983564	6.653220
H	-6.183151	-2.769676	-1.651189
H	-7.265703	-1.376273	-1.437459
H	-2.845747	-7.009170	-0.322928
H	-1.865155	-5.806516	-1.204604
H	3.605994	6.623787	-0.992343
H	4.684778	5.373550	-0.348849
H	7.110517	2.200920	-0.748785
H	6.957495	2.102676	-2.520253
H	-7.604516	-4.628748	-1.079040
H	-8.924646	-5.857051	0.616303
H	-9.444069	-4.792725	2.794435
H	-8.648762	-2.489874	3.261631
H	-7.345402	-1.258749	1.539150
H	-2.974019	-4.121510	-2.652673
H	-4.799298	-3.717251	-4.272206
H	-6.847388	-5.126830	-4.202140
H	-7.034336	-6.942379	-2.525710
H	-5.193173	-7.346588	-0.924024
H	6.771664	5.964223	-1.364825
H	8.176059	6.120295	-3.393078
H	7.135145	5.971382	-5.638345

H	4.682501	5.675659	-5.841738
H	3.279727	5.525979	-3.808484
H	9.194445	1.336089	0.001588
H	10.993958	-0.354945	-0.097727
H	10.897653	-2.174694	-1.777566
H	8.987628	-2.296970	-3.354077
H	7.193489	-0.592632	-3.259151
H	-3.229076	0.350038	-4.721133
H	-4.371860	-0.871823	-4.139673
H	0.902439	4.653890	-4.342950
H	1.255316	4.652739	-2.606371
H	5.296936	-2.381698	-2.822229
H	4.906622	-2.594891	-4.542271
H	0.474207	-5.397152	-3.614245
H	-1.234856	-5.813271	-3.372134
H	-4.424987	2.255320	-5.506396
H	-6.189794	3.963967	-5.208162
H	-7.712827	3.845585	-3.253135
H	-7.440002	2.018253	-1.590078
H	-5.653479	0.345626	-1.868000
H	-2.057711	3.934493	-4.252851
H	-4.090435	5.242839	-3.773706
H	-3.948926	7.299752	-2.391655
H	-1.766352	7.998875	-1.445303
H	0.247481	6.612327	-1.824986
H	4.626441	-4.025035	-1.145621
H	3.815183	-6.298205	-0.625588
H	2.870656	-7.721143	-2.423803
H	2.777629	-6.870670	-4.751156
H	3.583533	-4.587288	-5.266125
H	-0.157633	-5.752520	-6.053113
H	-0.804933	-4.595453	-8.136783
H	-1.920948	-2.381109	-8.030255
H	-2.370991	-1.340842	-5.834478
H	-1.733775	-2.494525	-3.745105
